# Low contact resistance and high current density in ballistic p-type transistors based on oxygen-doped WSe_2_ monolayers

**DOI:** 10.1038/s41467-026-78157-w

**Published:** 2026-10-01

**Authors:** Lei Sun, Tingting Gao, Lin Xu, Wanying Li, Chuanqi Zhang, Zhengyu Zhang, Kian Ping Loh, Yuhui He, Kaustav Banerjee, Yang Chai, Lain-Jong Li, Xiangshui Miao, Xuefei Li

**Affiliations:** 1 Wuhan National High Magnetic Field Center, School of Integrated Circuits, Huazhong University of Science and Technology, Wuhan, 430074 China; 2 Department of Applied Physics, The Hong Kong Polytechnic University, Hung Hom, Kowloon, Hong Kong, China; 3 Department of Electrical and Computer Engineering, University of California, Santa Barbara, CA USA; 4 Department of Materials Science and Engineering, National University of Singapore, Singapore, Singapore; 5 Department of Chemistry, National University of Singapore, Singapore, Singapore

**Keywords:** Electronic devices, Electronic devices

## Abstract

The development of high-performance p-type monolayer transistors is critical for extending two-dimensional (2D) semiconductors beyond silicon in sub 1-nm technology nodes for complementary metal–oxide–semiconductor (CMOS) circuits. However, insufficient doping and metal-induced gap states (MIGS) during metal deposition lead to high contact resistance, greatly limiting p-type on-state currents. Here, we demonstrate high-performance ballistic p-type monolayer tungsten diselenide (WSe_2_) transistors by oxygen p-doping. The controllable oxygen doping greatly reduces the number of Se vacancy defects in as-grown WSe_2_, and thus increases the hole mobility from 55 to 218.3 cm^2^ V^−1^ s^−1^—approaching the theoretical limit. Moreover, this approach also induces a heavily hole-doped contact region in p-type monolayer WSe_2_ devices and decreases the contact resistance from 1869 Ω·μm down to 104 Ω·μm. Consequently, a 16-nm-channel WSe_2_ p-type field effect transistor (pFET) delivers a saturation current of up to 1635 μA μm^−1^ at a drain voltage of −1.2 V and a ballistic ratio as high as 81% at room temperature.

## Abstract (web summary)

The realization of high-performance p-type transistors is essential for the realization of next-generation 2D electronics. Here, the authors report the realization of oxygen-doped p-type monolayer WSe_2_ transistors with hole mobility over 200 cm^2^ V^−1^ s^−1^, contact resistance down to 104 Ω·μm and saturation current densities up to 1635 μA/μm at a drain voltage of -1.2 V.

## Introduction

In recent years, two-dimensional (2D) layered semiconductors with atom-scale thicknesses have been proposed as potential channel materials to extend Moore’s law in complementary metal-oxide-semiconductor (CMOS) technology^1^. Among them, transition-metal dichalcogenides (TMDs), such as MoS_2_, WSe_2_, and WS_2_, have received significant attention as potential alternatives to silicon because of their ultrathin nature, good stability, and high carrier mobility at the atomic-scale^1,2^. Significant progress has been made in the development of two-dimensional (2D) semiconductors, including wafer-scale material growth^3,4^, high-performance transistors^2,5,6^, and simple integrated circuits^7,8^. However, most studies have focused on n-type field-effect transistors (nFETs), such as MoS_2_^2,9,10^. Insufficient doping and metal-induced gap states (MIGS) during metal deposition lead to high contact resistance^5,9,11,12^, greatly limiting p-type on-state currents^10,13^. To further advance 2D semiconductor electronics, the development of high-performance p-type 2D transistors with ballistic transport is imperative to achieve high-performance 2D CMOS below the 1 nm technology node.

Monolayer tungsten diselenide (WSe_2_) is a promising p-type 2D channel material because of its high thermal velocity (~0.41m_0_ for p-type WSe_2_ versus ~0.69m_0_ for p-type Si) and ultrathin nature (<1 nm)^14^. Moreover, wafer-scale monolayer WSe_2_ grown by chemical vapor deposition (CVD) has been developed, making it a suitable candidate for practical electronic device applications^15^. However, experimentally achieving a high p-type on-state current with ballistic transport has proven exceedingly challenging. The primary obstacle is that WSe_2_ films grown by CVD exhibit high defect densities, such as selenide vacancies^9^. A high selenide-vacancy density strongly scatters carriers and decreases hole mobility^16^. Moreover, conventional metal physical vapor deposition usually leads to metal-induced gap states that cause Fermi-level pinning and high contact resistance (*R*_c_) at the metal/WSe_2_ interface accordingly^13,17^. Using vdW contacts^17,18^, substitutional doping during material synthesis^19^, and surface charge transfer or metal oxide-based doping have been explored to improve the electrical characteristics of WSe_2_ pFETs^20^. However, the contact resistance of monolayer WSe_2_ pFETs is typically one order of magnitude greater than that of metal-MoS_2_ contacts, which significantly limits the p-type on-state current in short-channel transistors^6,21^. Furthermore, ballistic p-type transistors with high on-state current based on monolayer WSe_2_ have yet to be achieved. To further advance 2D CMOS electronics, developing high-performance p-type 2D transistors with ballistic transport is imperative to fully utilize 2D CMOSs below the 1-nm technology node.

Here, we demonstrate ballistic monolayer WSe_2_ pFETs by repairing channel defects and reducing contact resistance using an oxygen doping approach. Combined with a full-contact structure, monolayer WSe_2_ pFETs using high-work-function Pt metal exhibit nearly ohmic (barrier-free) contacts and achieve a low contact resistance of 104 Ω·μm. Ultrashort channel monolayer WSe_2_ pFETs with a channel length (*L*_ch_) of 16 nm achieve a high-saturation on-state current of 1635 μA μm^−1^ at a drain voltage (*V*_ds_) of −1.2 V and a room-temperature ballistic ratio of 81%.

## Results and Discussion

### Theoretical simulation of selenium vacancy passivation by O_2_

**Fig. 1:**
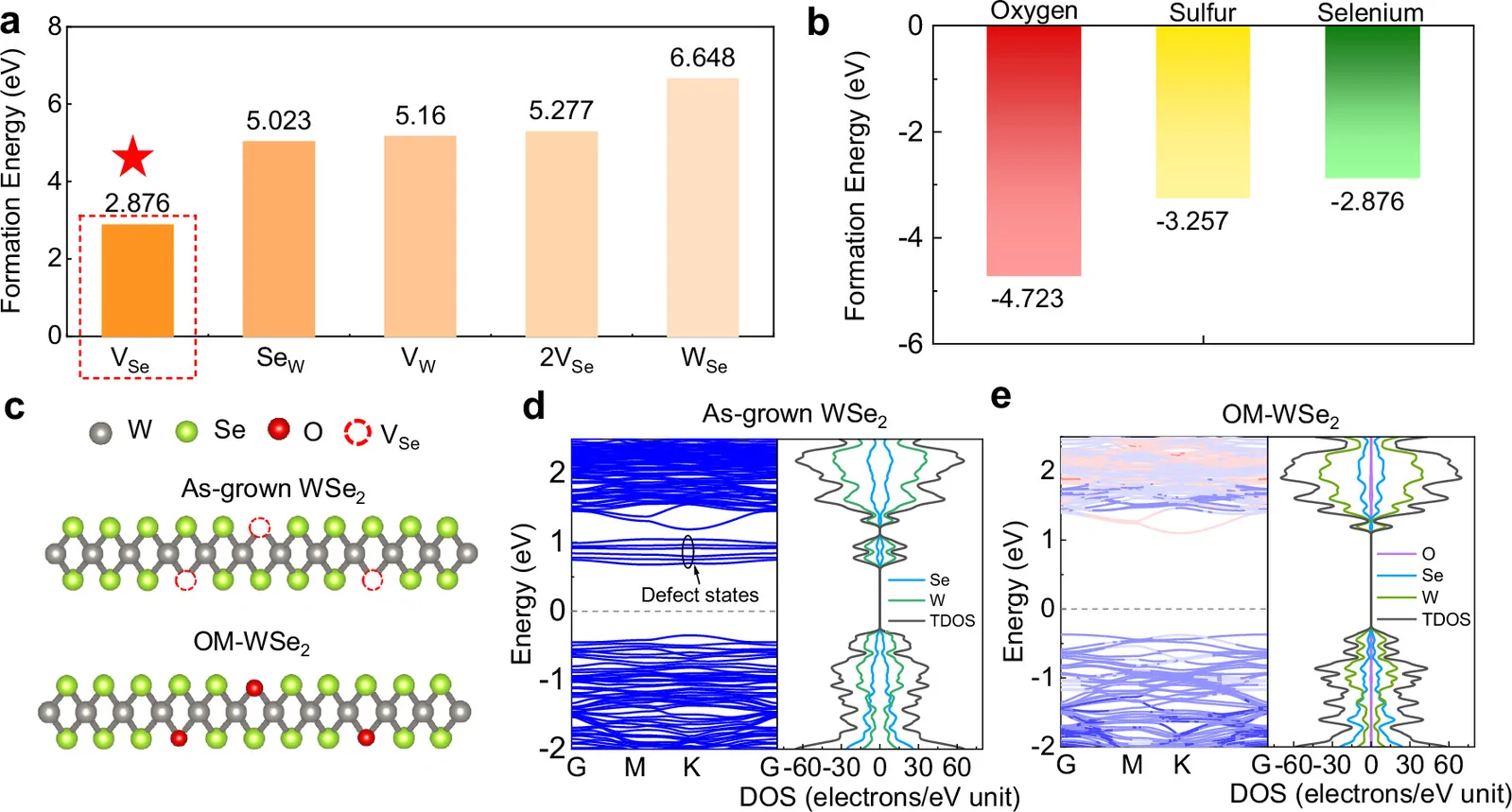
Theoretical calculation of the oxygen passivation of Se vacancies in monolayer WSe_2_. **a** Formation energy of the main types of defects in WSe_2_. V_Se_: Se vacancies, Se_W_: Se substituting W, V_W_: W vacancies, 2V_Se_: double Se vacancies, W_Se_: W substituting Se. **b** Formation energy of passivating Se vacancies using three different types of atoms: O, S, and Se. **c** The top panel is the side view of as-grown WSe_2_ with Se vacancies structures and the bottom panel is the passivated WSe_2_ by oxygen. **d** Band structure and density of states (DOS) of as-grown WSe_2_ with a V_se_ concentration of 4.29 × 10^13^ cm^−2^. **e** Band structure and DOS of oxygen-moderate conditions (OM-WSe_2_) when all V_se_ defects are passivated. The monolayer WSe_2_ structure in the simulation is a 5 × 5 supercell.

In WSe_2_ films grown by CVD, various point defects, including vacancies, antisites, adatoms, and interstitials, are present. The formation energies of several main defects, including Se vacancies (V_Se_), double Se vacancies (2V_Se_), W vacancies (V_W_), W substituting Se (W_Se_), and Se substituting W (Se_W_), were calculated via density functional theory (DFT). The formation energy of V_Se_ was the lowest (~2.9 eV), as shown in Fig. 1a. This indicates that the main point defect in monolayer WSe_2_ should be V_Se_, which is also consistent with the result of previous theoretical and experimental studies^22,23^. Because high defect densities can degrade the electrical properties of WSe_2_ transistors, new methods to reduce selenium vacancy density are urgently needed. The formation energy for passivating V_Se_ with Se, S, and O is shown in Fig. 1b to compare the doping effects of these three types of atoms (Supplementary Fig. 1). The formation energy for passivating V_Se_ with O was the lowest (~−4.7 eV). From the perspective of energy, passivation V_se_ by O to decrease the defect density is the best approach. Figure 1c shows the side view of as-grown WSe_2_ with Se vacancies and passivated WSe_2_ under oxygen-moderate conditions (OM-WSe_2_). The band structure and density of states (DOS) of as-grown WSe_2_ and OM-WSe_2_ were calculated and the results are shown in Fig. 1d, e, respectively (Supplementary Fig. 2). There are many defect states contributed by V_Se_ in as-grown WSe_2_ as shown in Fig. 1d^16^. OM-WSe_2_ can effectively passivate these V_Se_ states (Fig. 1e). Indeed, scanning tunneling microscopy (STM) has corroborated this phenomenon, revealing that Se vacancies in as-grown WSe₂ are effectively passivated by oxygen substitution following post-growth oxygen annealing^15,24^ (Supplementary Fig. 3).

### Experimental correlation of defect states and mobility

**Fig. 2:**
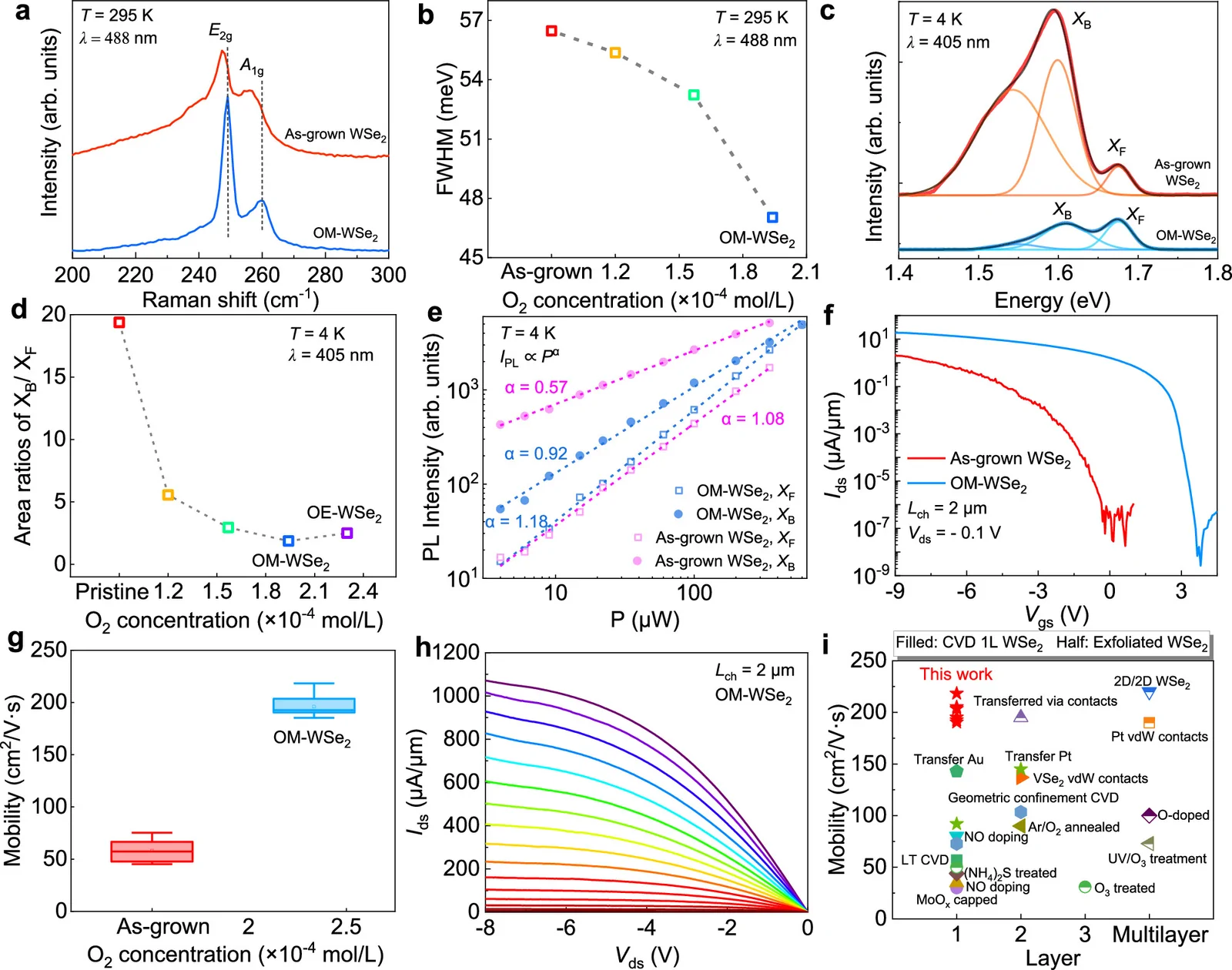
Doping effect of oxygen on monolayer WSe_2_. **a** Typical Raman spectra of these two types of monolayer WSe_2_. **b** Average full-width at half-maximum (FWHM) of the photoluminescence (PL) spectra of monolayer WSe_2_ with and without oxygen doping, showing the increase in crystallinity of OM-WSe_2_. **c** PL spectra of monolayer WSe_2_ with and without oxygen doping at 4 K. Two peaks can be assigned to neutral exciton (*X*_F_) and defect-bound exciton (*X*_B_). **d** Percentage of bound-exciton-associated accumulative PL intensity (*I*_B_/*I*_F_) for different samples. **e** Plot of the intensity of free excitons and bound excitons as a function of the laser power. Lines are power-law fits, and *α* is the power factor. **f** Transfer characteristics of monolayer WSe_2_ devices with and without oxygen doping at a drain voltage (*V*_ds_) of −0.1 V, *I*_ds_ is the drain current, and *L*_ch_ is the channel length. **g** Distribution of the hole field-effect mobility (*μ*_FE_) of monolayer WSe_2_ before and after oxygen doping. In the box plot, the box represents the interquartile range from the 25th to the 75th percentile, and the line inside the box denotes the median. The whiskers extend to the minimum and maximum values, excluding outliers. The mean is denoted by a square within each box. **h** Output characteristics of the monolayer OM-WSe_2_ devices, gate voltage (*V*_gs_) ranges from 4 to −11 V with steps of −1 V. **i** Benchmark of the hole mobility of WSe_2_ with different thicknesses at room temperature^12,17,32–45^.

Monolayer WSe_2_ was grown on 100-nm SiO_2_ substrates via CVD (methods for details of WSe_2_ growth), and the thickness of WSe_2_ was measured to be 0.8 nm via atomic force microscopy (Supplementary Fig. 4). The effects of oxygen doping on the Raman spectrum were studied at room temperature as shown in Fig. 2a (methods for details of the oxygen annealing experiments). The blueshift of both the *A*_1g_ and *E*_2g_ peaks after oxygen doping indicates a p-type doping effect^25,26^. Moreover, significant Raman peak narrowing can be observed for the OM-WSe_2_ sample, suggesting improved crystallinity^27^. With increasing oxygen doping concentration, the average full-width at half-maximum (FWHM) of the photoluminescence (PL) spectrum first decreases from 56.5 to 47.0 meV and then increases to 52.4 meV (Supplementary Fig. 5), indicating that OM-WSe_2_ can improve the crystalline quality^27^. Beyond this observation, we suspect that the as-grown WSe_2_ has an additional p-doping effect on the SiO_2_ growth substrate^28^, as evidenced by the comparative analysis of the Raman and PL spectra acquired from the as-grown and transferred WSe_2_ flakes (Supplementary Fig. 6). Such additional doping of the as-grown WSe_2_ also contributes to the notable electrical characteristics of WSe_2_ devices. To study the effect of oxygen doping on the defect states of monolayer WSe_2_, the temperature dependence of the PL was determined with a 405 nm laser via a confocal PL microscope with ∼1 μm spatial resolution (Supplementary Fig. 7). Figure 2c shows the PL of monolayer WSe_2_ before and after oxygen doping at 4 K. For as-grown WSe_2_, the PL spectra exhibit two peaks: a free excitonic photoluminescence peak (*X*_F_) and a bound excitonic peak (*X*_B_) from localized defect states within the bandgap at lower emission energies^29^. In addition, the peak intensity of *X*_B_ in the as-grown samples is greater than that of the free excitons *X*_F_, indicating a high selenium vacancy density. After oxygen doping, the peak intensity of *X*_B_ decreases greatly, indicating a decreased selenium vacancy density due to oxygen passivation. To further elucidate the effect of oxygen doping, we plot *I*_B_/*I*_F_ as a function of the oxygen concentration (Fig. 2d), where *I*_B_ and *I*_F_ are the accumulated PL intensities for bound excitons and free excitons, respectively. With increasing oxygen concentration, the *I*_B_/*I*_F_ first decreases from 19.4 to 1.9 and then increases (Supplementary Fig. 8). This result shows that the number of defect states decreases by more than ten times for OM-WSe_2_. Therefore, annealing at an appropriate oxygen concentration is very important for reducing the number of defect states and improving the crystallinity of monolayer WSe_2_. To verify that *X*_B_ indeed involves excitons bound to the defect states, we investigated the exciton population as a function of incident laser power ranging from 4 to 600 μW at 4 K via continuous wave excitation at 405 nm (Fig. 2e and Supplementary Fig. 9). By fitting the power-dependent free- and bound-exciton PL emissions via the power law of *I*_PL_  ≈  *P*^α^, where *P* is the incident light power, the power factors *α* of the bound excitons for the as-grown WSe_2_ and OM-WSe_2_ samples are 0.57 and 0.92, respectively. The sublinear trend for *X*_B_ in the as-grown film (*α* = 0.57) confirms the low-energy PL emission from bound excitons^30^. X-ray photoelectron spectroscopy (XPS) revealed that the chemical composition of WSe₂ before and after doping remained unchanged (Supplementary Fig. 10).

To evaluate the quality of monolayer WSe_2_, carrier mobility is an important parameter and is also useful for high-performance field-effect transistors^6^. The transfer characteristics of the as-grown monolayer WSe_2_ on SiO_2_ substrates with and without oxygen doping at a *V*_ds_ of −0.1 V at room temperature are shown in Fig. 2f. Clear p-type transport behaviors with high on/off ratios of 10^8^ were observed for both devices. Moreover, the shift toward a positive gate voltage (*V*_gs_) in the threshold voltage (*V*_th_) indicates p-type doping of oxygen^26^. The gate leakage current of both devices is always less than 2 nA, indicating the excellent insulator properties of SiO_2_ (Supplementary Fig. 11). The peak transconductance value in the linear *V*_ds_ region is used to extract the field-effect hole mobility *μ*_FE_ (The areal capacitance of the gate dielectric (C_ox_) is 0.26 μF/cm^2^. Since the variation in C_ox_ before and after annealing is negligible (as shown in Supplementary Fig. 12), a constant C_ox_ value is adopted for all mobility extractions to ensure a fair comparison.). The extrinsic *μ*_FE_ of as-grown WSe_2_ and OM-WSe_2_ (concentration of O_2_ during annealing 1.94 × 10^−4^ mol/L) are 55 and 218.3 cm^2^ V^−1^ s^−1^, respectively. The corresponding average mobility values are shown in Fig. 2g (Supplementary Fig. 13 for more details). By optimizing the degree of oxygen doping to decrease the defect density, the hole mobility of monolayer WSe_2_ approaches the theoretical limit at room temperature^31^. The corresponding output characteristics of the WSe_2_ transistors are shown in Fig. 2h and Supplementary Fig. 14. For the monolayer OM-WSe_2_ transistors with an *L*_ch_ of 2 µm, we achieve the highest well-saturated drain current of 1070 μA μm^−1^ at a *V*_ds_ of −8 V^31^. Moreover, oxygen doping also reduces the hysteresis window from 0.71 V for as-grown WSe_2_ to ~ 0 V for OM-WSe_2_ (Supplementary Fig. 15). As a reference, the on-state current of WSe_2_ transistors with pure Ar annealing decreases from 63.9 to 47.8 μA μm^−1^, as shown in Supplementary Fig. 16. Figure 2i summarizes the state-of-the-art hole mobility for both monolayer WSe_2_ and thicker WSe_2_ at room temperature^12,17,32–45^ (Supplementary Table 1). The high hole mobility of monolayer WSe_2_ in our work is close to the theoretical value^31^, which is mainly attributed to the effective passivation of selenium vacancies by oxygen doping and a decrease in the defect density in the WSe_2_ film^16^.

### Low contact resistance of WSe_2_ transistors with O_2_ doping

**Fig. 3:**
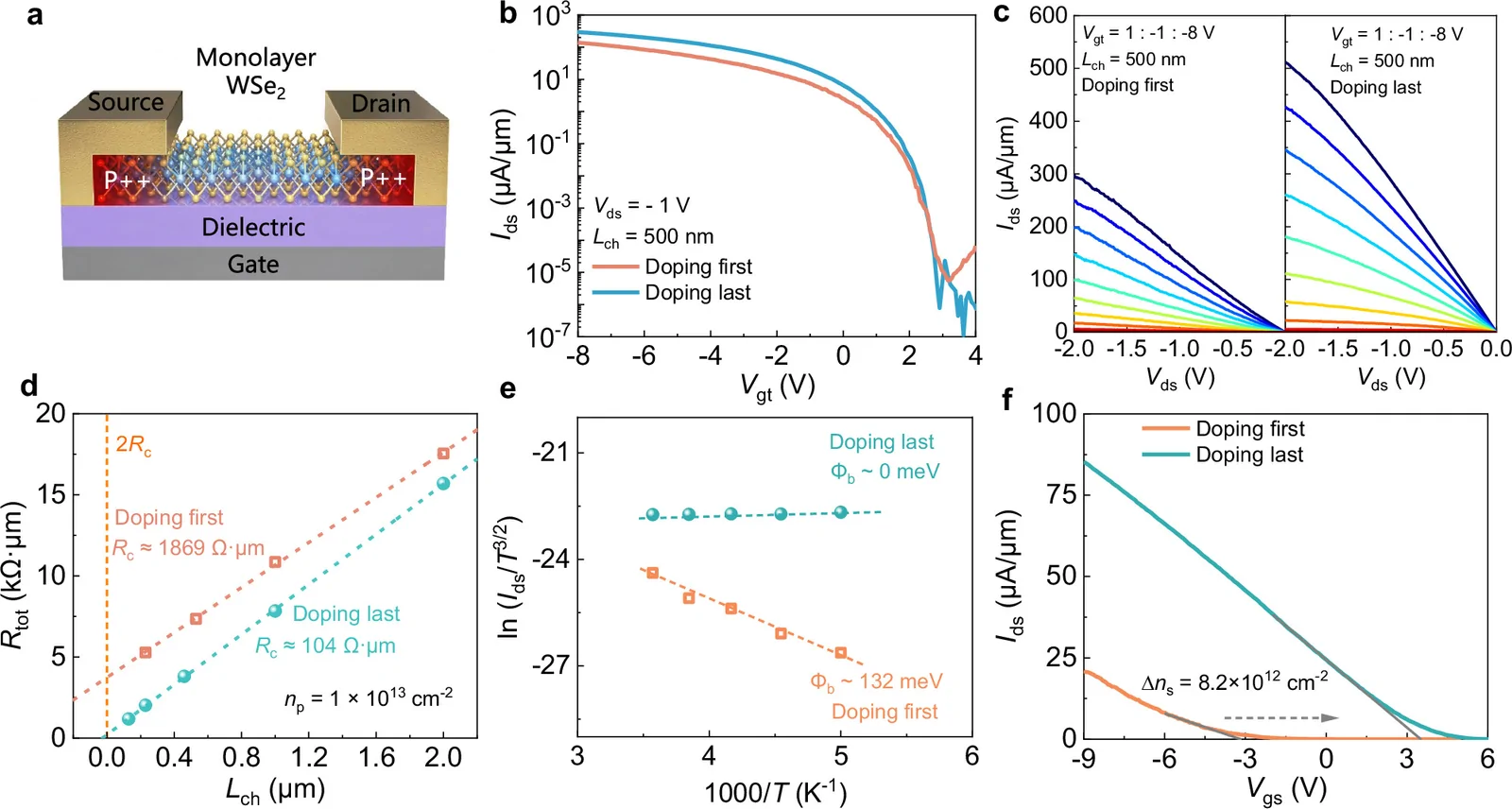
Electrical properties of monolayer WSe_2_ transistors with doping first and doping last. **a** Schematic of monolayer WSe_2_ transistors with doping last by oxygen. **b** Transfer characteristics of monolayer WSe_2_ transistors with doping first and doping last at an *L*_ch_ of 500 nm, *V*_gt_ is the overdrive voltage. **c** Corresponding output characteristics for the same devices in Fig. 3b. **d** Extraction of contact resistance (*R*_c_) from the transfer length method (TLM) structure; total resistance (*R*_tot_) is extracted at *V*_ds_ = −100 mV, and carrier density (*n*_p_) is 1 × 10^13^ cm^−2^. **e** Arrhenius plots of WSe_2_ transistors with doping first and doping last at flatband voltage (1.1 V for doping first and 2 V for doping last), where *T* represents the temperature, and *Φ*_b_ is the Schottky barrier height. The dashed lines indicate the linear relationship of ln(*I*_ds_/*T*^3/2^) versus 1000/*T*. **f** Comparison of the transfer characteristics of monolayer WSe_2_ transistors with doping first and doping last at an *L*_ch_ of 230 nm, indicating an increased hole doping concentration of 8.2 × 10^12^ cm^−2^, where ∆*n*_s_ is the change in the hole carrier density.

The low contact resistance of WSe_2_/metal contacts is necessary for the downsizing of WSe_2_ pFETs with high on-state current. Top contact is widely used to fabricate 2D semiconductor FETs because of its simple process; however, edge contact offers superior electrical characteristics because it is less affected by Fermi-level pinning^13,46^. Here, we utilized a full-contact structure to realize high-quality contacts in monolayer WSe_2_ pFETs by combining the advantages of top and edge contact structures. To reveal the effect of p-type oxygen doping on contact resistance, we fabricated two types of monolayer WSe_2_ devices: the first type of device involved the deposition of contact metals after oxygen doping (doping first) and the other type involved the deposition of contact metals before oxygen doping (doping last) (Fig. 3a and Supplementary Fig. 17). Figure 3a shows a schematic diagram of monolayer WSe_2_ transistors with full contact configurations. The gate dielectric is 15 nm SiO_2_, and the contact metal is Pt deposited by e-beam evaporation. Figure 3b compares typical transfer curves of monolayer WSe_2_ transistors with doping first and doping last at an *L*_ch_ of 500 nm (Statistics data are shown in Supplementary Fig. 18). Clearly, the monolayer WSe_2_ FET with doping last clearly exhibits enhanced p-channel conduction with a high on/off current ratio of >10^8^. Raman and PL spectra confirm oxygen doping in the contact regions, which likely occurs through metal grain boundaries or lateral diffusion from the regions surrounding the contacts (Supplementary Fig. 19). The corresponding output curves at the same overdrive voltage *V*_gt_ (*V*_gt_ = *V*_gs_ - *V*_th_) are shown in Fig. 3c. With doping last by oxygen, the on-state current (*I*_on_) of monolayer WSe_2_ transistors increases from 142.5 to 294.4 μA μm^−1^ at a drain voltage of −1 V, which is mainly attributed to the lower contact resistance. Figure 3d shows the extracted contact resistance of WSe_2_ transistors with doping first and doping last by oxygen using the transfer length method (TLM) (Supplementary Figs. 20 and 21). The contact resistance of monolayer WSe_2_ with doping first is 1869 Ω·μm, which greatly decreases to 104 Ω·μm after doping last at a carrier density of 1 × 10^13^ cm^−2^. To study the Schottky barrier height, we fit the data at *V*_ds_ = −0.1 V to the classical thermionic emission equation of Schottky barriers and the Arrhenius plots of ln(*I*_ds_/*T*^3/2^) versus 1000/*T* for both contacts as shown in Fig. 3e (Supplementary Fig. 22). The Schottky barrier height (*Φ*_b_) of WSe_2_ with doping first at the flatband is 132 meV for a negative slope, whereas this analysis is not applicable to WSe_2_ with doping last because it has a positive slope^5,47^, indicating a near-zero barrier height for hole transport. Moreover, the output curves of monolayer WSe_2_ pFETs with doping last remain linear even at 4 K, providing direct evidence for the realization of a nearly ohmic contact with a negligible Schottky barrier (Supplementary Fig. 23). A linear plot of the transfer curves of short-channel monolayer WSe_2_ transistors with doping first and doping last is shown in Fig. 3f. The added doping concentration is determined to be 8.2 × 10^12^ cm^−2^ using Δ*n*_s_ = *q*·*C*_ox_·Δ*V*_th_, where *q* is the elementary charge, Δ*n*_s_ is the change in the hole carrier density, and Δ*V*_th_ is the change in the threshold voltage.

### Ballistic transport of monolayer WSe_2_ transistors

**Fig. 4:**
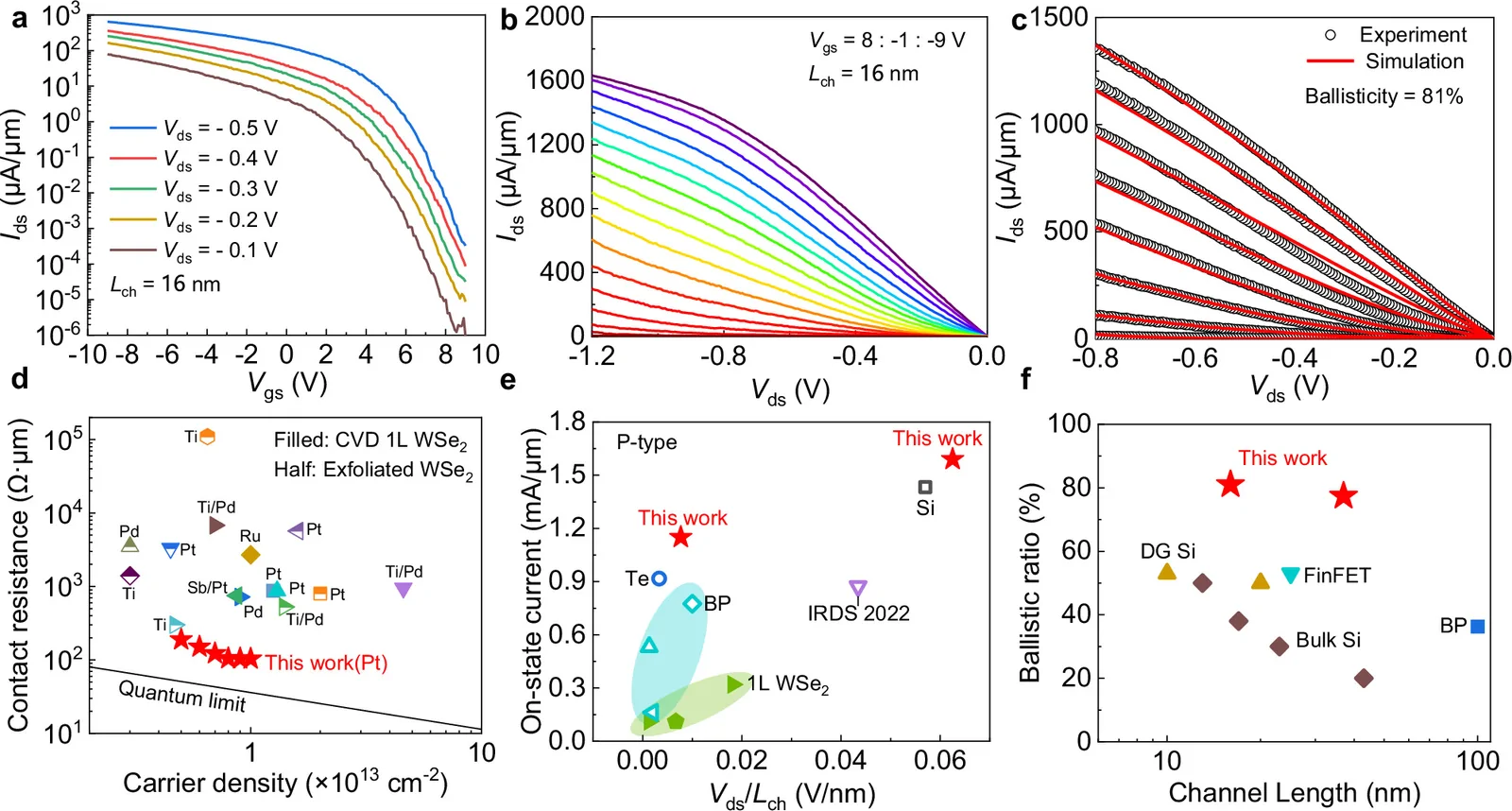
Short-channel and benchmark p-type monolayer WSe_2_ transistors with other devices. **a** Room-temperature transfer characteristics of a typical 16-nm monolayer WSe_2_ p-type field effect transistor (pFET) on 15-nm-thick SiO_2_ dielectrics. **b** Corresponding saturation output characteristics for the device in Fig.4a. **c**, Comparison of the virtual source (VS) model fitting (red lines) and measured data (black lines) for output characteristics from a typical 16-nm-channel-length monolayer WSe_2_ FET. **d** *R*_c_ as a function of carrier density for monolayer WSe_2_ in the literature. The black solid line represents the quantum limit for *R*_c_^12,17,33,34,36–38,42,45,49–54^. **e** The highest on-state current obtained from various p-type materials and devices^12,45,55–61^. **f** Benchmarking the room-temperature ballistic ratios of our WSe_2_ pFETs with those of short-channel FETs, including bulk silicon FETs, silicon FinFETs, dual-gated silicon FETs, and other 2D pFETs^59,62–64^.

To further evaluate the potential of WSe_2_ pFETs with doping last, we scaled the channel length down to 16 nm on monolayer WSe_2_, whose channel length was confirmed by high-resolution cross-sectional transmission electron microscopy (TEM) (Supplementary Fig. 24). Figure 4a demonstrates its transfer characteristics with a high on/off ratio (~10^8^) at room temperature. The threshold voltage can be tuned by separate doping with an appropriate oxygen concentration or by a dual-gated structure, and a high subthreshold swing can be improved by using HfO_2_ dielectrics (Supplementary Fig. 25). The corresponding output characteristics are shown in Fig. 4b, which reveals a high saturation current of 1635 μA μm^−1^ at *V*_ds_ = −1.2 V (1532 μA μm^−1^ at *V*_ds_ = −1.0 V). This current is also higher than that of n-type two-dimensional transistors^48^, which is likely attributed to the high hole mobility of the WSe_2_ channel and low contact resistance. The total resistance in our devices is corrected by the external metal line resistance as shown in Supplementary Fig. 26. An on-state resistance of 99.5 Ω·μm is extracted in the 16-nm WSe_2_ pFETs, which is also close to the value extracted by the TLM (Supplementary Figs. 26 and 27). Moreover, the monolayer WSe_2_ transistor with doping last exhibited excellent stability (Supplementary Fig. 28). Figure 4c shows the modeling results of the output characteristic curves at room temperature with the standard ballistic Virtual Source Model, which are in good agreement with the experimental data. The extracted ballistic efficiency of the fabricated 16-nm device is as high as 81 %. The presence of a metal-to-insulator transition (MIT) at a low carrier density of 1.4 × 10^12^ cm^−2^ indicates a low defect density after oxygen doping (Supplementary Fig. 29). Channel-length scaling shows reduced scattering as the device dimensions shrink (Supplementary Fig. 30).

Figure 4d shows a comparison of the contact resistance for monolayer WSe_2_ pFETs^12,17,33,34,36–38,42,45,49–54^ (Supplementary Table 1). When doping last by oxygen, the high-work-function Pt contact with the full contact structure to monolayer WSe_2_ yields the lowest contact resistance of 104 Ω·μm for pFETs, which approaches the quantum limit. The most meaningful metric for transistor performance is the current density^6^. To make a fair comparison, we plot the on-state current of monolayer 2D materials and conventional semiconductors as a function of *V*_ds_/*L*_ch_, which is essentially the average lateral electric field along the channel (Fig. 4e and Supplementary Fig. 31)^12,45,55–61^ (Supplementary Table 1). It is clear that we achieve the highest hole drain current under the same electric field, suggesting the great potential of 2D materials toward next generation high-performance transistor technologies. Figure 4f shows the benchmark ballistic ratios of our monolayer WSe_2_ pFETs with those of silicon MOSFETs, including 2D pFETs, bulk Si FET, FinFET, and double-gate (DG) Si FETs^59,62–64^. The ballistic ratio of our typical 16 nm WSe_2_ pFET is significantly higher than those of all the previously reported silicon-based and other p-type 2D transistors. The excellent overall electrical performance observed in our ballistic WSe_2_ pFETs originates from minimized contact resistance, which is enabled by doping last and a full contact structure.

In conclusion, our work resolves the critical challenge of low contact resistance in monolayer WSe₂ pFETs through tunable oxygen doping and full contact geometry. The monolayer WSe_2_ transistors achieve low contact resistance (104 Ω·μm) and ballistic transport (81% ratio) with a high saturation current (1635 μA μm^−1^) at room temperature, outperforming state-of-the-art silicon transistors. We also find that the electrical characteristics of WSe_2_ are superior on SiO_2_; therefore, more effort is needed to develop suitable high-k dielectrics that impose a similar doping effect while lowering the EOT. Our results demonstrate that the expected p-type performance of monolayer WSe_2_ is achievable in high-performance 2D p-type electronics for sub-1-nm CMOS technology.

## Methods

### WSe_2_ growth by CVD

Monolayer WSe_2_ was grown on SiO_2_ substrates by a molten-salt-assisted low-pressure chemical vapor deposition (LPCVD) process. Se (sigama-Aldrich, ≥99.5%) powder was prepared in a ceramic boat and placed in downstream of a vacuum one-inch tube furnace, WO_2_ (Alfa Aesar, 99.99%) and KCl (sigama-Aldrich, 99.999%) were prepared in a corundum boat with the substrate above and placed in upstream of the tube. Afterward, the crystals were grown at 890 °C for 10 min under low pressure (1700 Pa) with a mixture of 100 standard cubic centimeters per minute (sccm) Ar and 9 sccm H_2_ as the carrier gas. After the growth, the furnace was naturally cooled to room temperature.

### Device fabrication

For back-gate device fabrication, both WSe_2_ materials grown directly on SiO_2_ and those transferred from sapphire to HfO_2_ were patterned by electron-beam lithography (EBL). AR-P 6200.13 e-beam resists are used during the plasma etching of WSe_2_ films. The channel was subsequently isolated via reactive-ion-etch (SHL 100μ-RIE) with a 100/10/80 sccm flow of Ar/O_2_/CF_4_ gas at 3 W for 180 s, followed by immersing the sample in N-Methylpyrrolidone (NMP) to remove the e-beam resist AR-P 6200.13. After annealing at 250 °C for 2 h with 100 sccm Ar to remove AR-P residuals, S/D contacts were defined by EBL on the PMMA A4 resist and formed with metal deposition by e-beam evaporation. The Pt S/D electrode was deposited under a vacuum of 5e-7 Torr at a rate of 0.3 Å/s. The source-substrate distance is about 65 cm. After the metal was lifted from unwanted regions with acetone, the p-type WSe_2_ devices were annealed in a furnace with a 3 in. quartz tube with an O_2_ atmosphere under different oxygen concentrations for 2 h at 200 °C. The oxygen concentration *C* = *n*/*V* = *P*/*RT* was derived by the ideal gas law *PV* = *nRT*, *P* is the pressure within the chamber, *V* is the volume of the chamber, *n* is the amount of gas, *R* is the universal gas constant (8.314 J/(mol·K)) and *T* is the absolute temperature. Oxygen was introduced from the beginning of the heating process until the entire annealing process was completed, and the supply was stopped when the sample cooled.

### Characterization and measurements

Monolayer WSe_2_ was confirmed using Raman and photoluminescence spectra measurements at a laser wavelength of 405 nm. Raman spectroscopy was obtained through the Via Reflex manufactured by Renishaw. For temperature-dependent PL, a Montana Instruments Cryo Advance was used to control the temperature, and a Keithley 2450 was used to provide the gate voltage. Raman and photoluminescence spectra of WSe_2_ at room temperature were collected in a confocal Raman microscopy system (WITec alpha 300 R) with a 488 nm laser. The test light path was homemade and all the PL spectra at low temperature were acquired in a vacuum environment. AFM was carried out with an SPM-9700 in dynamic mode. SEM images were obtained with a Raith 150-Two. The as-grown monolayer WSe_2_ and oxygen-doped WSe_2_ were transferred on the HOPG substrate by the commonly used PMMA method in DI water. The samples were degassed at 160 °C under the base pressure of ~1 × 10^−10^ mbar. STM/STS measured under ultra-high vacuum conditions using electrochemical etching of tungsten tips. Constant current mode was used during scanning and the tunneling current was 300 pA. Electrical characteristic measurements were performed at a Lakeshore probe station at room temperature under vacuum (<10^−5^ Torr) using an Agilent B1500A semiconductor parameter analyzer.

### DFT calculations

All of the calculations are performed in the framework of the spin-polarized density functional theory with the projector augmented plane-wave method, as implemented in the Vienna ab initio simulation package (VASP)^65,66^. The generalized gradient approximation (GGA) proposed by Perdew, Burke, and Ernzerhof (PBE) is selected for the exchange-correlation potential^67,68^. The long-range van der Waals interaction is described by the DFT-D3 approach^69^. The cut-off energy for the plane wave is set to 480 eV. The energy criterion is set to 10^−5^ eV in the iterative solution of the Kohn-Sham equation. All the structures are relaxed until the residual forces on the atoms have declined to less than 0.02 eV/Å. Data analysis and visualization are carried out with the help of VASPKIT code and VESTA^70,71^. To avoid interlaminar interactions, a vacuum spacing of 20 Å is applied perpendicular to the slab. The formation energies (${E}_{{form}}$) are calculated as follow:

1 $${E}_{{form}}={E}_{{defect}}-{E}_{{perfect}}-{n}_{{defect}}\times {\mu }_{{defect}}$$

where ${E}_{{defect}}$ and ${E}_{{perfect}}$ are the energy with and without the defect. ${\mu }_{{defect}}$ (${n}_{{defect}}$) is the chemical potentials (numbers) of defect atom, respectively.

### Virtual Source Model

To explore the contact resistance and current density, we employ a two-probe model of metal-WSe_2_ contact. The lengths of the left (Pt) and right electrodes (WSe_2_) are semi-infinite, and the center scattering region is the Pt/WSe_2_ contact interface. The transport properties are calculated by the DFT coupled with the non-equilibrium Green’s functions (NEGF) method as implemented in the Quantum ATK package. The transmission coefficient ${T}^{{k}_{//}}\,(E)$ is calculated as

2 $${T}^{{k}_{//}}\left(E\right)={Tr}[{\varGamma }_{L}^{{k}_{//}}\left(E\right){G}^{{k}_{//}}\left(E\right){\varGamma }_{R}^{{k}_{//}}\left(E\right){G}^{{k}_{//}{\dagger} }\left(E\right)]$$

where ${k}_{//}$ is the reciprocal lattice vector point along a surface-parallel direction, ${G}^{{k}_{//}}$ is the retarded Green′s function, ${\varGamma }_{L/R}^{{k}_{//}}(E)=i({\sum }_{L/R}^{r,{k}_{//}}-{\sum }_{L/R}^{a,{k}_{//}})$ represents the lcevel broadening. The self-energy ${\sum }_{L/R}^{{k}_{//}}$ represents the impact of the right/left electrodes on the central scattering channel. The transmission coefficient *T*(*E*) is then determined via averaging over different ${k}_{//}$ in the irreducible Brillouin zone. Single-ξ polarized basis set is used to expand the electron wave function. A real-space mesh cut-off of 75 Hartree and the room temperature at 300 K are set, respectively.

In the virtual source model, the drain current can be modeled as the product of the mobile charge density and the carrier velocity along the channel. Based on the gradual channel approximation at the VS, the channel charge density per area is,

3 $${I}_{D}={{WQ}}_{x0}{v}_{{inj}}{F}_{{sat}}$$

4 $${F}_{{sat}}=\frac{{V}_{{DS}}/{V}_{{DS}{AT}}}{{\left (1+{\left ({V}_{{DS}}/{V}_{{DS}{AT}}\right)}^{\beta }\right)}^{1/\beta }}$$

where *W* is the device width, ${Q}_{x0}$ is the charge density at VS, ${v}_{{inj}}$ is the saturation injection velocity at the top of the barrier, ${F}_{{sat}}$ denotes the empirical transition function and β is the fitting parameter, ${V}_{{DS}{AT}}$ is the saturation voltage. The intrinsic bias is calculated by

5 $${V}_{{GS}}={V}_{G{S}^{{\prime} }}-{R}_{c}{I}_{D}$$

6 $${V}_{{DS}}={V}_{D{S}^{{\prime} }}-2{R}_{c}{I}_{D}$$

where *R*_*c*_ is the source/drain contact resistance, ${V}_{{DS}}({V}_{D{S}^{{\prime} }})$ and ${V}_{{GS}}({V}_{G{S}^{{\prime} }})$ are the intrinsic (extrinsic) drain voltage and the gate voltage. Then we can extract the virtual source injection velocity ${\upsilon }_{{inj}}$ by fitting the *VS model* to the output characteristics of our FETs. The expression for ballisticity is as follows,

7 $${{B}_{{SAT}}=v}_{{inj}}/{v}_{T}$$

where ${v}_{T0}$ denotes the non-degenerate thermal velocity.

## Supplementary information

Supplementary Information

Peer Review file

## Data Availability

The Source Data underlying the figures of this study are available at https://doi.org/10.6084/m9.figshare.33458686. All raw data generated during the current study are available from the corresponding authors upon request.
