# Supplementary material for: Low contact resistance and high current density in ballistic p-type transistors based on oxygen-doped WSe2 monolayers
Source: Nat Commun. 2026 Oct 1;17:11273. doi: 10.1038/s41467-026-78157-w (PMC13720060; doi:10.1038/s41467-026-78157-w)
Supplement: Supplementary file 1 [file 41467_2026_78157_MOESM1_ESM.pdf]

Supplementary Information for

Low contact resistance and high current density in ballistic p-type transistors based on oxygen-doped WSe_2 monolayers

Lei Sun[1,6], Tingting Gao[2,6], Lin Xu[3,6], Wanying Li[4,6], Chuanqi Zhang[5], Zhengyu Zhang[1], Kian Ping Loh[5], Yuhui He[1], Kaustav Banerjee[3], Yang Chai[2,*], Lain-Jong Li[4,*], Xiangshui Miao[1], Xuefei Li[1,*]

[1]Wuhan National High Magnetic Field Center, School of Integrated Circuits, Huazhong University of Science and Technology, Wuhan 430074, China

[2]Department of Applied Physics, The Hong Kong Polytechnic University, Hung Hom, Kowloon, Hong Kong, China

[3]Department of Electrical and Computer Engineering, University of California, Santa Barbara, CA, USA

[4]Department of Materials Science and Engineering, National University of Singapore, Singapore, Singapore

[5]Department of Chemistry, National University of Singapore, Singapore, Singapore

[6]These authors contributed equally: Lei Sun, Tingting Gao, Lin Xu, and Wanying Li

This document includes:

1. Theoretical calculation of the passivation of Se vacancies in monolayer WSe_2
2. Crystal structures and DFT calculations
3. STM characteristics of as-grown and OM-WSe_2
4. Optical image and AFM of monolayer WSe_2 synthesized by CVD on SiO_2/Si
5. PL spectra of monolayer WSe_2 at room temperature
6. Raman and PL spectra of monolayer WSe_2 after transfer
7. Temperature-dependent PL spectra of as-grown WSe_2 and OM-WSe_2
8. PL spectra of monolayer WSe_2 doping with different O_2 concentration at 4 K
9. Laser power-dependent PL spectra of as-grown WSe_2 and OM-WSe_2
10. XPS spectra of WSe_2 before and after doping
11. Dielectric leakage of WSe_2 with after growth and after the post-annealing process
12. The C-V curves of purchased SiO_2/Si substrates without and with post-annealing
13. Mobility statistics of monolayer WSe_2 pFETs doping with different O_2 concentration
14. Output characteristics of WSe_2 transistor without O_2 doping
15. Hysteresis of as-grown WSe_2 and OM-WSe_2 transistors
16. Output characteristics of monolayer WSe_2 transistors without and with Ar annealing
17. Schematic diagram of the device fabrication process
18. Transfer characteristics curve statistics data of devices with doping first and doping last
19. Evidence of oxygen atoms diffusing underneath the metal contacts to passivate defects in the contact regions

20. Transfer curves of WSe_2 TLM devices with doping first and with doping last
21. Statistical analysis on the contact resistance of the same batch of devices
22. Extraction of the effective Schottky barrier height (SBH) of monolayer WSe_2 transistors with doping first and with doping last
23. Output characteristics of monolayer WSe_2 transistors using full contact at 4 K
24. TEM and EDS mapping of the ballistic monolayer WSe_2 transistor
25. Transfer characteristics of WSe_2 transistors without and with O_2 doping on HfO_2 gate dielectrics
26. Extraction of the external interconnection metal line resistance
27. Benchmarking on-state resistance of WSe_2 transistors
28. Stability of OM-WSe_2 transistors
29. Temperature-dependent transfer characteristics and mobility of the monolayer WSe_2 transistor with doping last
30. Temperature-dependent carrier injection velocity and channel-length dependence of current in short-channel devices.
31. Benchmarking on-state current of WSe_2 transistors

Table 1 Benchmarking state-of-art WSe_2 p-type transistors

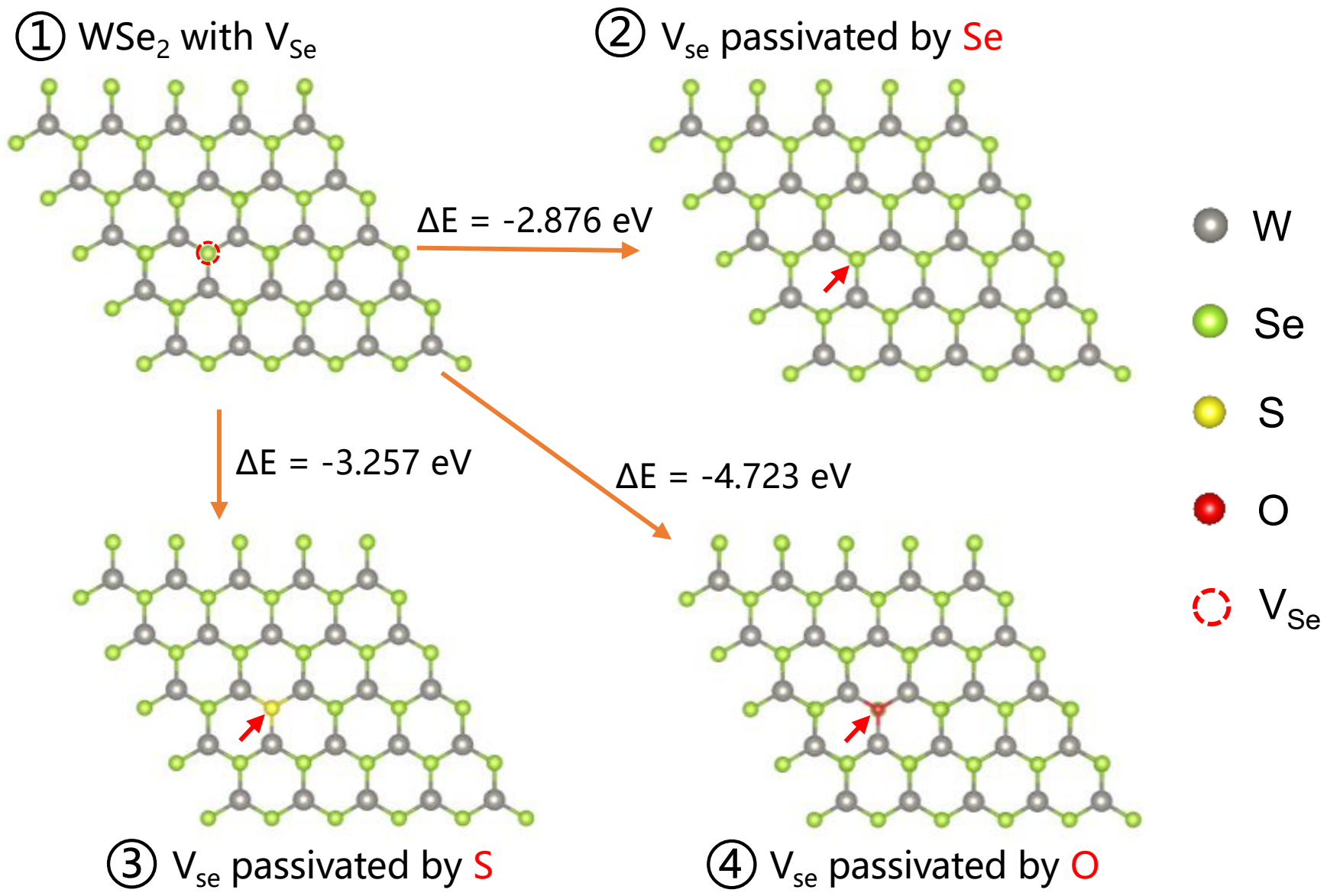

Supplementary Fig. 1. Theoretical calculation of using different atoms to passivate Se vacancies in monolayer WSe_2. It shows the formation energy (ΔE) for passivating V_{Se} via Se, S, and O, and the result indicates the ΔE of O-passivated V_{Se} is the lowest.

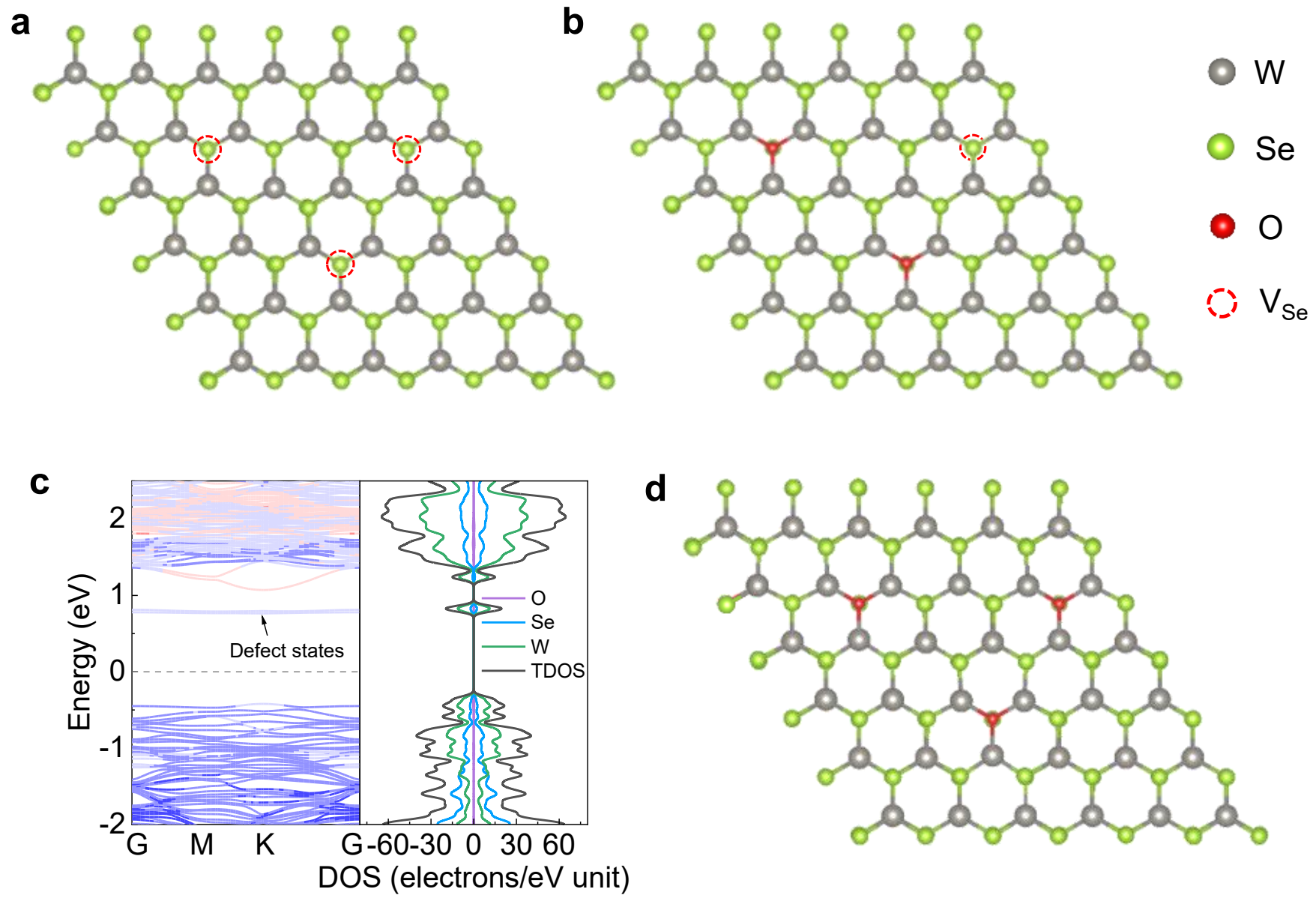

Supplementary Fig. 2. Crystal structures and DFT calculations. (a) Crystal structure of a 5×5 supercell with 3 V_{Se} corresponding to Fig. 1d in the main text. **(b)**

Crystal structure of a 5×5 supercell with 2 O-passivated V_{Se} and 1 V_{Se} corresponding to (c). **(c)** Band and DOS of monolayer WSe_2 which still has a V_{Se} density of 1.4×10^{13} cm^{-2} after O passivation, and the defect states are significantly less than those of as-grown WSe_2 as shown in Fig. 1d in the main text. **(d)** Crystal structure of a 5×5 supercell with 3 O-passivated V_{Se} corresponding to Fig. 1e in the main text.

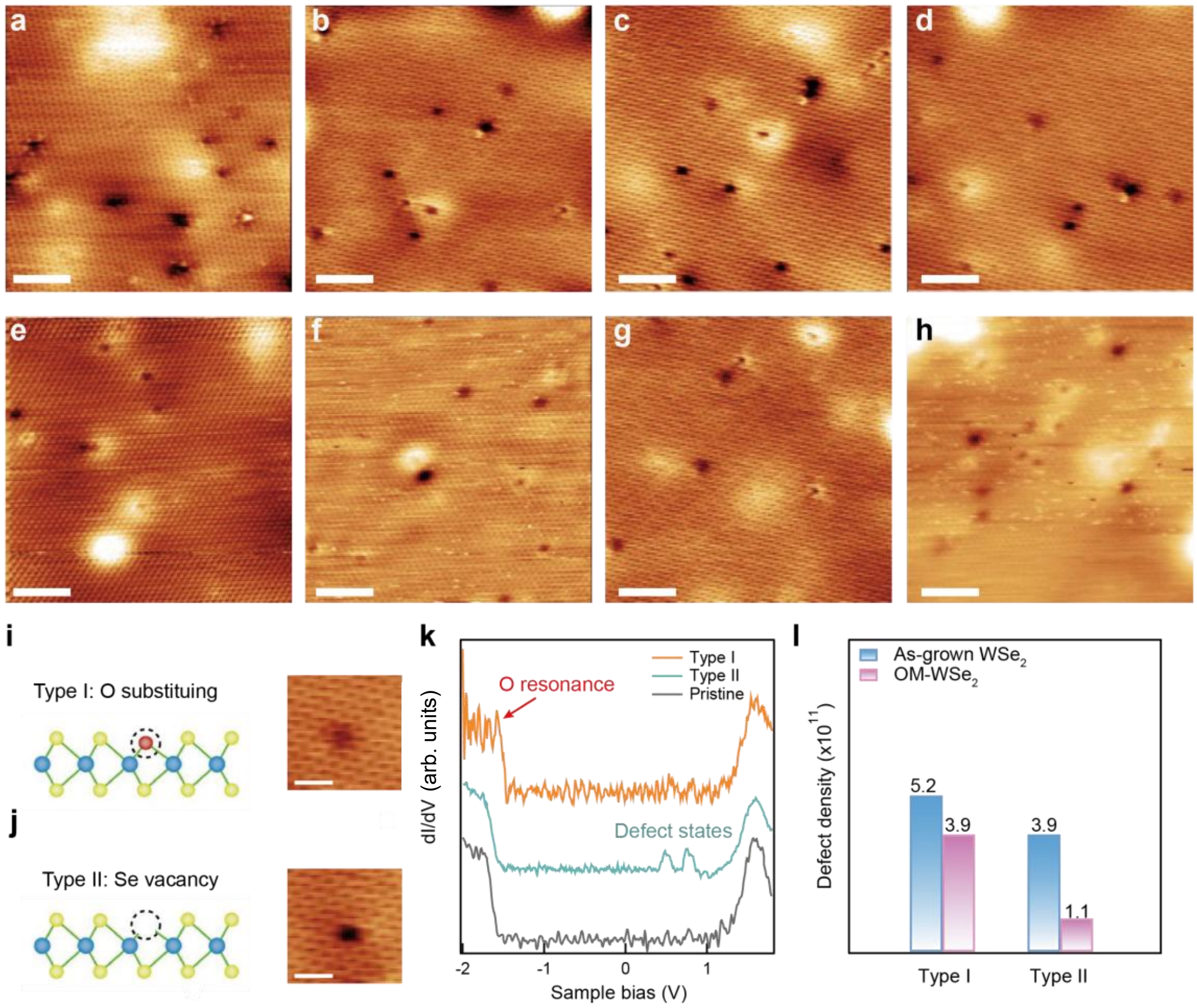

Supplementary Fig. 3. STM characteristics of as-grown and OM-WSe_2. STM topography images of the monolayer as-grown WSe_2 **(a-d)** and OM-WSe_2 **(e-h)**, scale bar:3 nm. **(i-j)** Schematic and STM images of type I and type II defect which represent

oxygen atom substituting Se and Se atom vacancy, respectively. scale bar: 1 nm. **(k)** STS spectra acquired on the corresponding defect types. An additional defect resonance as arrow marked is observed in the defect's spectra indicating substitutional oxygen in type I spectra[1]. The defect states in type II spectra in bandgap consistent with our DFT results. The spectra of pristine WSe_2 in dark gray color represent perfect lattice structure. **(l)** Comparison of type I and type II defect density in as-grown and OM-WSe_2.

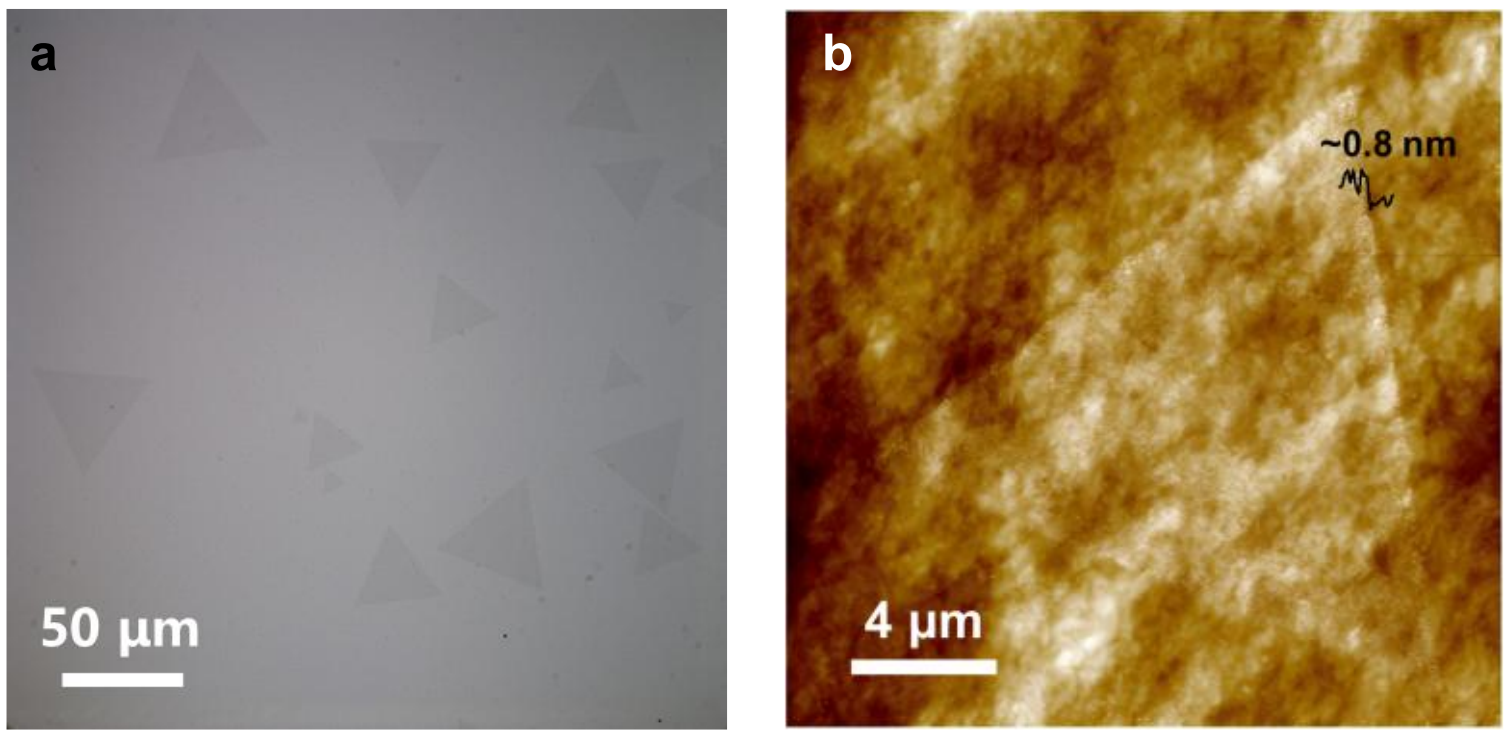

Supplementary Fig. 4. Characterization of CVD monolayer WSe_2. (a) Optical image of monolayer WSe_2 synthesized by CVD on 15 nm SiO_2/Si substrates. **(b)** AFM of monolayer WSe_2 synthesized by CVD on 100 nm SiO_2/Si substrates. The height profiles show that the thickness of monolayer WSe_2 is ~ 0.8 nm.

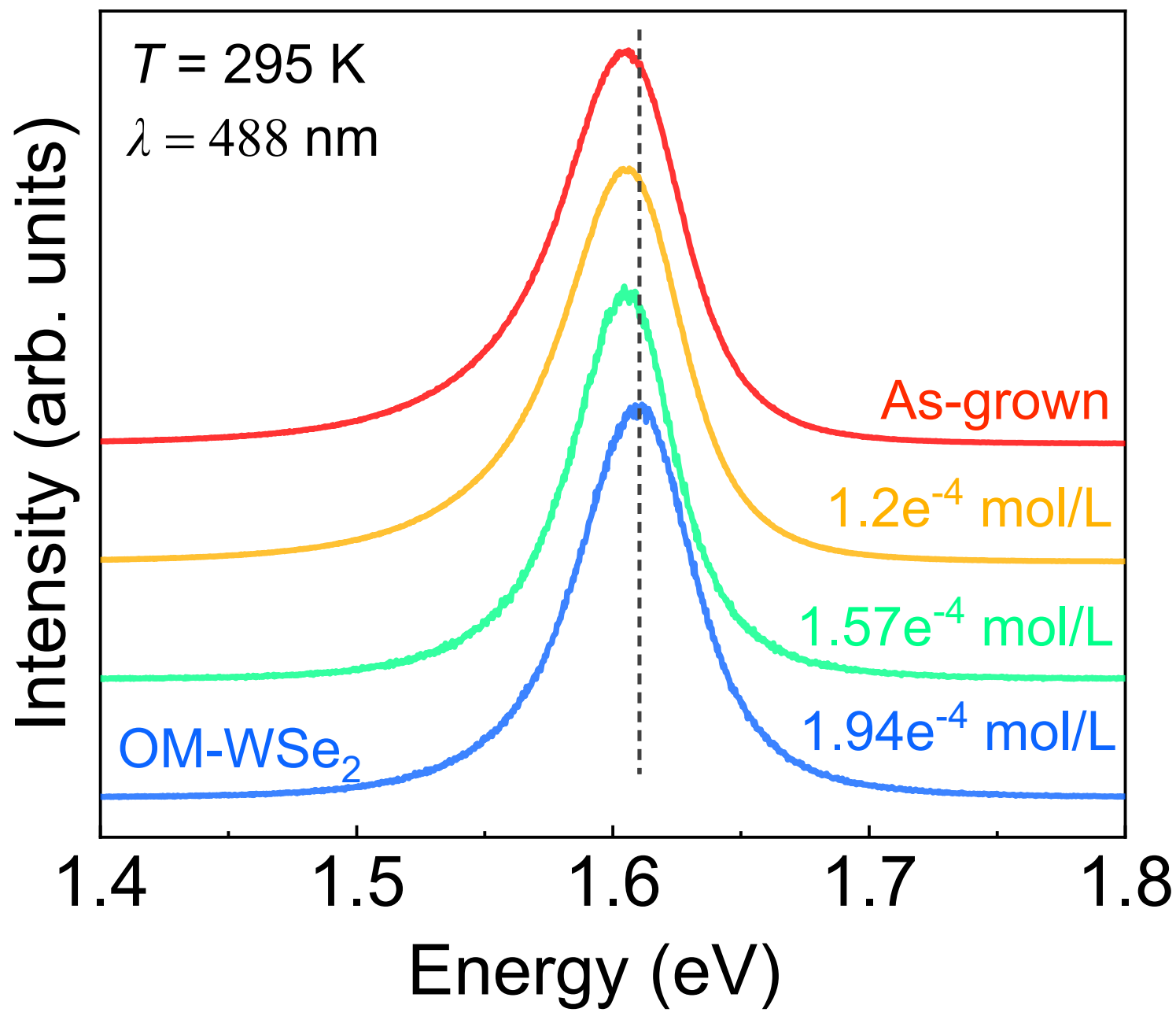

Supplementary Fig. 5. PL spectra of monolayer WSe_2 on 100 nm SiO_2/Si substrates at room temperature. It shows the full width at half maximum (FWHM) of WSe_2 without and with different concentrations O_2 doping have a trend of initially decreasing, indicating the quality of WSe_2 crystal improved.

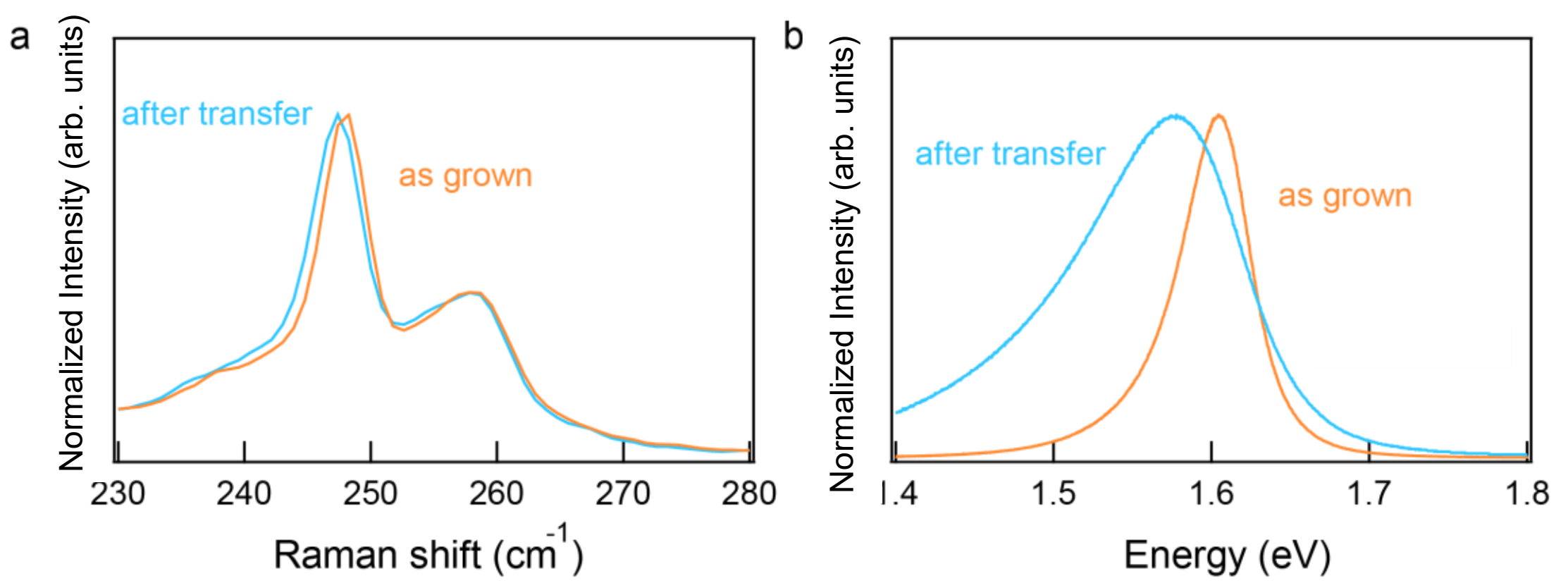

Supplementary Fig. 6. Raman and PL spectra of monolayer WSe_2 after transfer. Raman **(a)** and PL **(b)** spectra of as grown (orange curve) WSe_2 and after transfer to

another silicon substrate (blue curve). After transfer from as-grown SiO_2 to another SiO_2 substrate, both Raman and PL red shift, which indicates that the as-grown WSe_2 exhibit some p-doping effect likely induced during the growth that is weakened after transfer. Based on the Raman and PL shift, it is unlikely attributed to the strain effect[2,3].

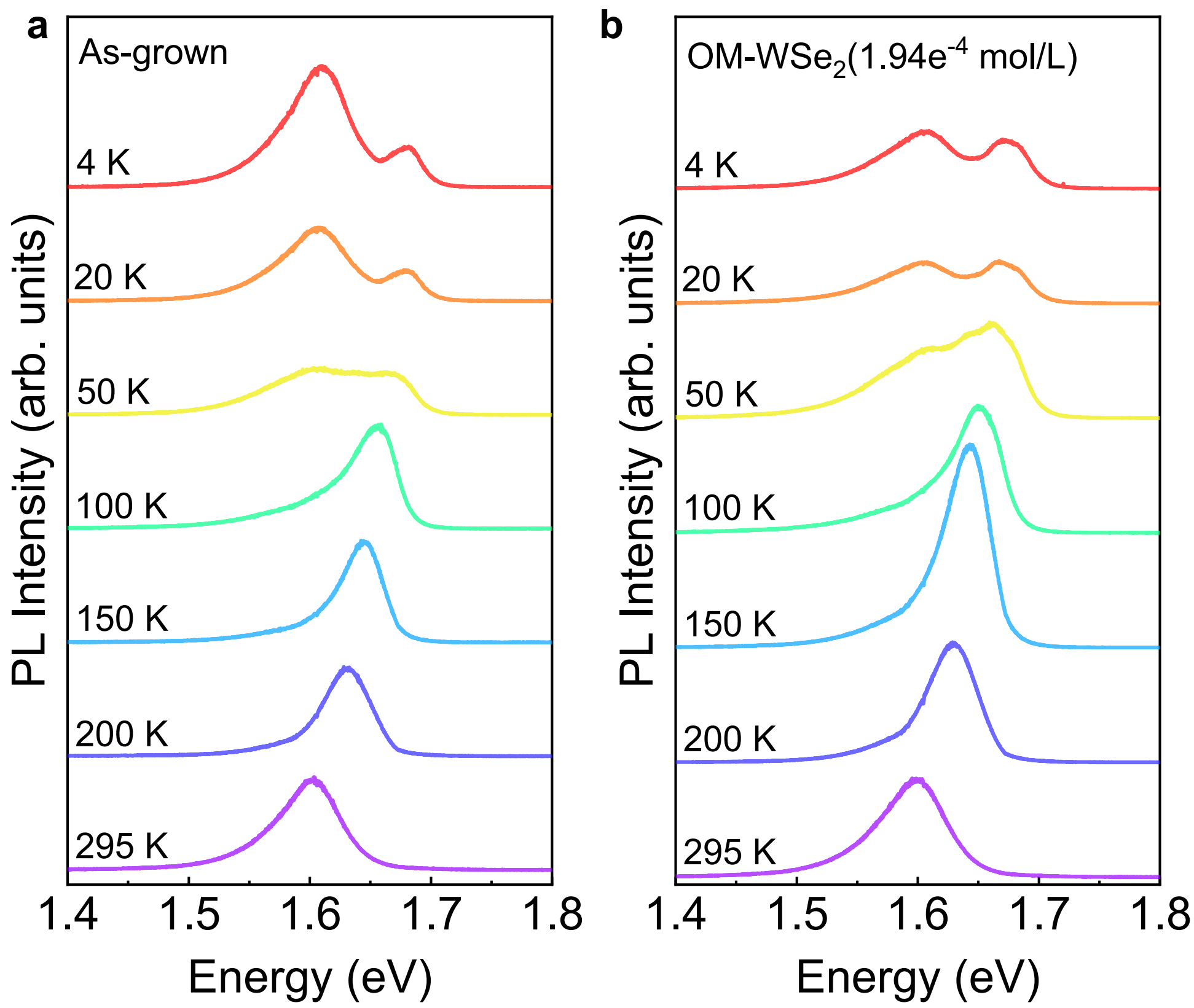

Supplementary Fig. 7. Temperature-dependent PL spectra of monolayer WSe_2 on 100 nm SiO_2/Si substrates. PL spectra of monolayer WSe_2 without **(a)** and with **(b)** $1.94e^{-4}$ mol/L O_2 doping. The defect-bound exciton peak decreases while the free exciton peak increases with increasing temperature from 4 K to 295 K due to thermal fluctuations[4].

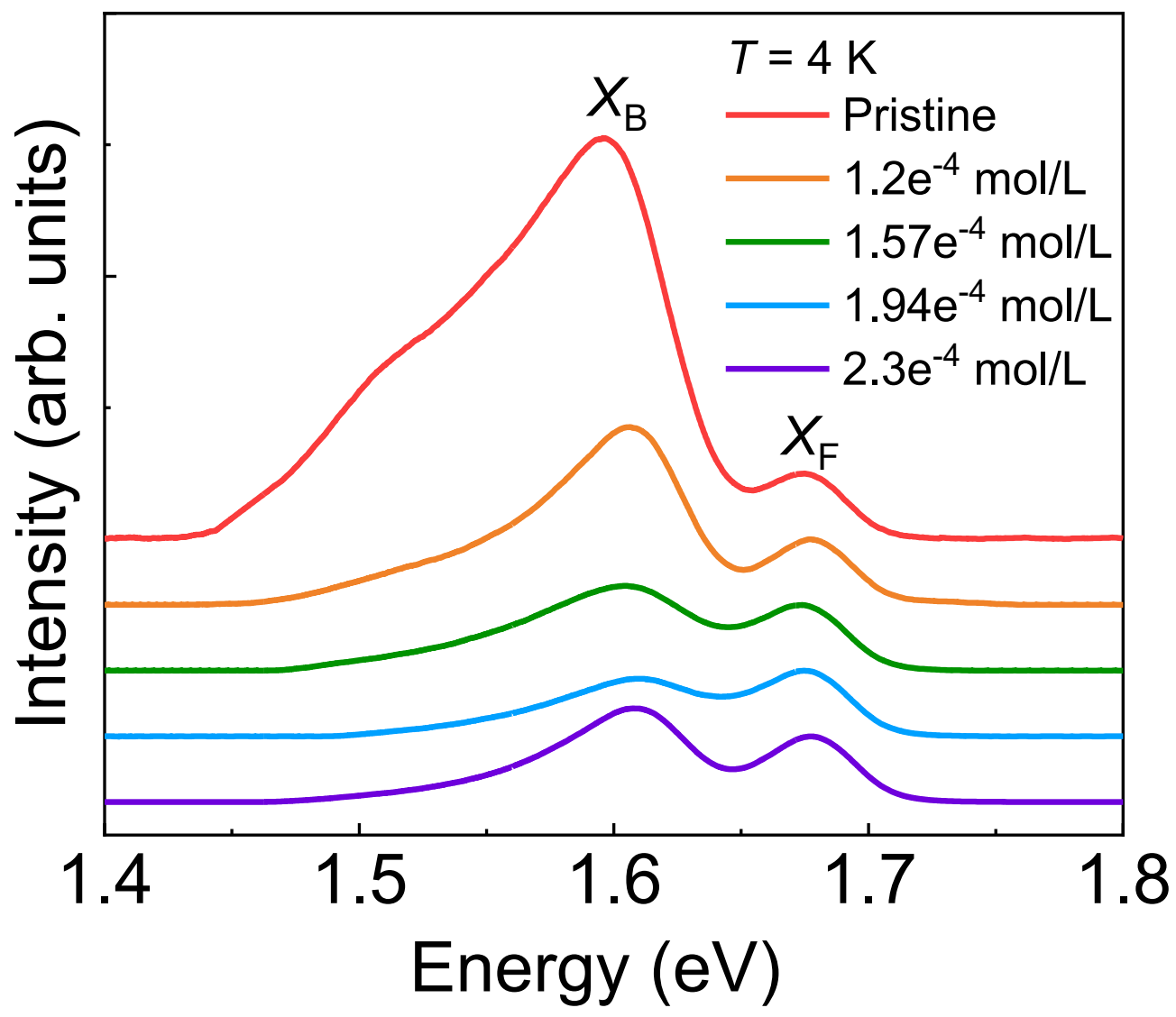

Supplementary Fig. 8. PL spectra of monolayer WSe_2 doping with different O_2 concentration at 4 K. The intensity of the bound exciton peak decreased with increasing O_2 concentration. With the oxygen concentration increases further, the intensity of the bound exciton peak increased and defects increased.

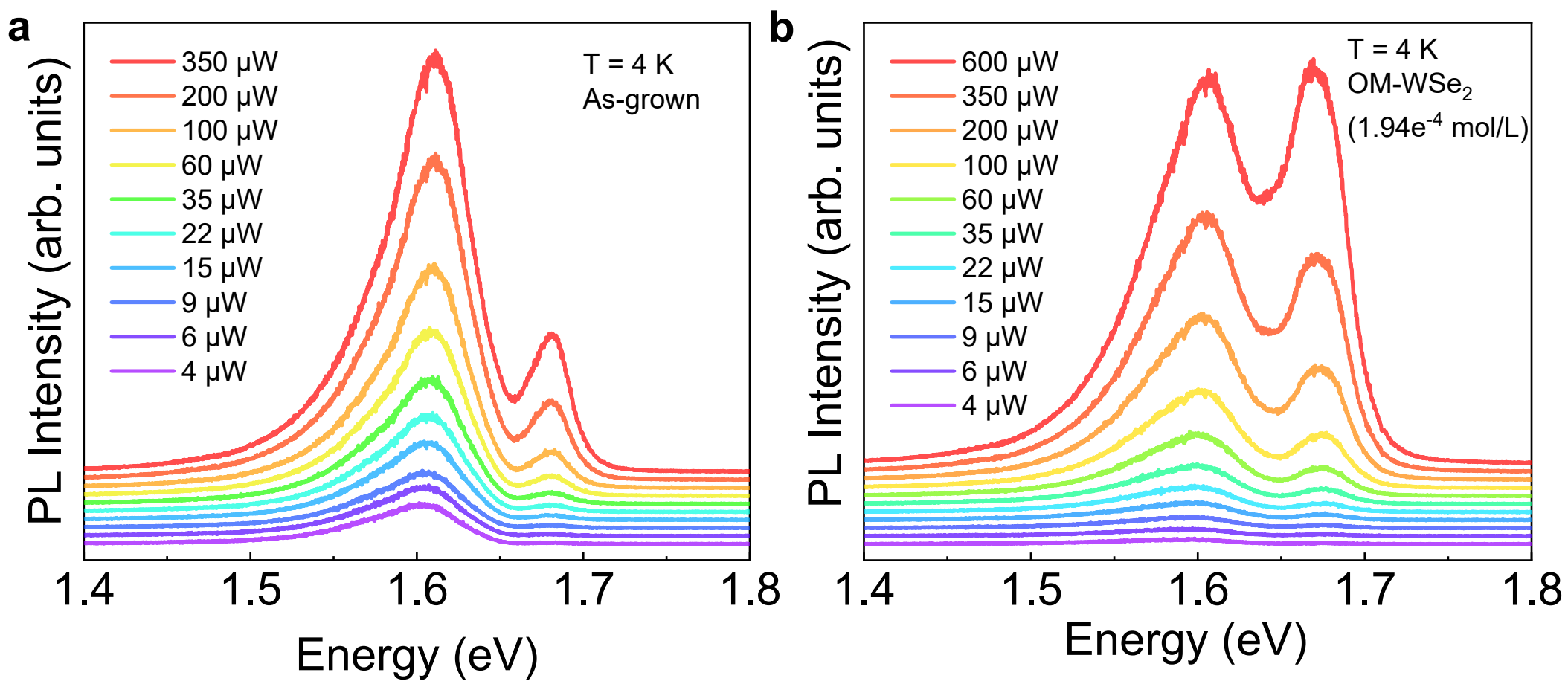

Supplementary Fig. 9. Low-temperature PL spectra of monolayer WSe_2 without or with O_2 doping. Laser power-dependent PL spectra of monolayer WSe_2 without **(a)** and with **(b)** $1.94e^{-4}$ mol/L O_2 doping.

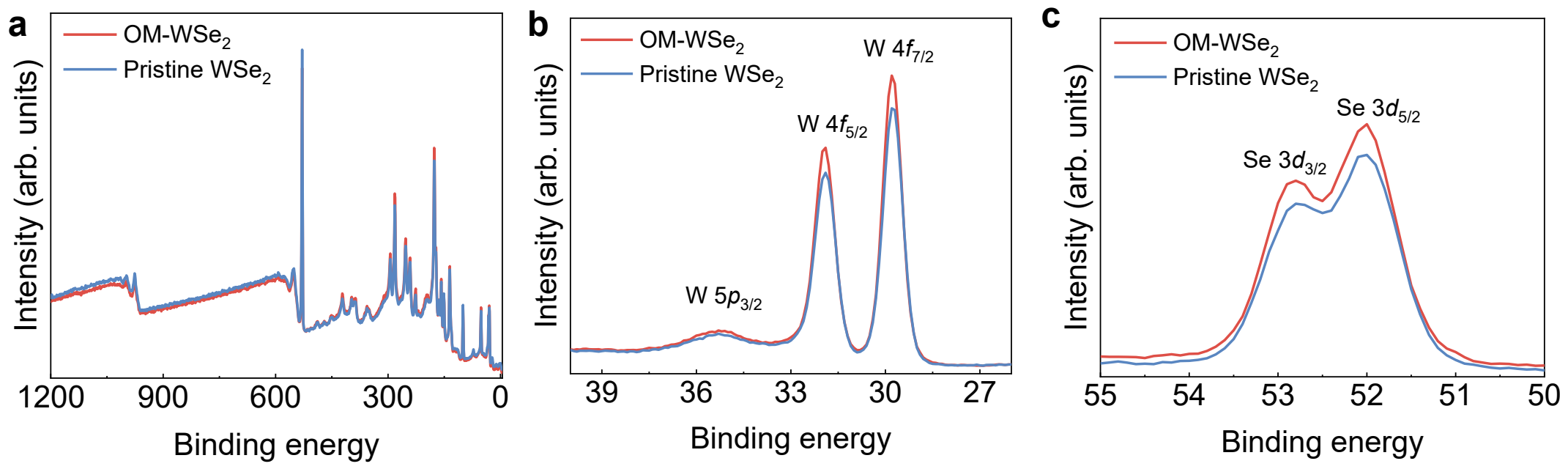

Supplementary Fig. 10. XPS spectra of WSe_2 before and after doping: (a) Survey spectrum, (b) W 4*f*, (c) Se 3*d*. The change of chemical composition of the samples is not obvious before and after doping.

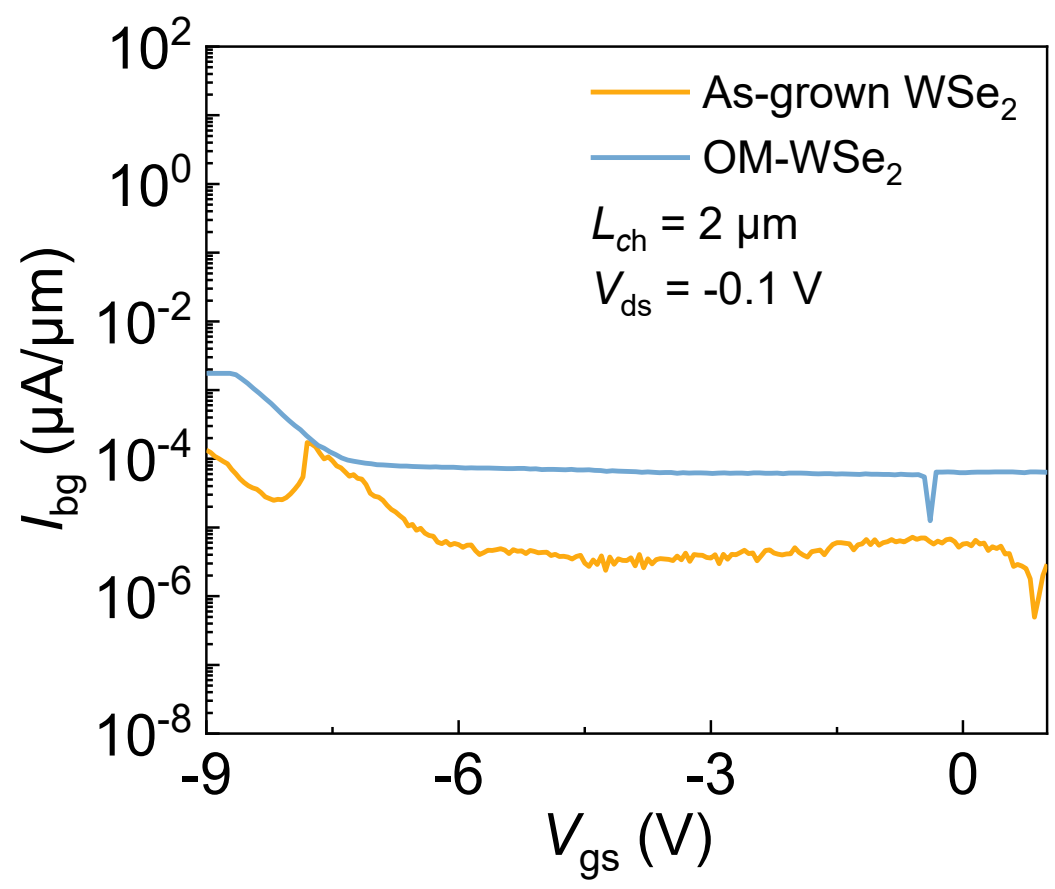

Supplementary Fig. 11. Gate leakage currents both after growth and after the post-annealing process, corresponding to Fig. 2f in main text. The gate dielectric is 15-nm SiO_2. The gate leakage current of both devices is always below 2 nA, indicating the excellent insulator properties of SiO_2.

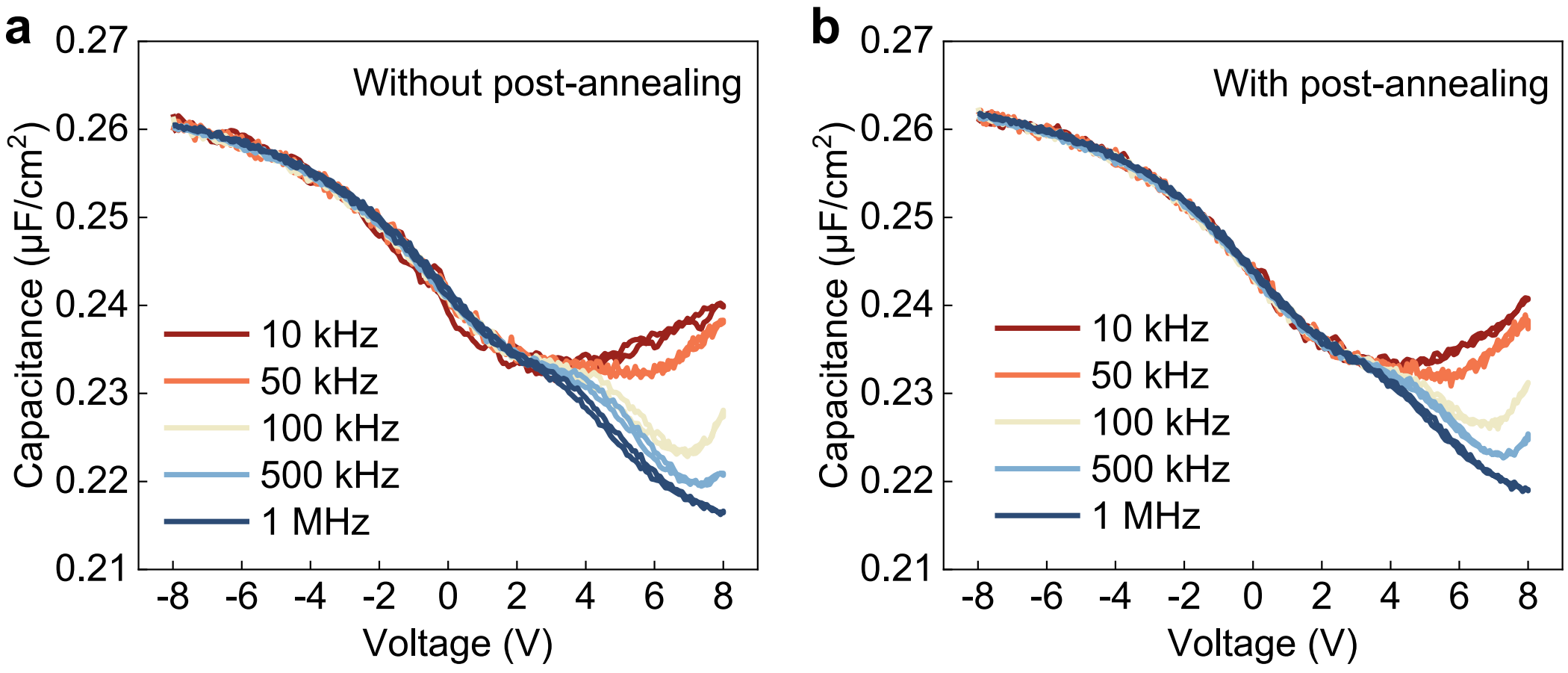

Supplementary Fig. 12. The C-V curves of SiO_2/Si substrates without (a) and with (b) post-annealing. The areal capacitance of the gate dielectric (Cox is 0.26 μF/cm^2. Since the variation in C_{ox} before and after annealing is negligible, a constant C_{ox} value is adopted for all mobility extractions to ensure a fair comparison.

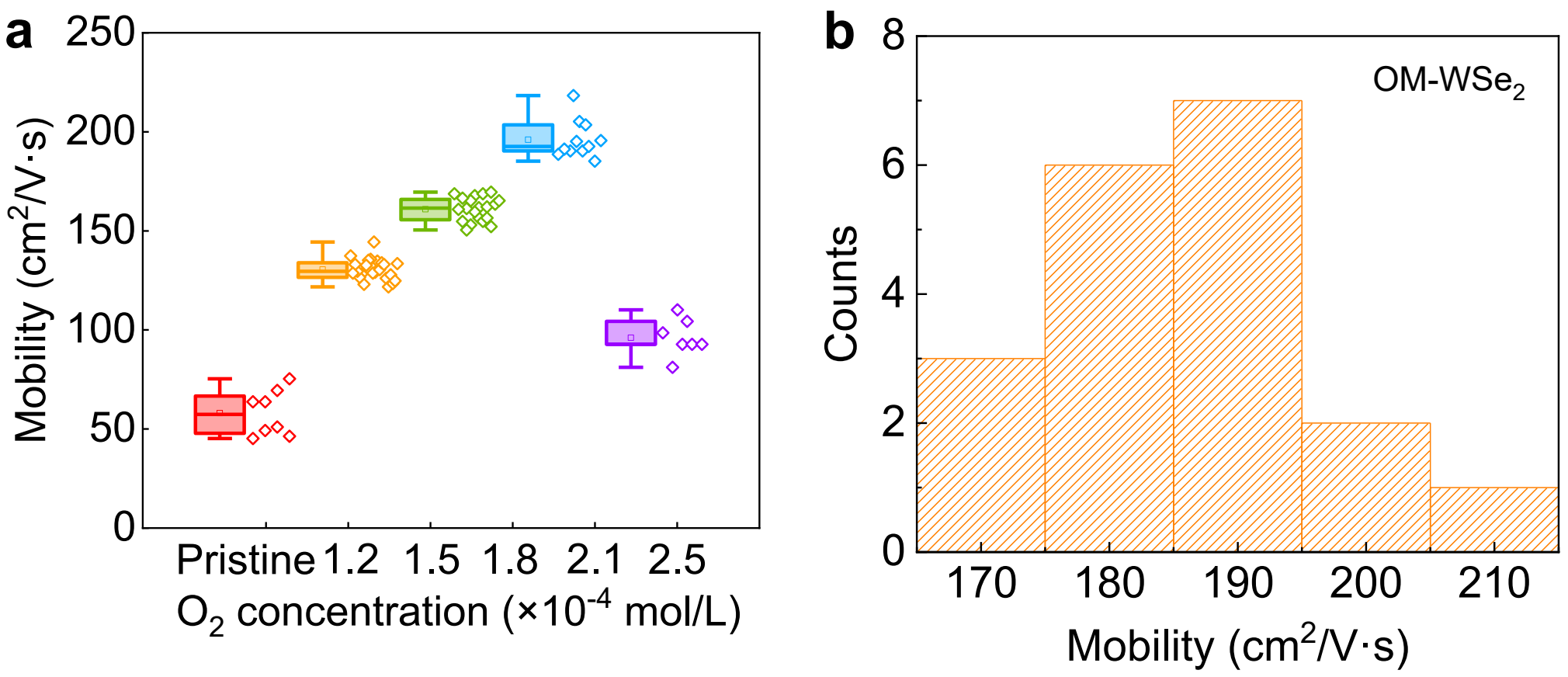

Supplementary Fig. 13. Mobility statistics of monolayer WSe_2 pFETs doping with different O_2 concentration. (a) The transistor mobility increased to theoretical limit with increasing O_2 treatment concentration[5]. With the oxygen concentration increases further, the mobility of transistors decreased. (**b**) Statistics of mobility for 19 devices with long channel. The mobility and contact resistance of OM-WSe_2 transistors are 189.2 ± 11.2 cm^2/V·s.

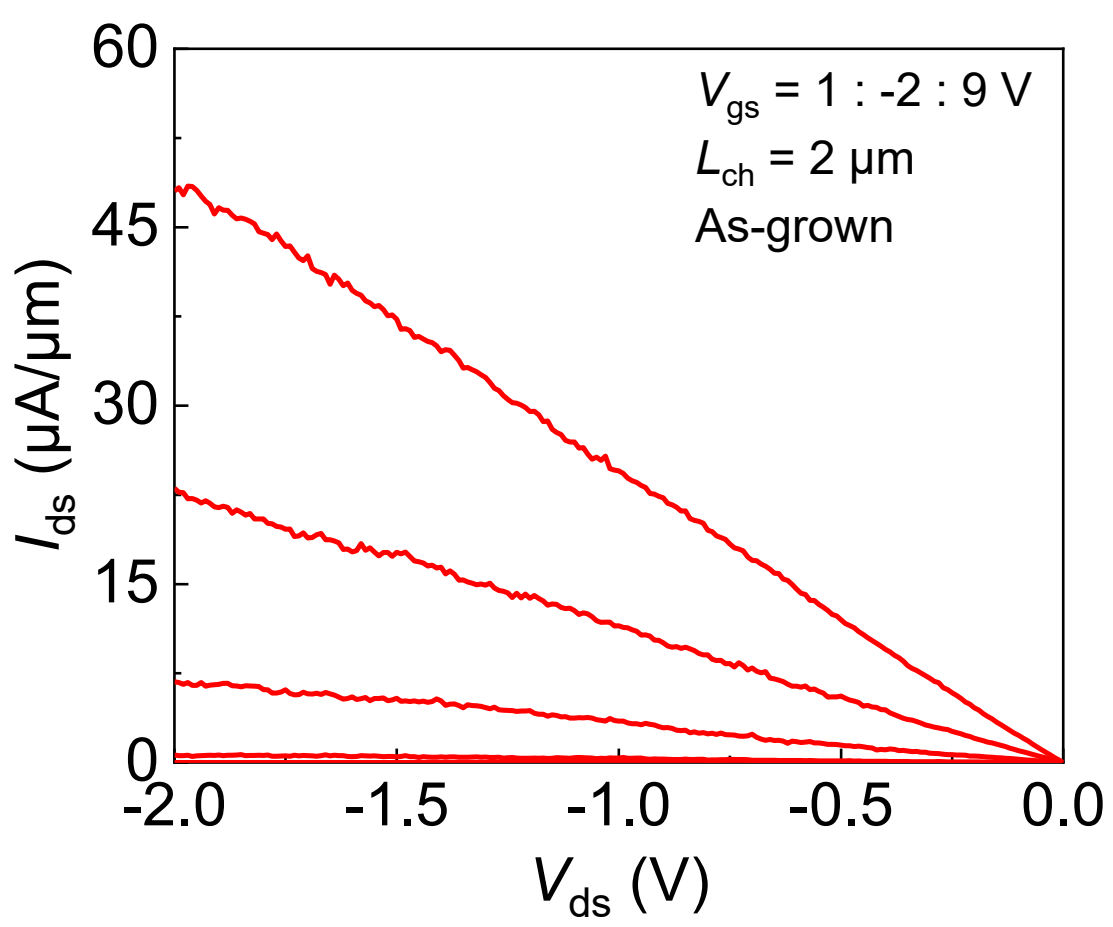

Supplementary Fig. 14. Output characteristics of WSe_2 transistor without O_2 doping. The output current density of the as-grown WSe_2 transistor is low.

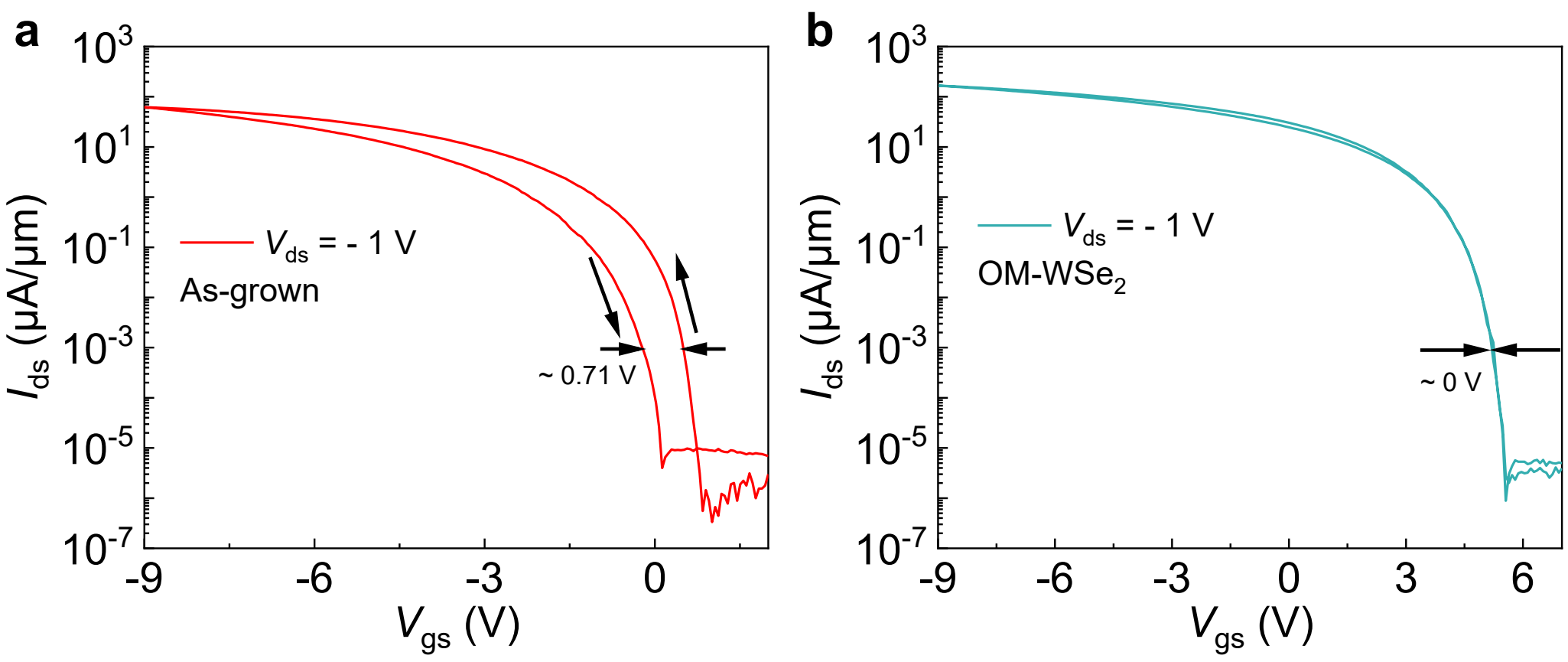

Supplementary Fig. 15. Hysteresis of monolayer WSe_2 transistors without and with appropriate O_2 doping. Transfer characteristics of the transistor before **(a)** and after **(b)** appropriate O_2 doping[6]. After O_2 doping, the hysteresis decreased from ~0.71 V to ~0 V.

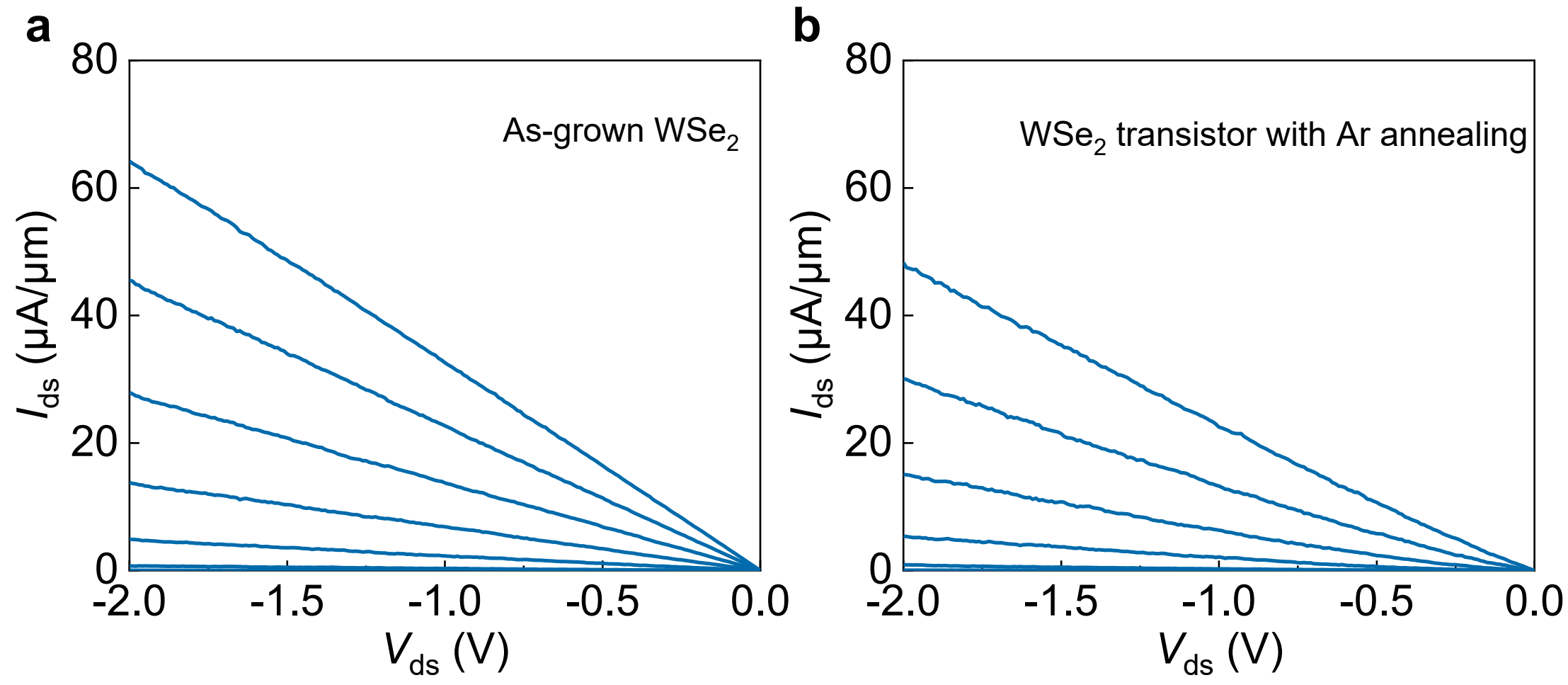

Supplementary Fig. 16. Output characteristics of monolayer WSe_2 transistors without (a) and with (b) Ar annealing. After Ar annealing, the on-state current of the transistor decreases slightly.

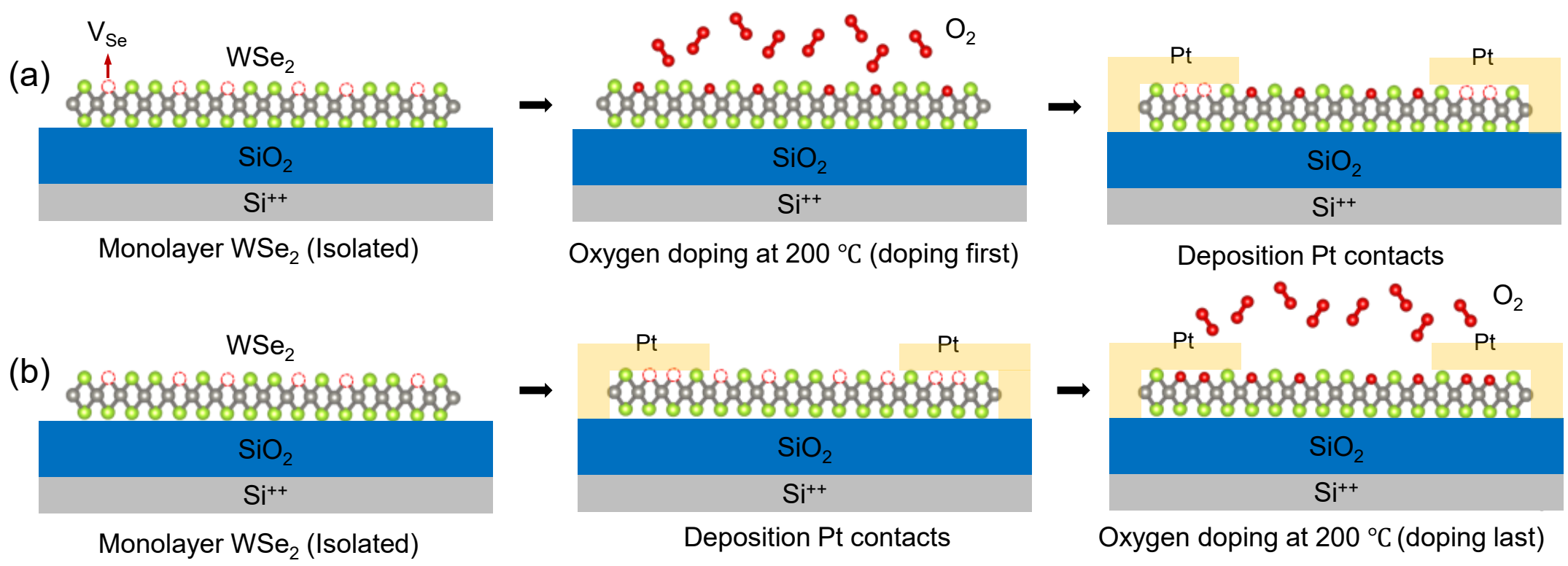

Supplementary Fig. 17. Schematic diagram of the device fabrication process. Fabrication process of monolayer WSe_2 transistors **(a)** with doping first (annealing before S/D contacts) and **(b)** with doping last (annealing after S/D contacts).

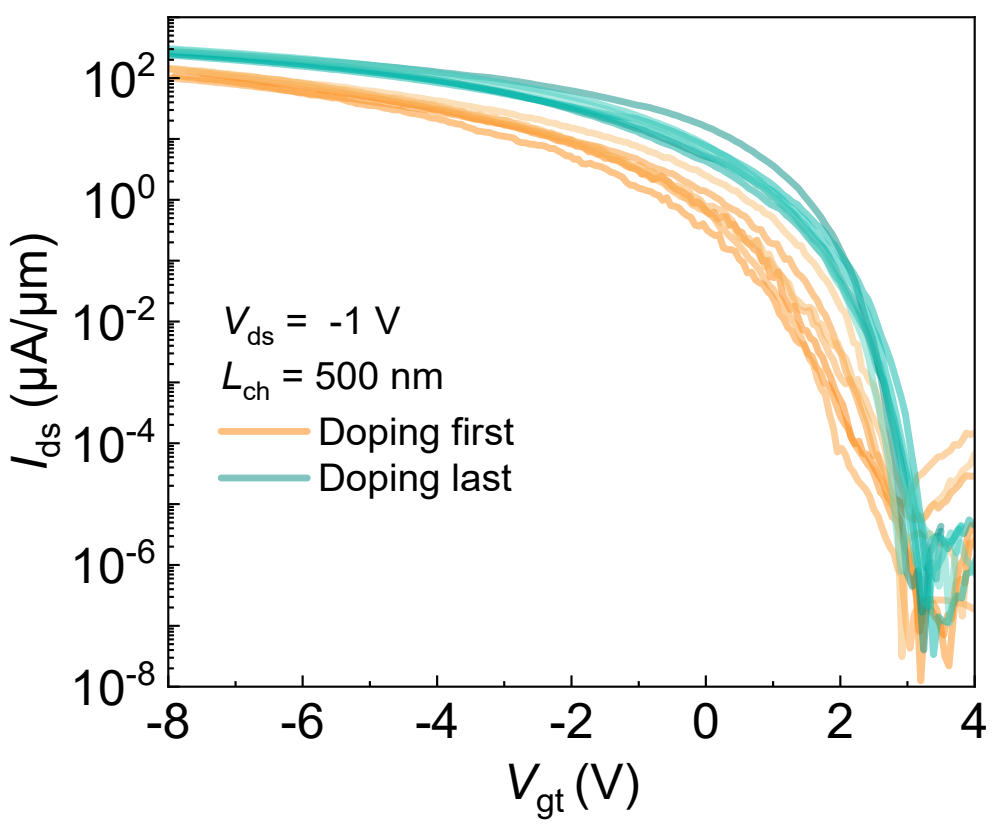

Supplementary Fig. 18. Transfer characteristics curve statistics data of devices with doping first and doping last. The device-to-device variation is comparable between the two groups (similar standard deviations), ruling out the possibility that the lack of visible difference is merely due to random device fluctuations.

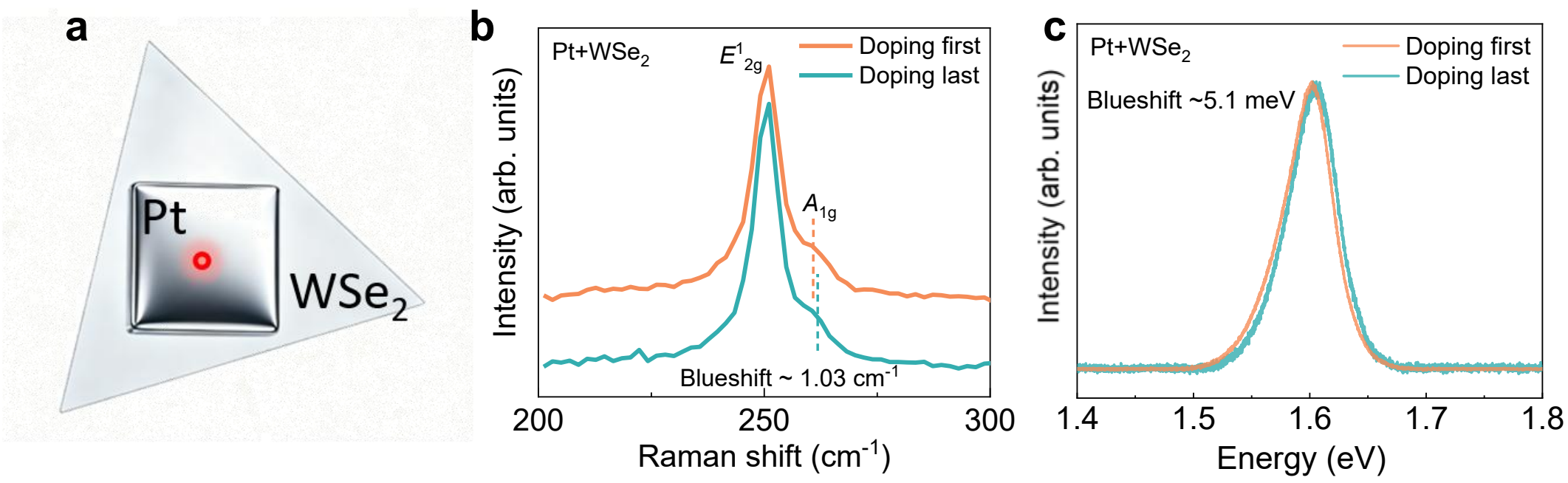

Supplementary Fig. 19. (**a**) Schematic diagram of Raman measurement for 3-nm Pt+1L WSe_2. Raman (**b**) and PL (**c**) Spectra of Pt+WSe_2 with doping first and doping last. The blueshift of the Raman A_{1g} peak and the PL peak for doping last confirmed the p-type doping, which prove that oxygen atoms diffused underneath the metal contacts and repaired defects at the buried interface.

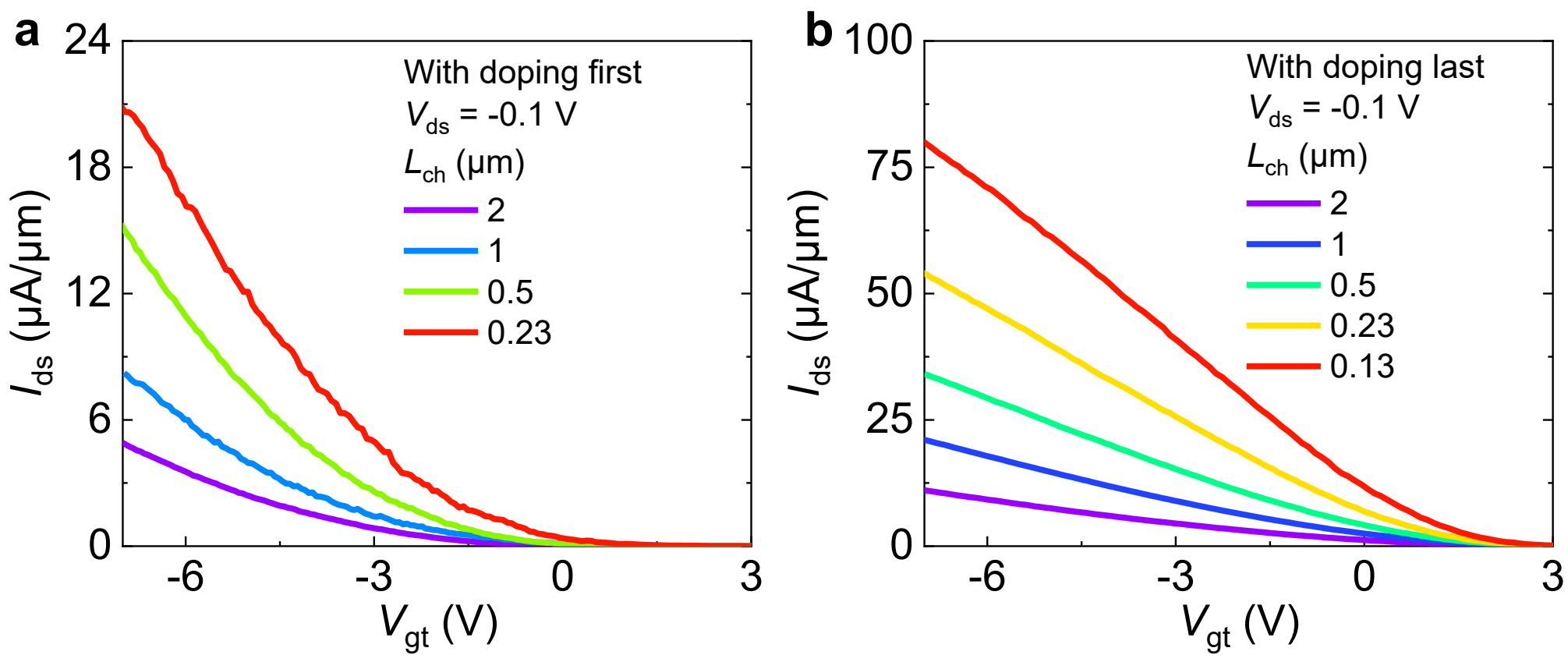

Supplementary Fig. 20. Transfer curves of WSe_2 TLM devices with doping first (a) and with doping last (b). The performance of transistors with doping last is significantly better than that with doping first. This is attributed to that the damage to WSe_2 in the contact area during metal deposition process is also passivated by O_2 doping.

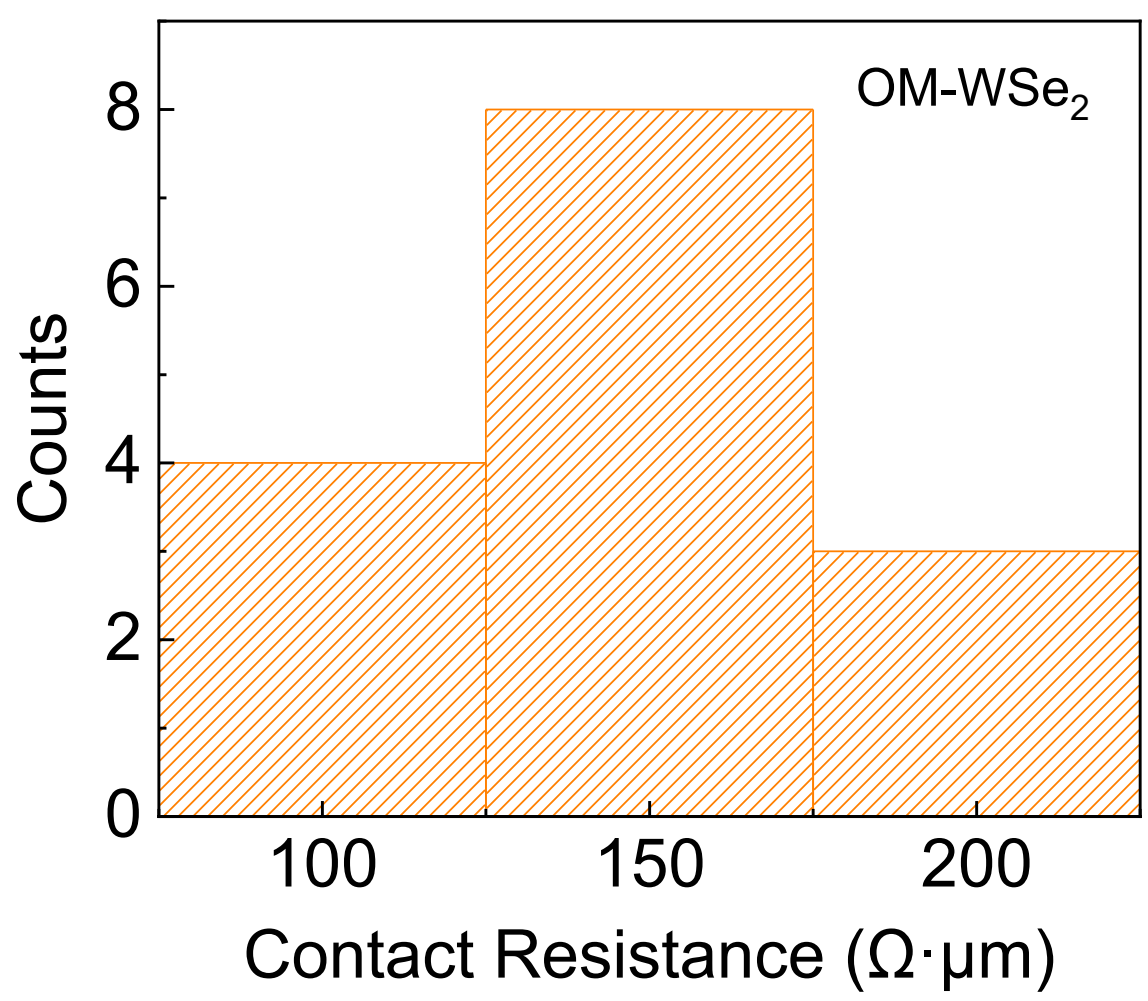

Supplementary Fig. 21. Statistics of contact resistance for 15 TLM structures. The contact resistance of OM-WSe_2 transistors is 165 ± 39 Ω·μm.

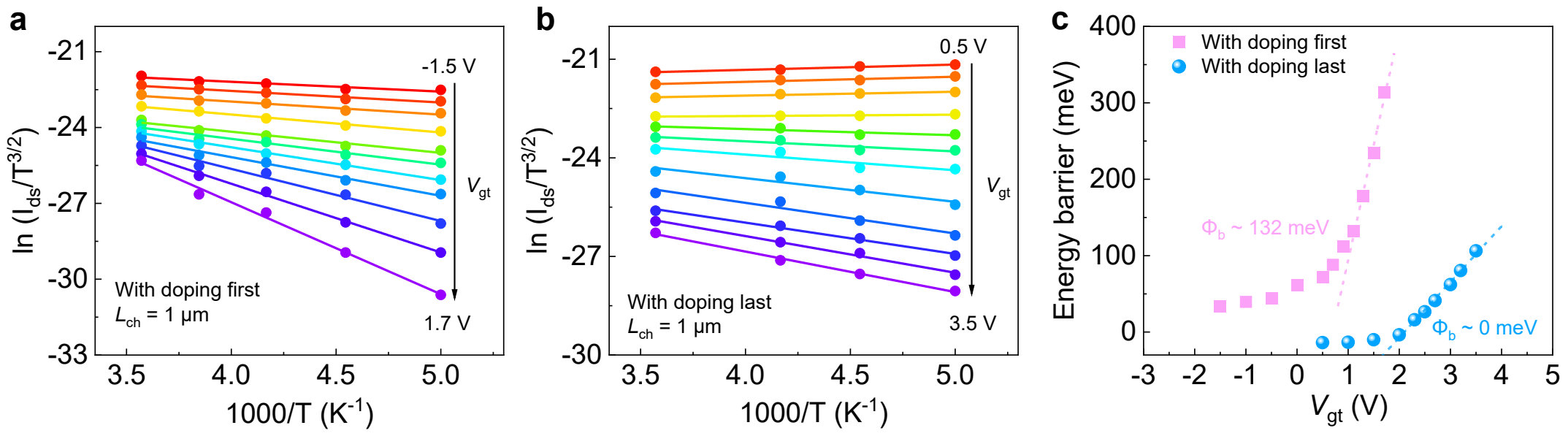

Supplementary Fig. 22. Extraction of the effective Schottky barrier height (SBH) of monolayer WSe_2 transistors with doping first and with doping last. Arrhenius plots of monolayer WSe_2 transistors with doping first **(a)** and with doping last **(b)**. **(c)** Effective contact barriers at various gate biases corresponding to **(a)** and **(b)**. Schottky barrier heights (SBHs) are extracted under the flat-band conditions. The SBHs with doping first and with doping last are ~132 meV and ~0 meV, respectively.

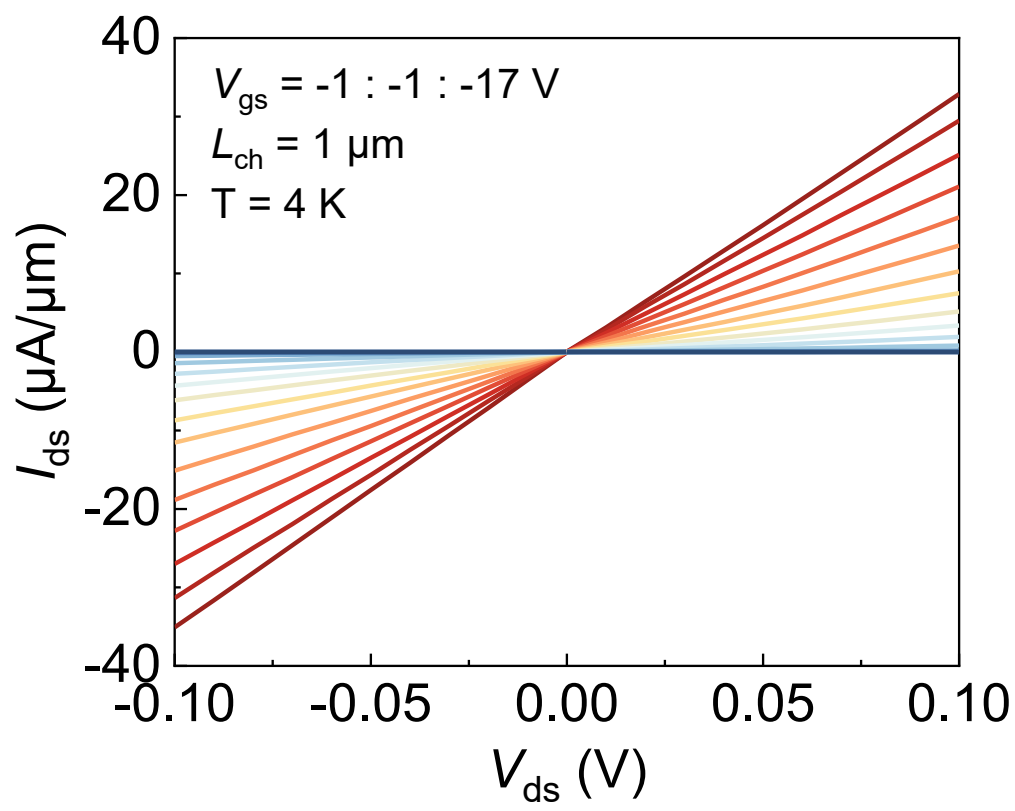

Supplementary Fig. 23. Output characteristics of monolayer WSe_2 transistors with doping last at 4 K. The linear output characteristics of transistors at 4 K show that the contact is ohmic[7].

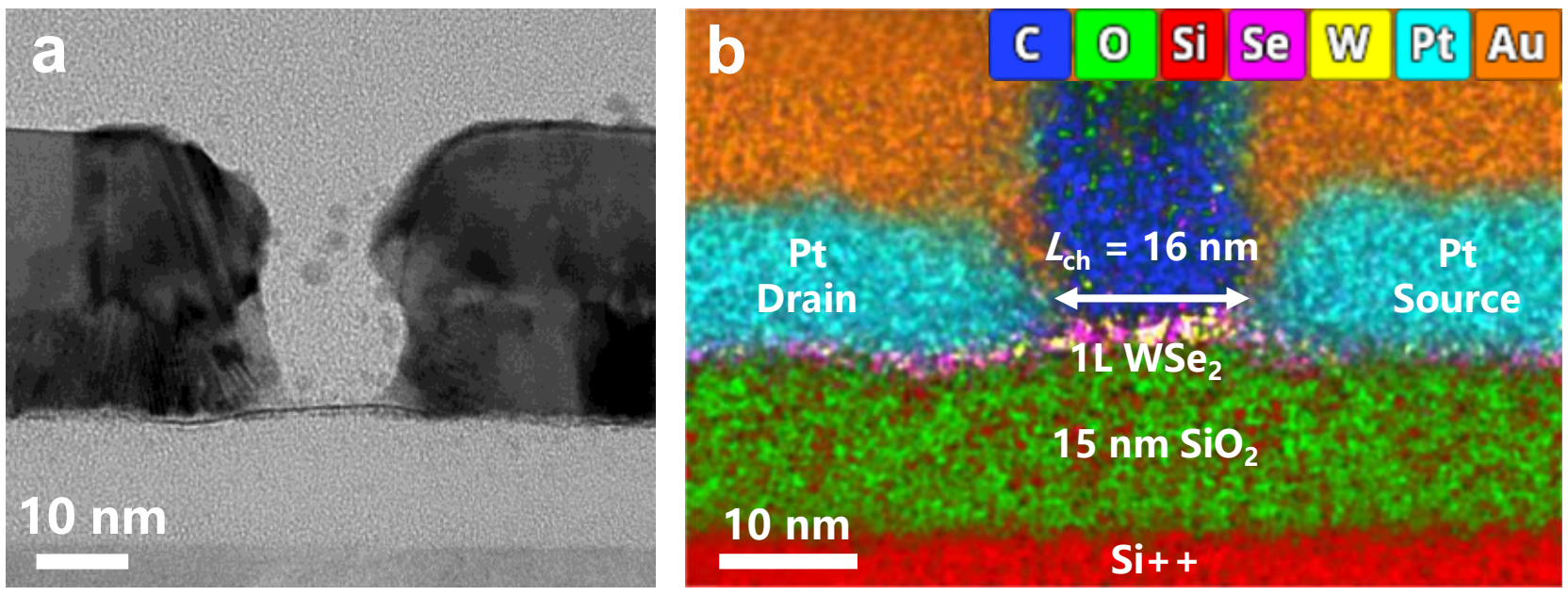

Supplementary Fig. 24. TEM (a) and EDS mapping (b) of the ballistic monolayer WSe_2 transistor. The channel length of the device is approximately 16 nm.

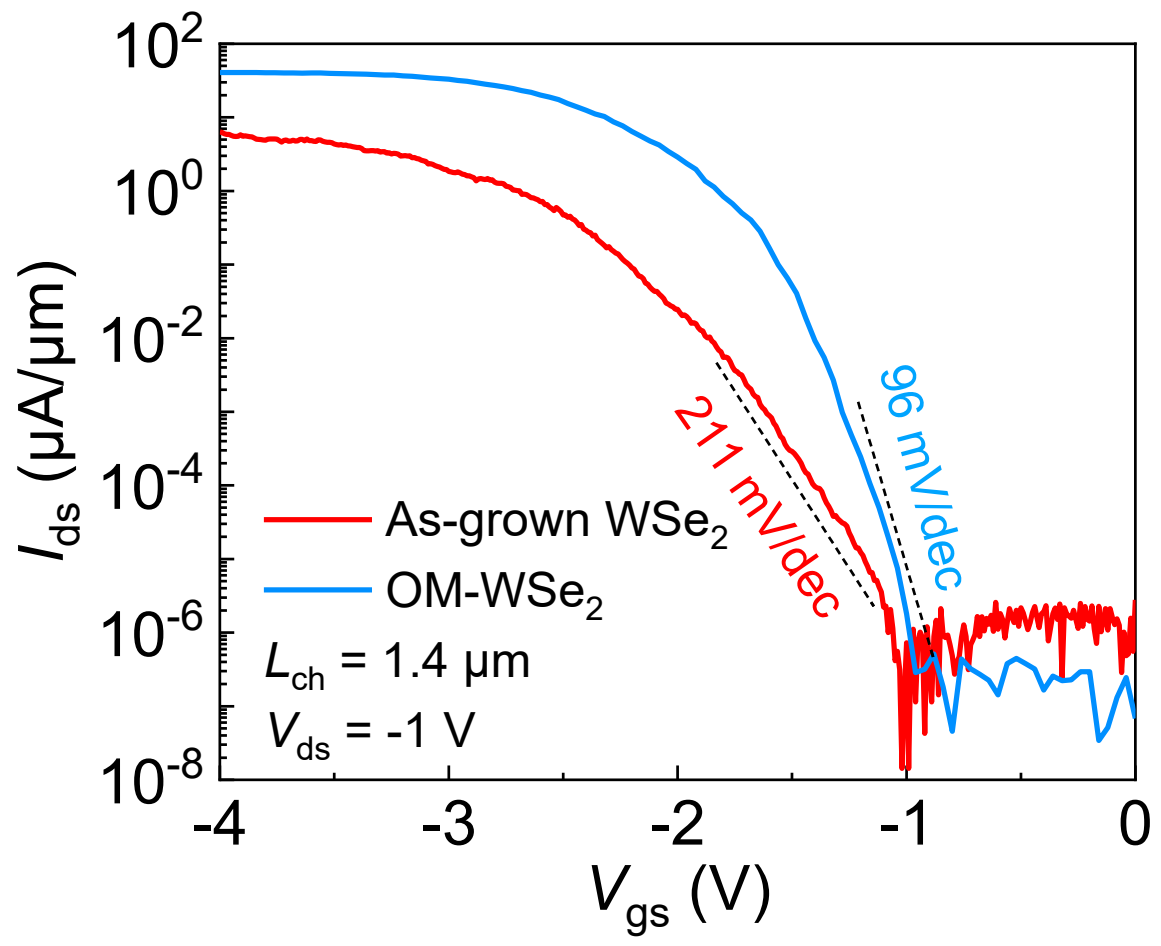

Supplementary Fig. 25. Transfer characteristics of WSe_2 transistors without and with O_2 doping on HfO_2 gate dielectrics. The on-current of the monolayer transistor significantly improved, and the SS decreased from 211 mV/dec to 96 mV/dec after O_2 doping. The mobility of device before and after doping was 5.3 and 13 $cm^2/V{\cdot}s$, respectively. The reduction in SS after annealing are direct evidence of interface state passivation. Oxygen annealing effectively supplies oxygen atoms to diffuse to the HfO_2/Si interface, terminating the dangling bonds and reducing the D_{it} from ~4.45×10^{13} $eV^{-1}cm^{-2}$ to ~1.06×10^{13} $eV^{-1}cm^{-2}$ (calculated via $SS=\frac{k_BT}{q}ln(10)(1+\frac{qD_{it}}{C_{ox}})$). This passivation reduces the trap-assisted recombination, thereby steepening the SS. Besides

interface passivation, oxygen annealing plays a crucial role in suppressing remote Coulomb scattering. The as-deposited HfO_2 films typically contain a high density of oxygen vacancies (V_O) acting as charged bulk traps. These bulk traps are located physically close to the channel; their long-range Coulomb potential scatters the channel carriers, degrading the subthreshold slope (especially at low drain current levels). After oxygen annealing, these oxygen vacancies are effectively filled, reducing the fixed bulk charge density. The reduction of these remote charged centers weakens the Coulomb scattering potential in the channel, which is corroborated by the improved carrier mobility and the steeper SS observed in the linear region.

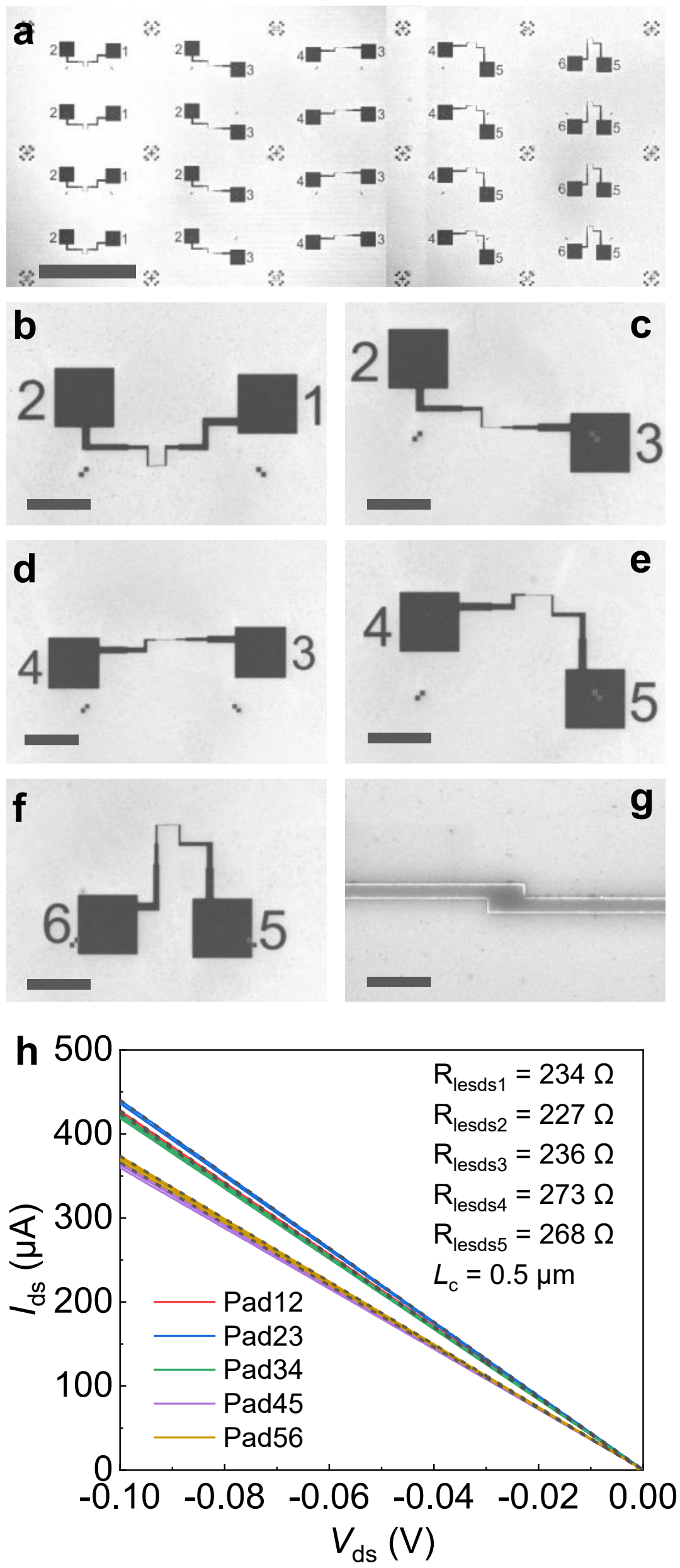

Supplementary Fig. 26. Extraction of the external interconnection metal line resistance. (a) Full SEM image of the structure used for extracting the metal line resistance. Scale bar: 400 μm. **(b-f)** SEM images of different channel lengths in the TLM structure. Scale bar: 60 μm. **(g)** SEM image of the metal interconnection area. Scale bar: 2 μm. **(h)** Collection of current-voltage characteristics used to calculate the lead resistance R_{leads} for FETs with a contact length of 0.5 μm. The metal wire resistance is deducted when extracting the contact resistance of an FET[8].

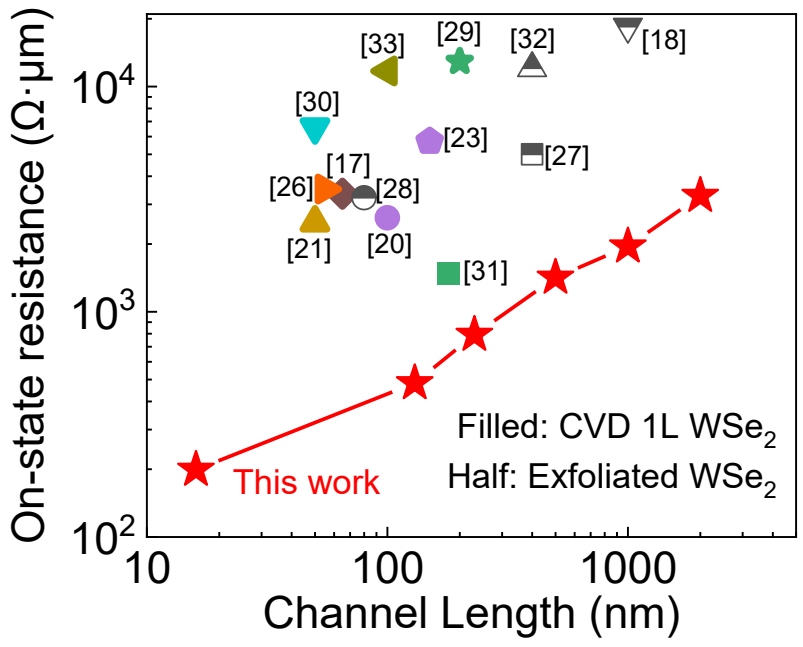

Supplementary Fig. 27. Benchmarking on-state resistance of p-type WSe_2 transistors.

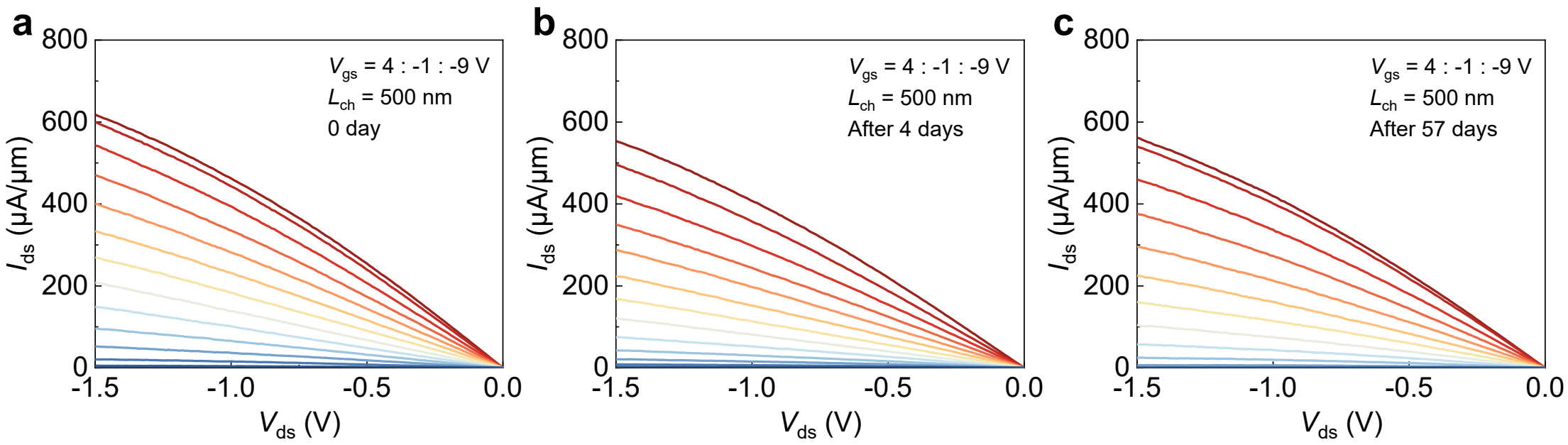

Supplementary Fig. 28. Stability of OM-WSe_2 transistors. Output characteristics of the WSe_2 transistor (L_{ch} = 500 nm) at once **(a)** and after 4 days **(b)** and 57 days **(c)**.

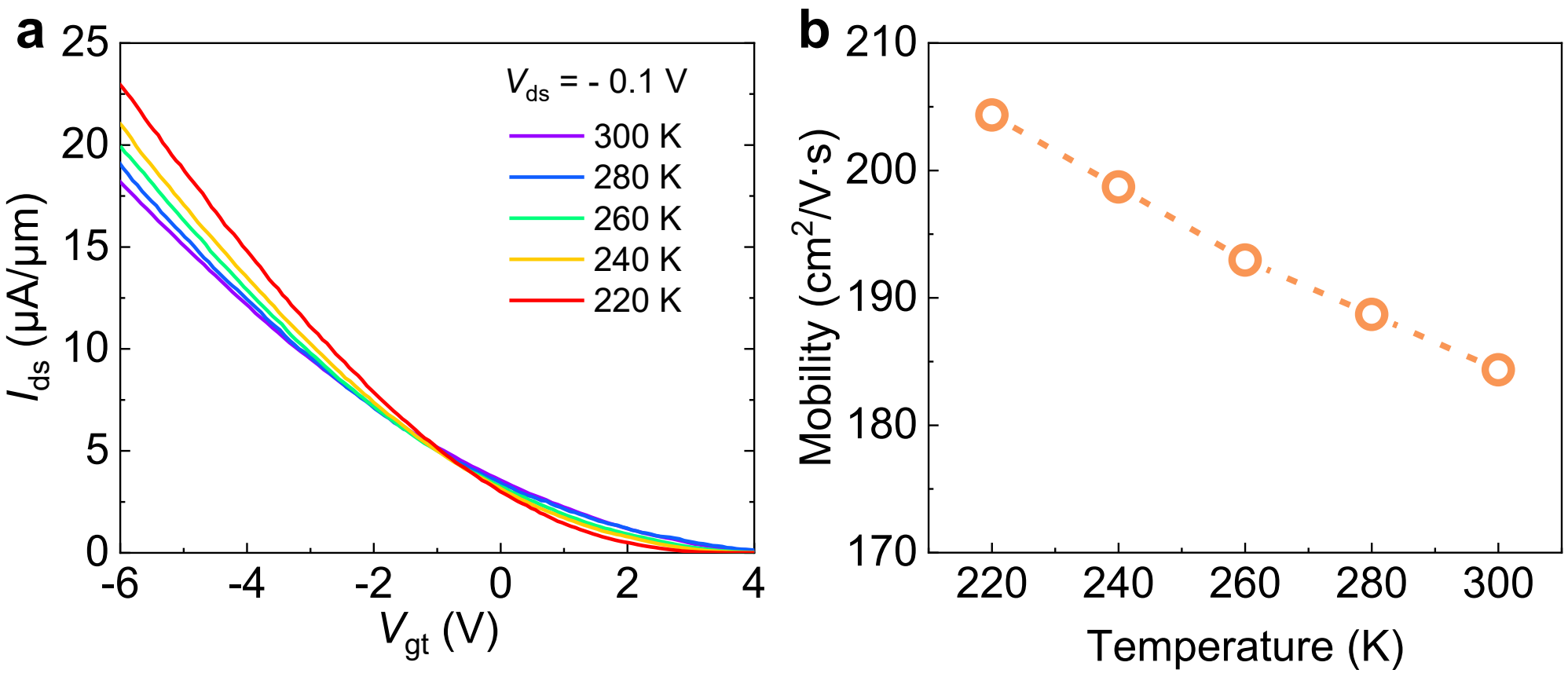

Supplementary Fig. 29. Temperature-dependent transfer characteristics (a) and mobility (b) of the monolayer WSe_2 transistor with doping last, L_{ch} = 1 μm.

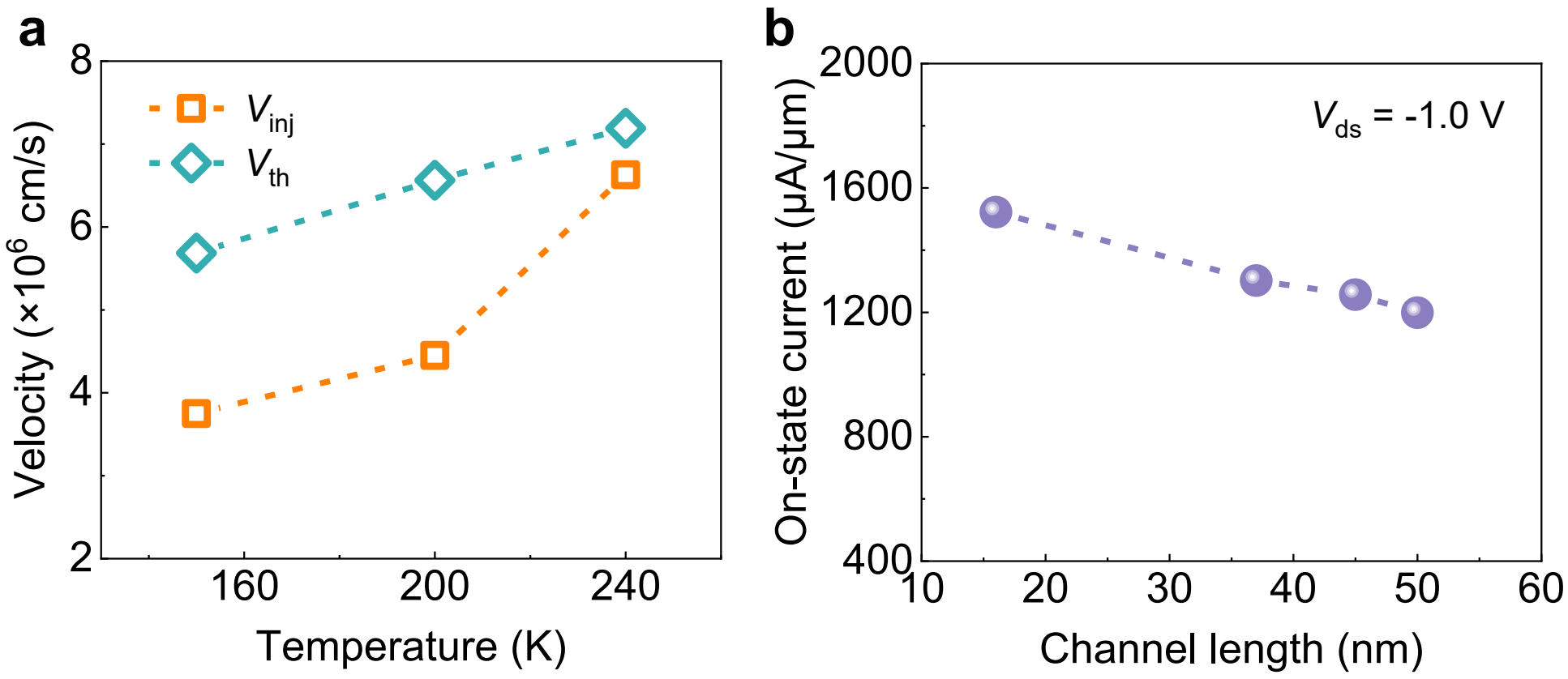

Supplementary Fig. 30. (**a**) Temperature-dependent carrier injection velocity and thermal velocity. (**b**) Channel-length dependence of current in short-channel devices.

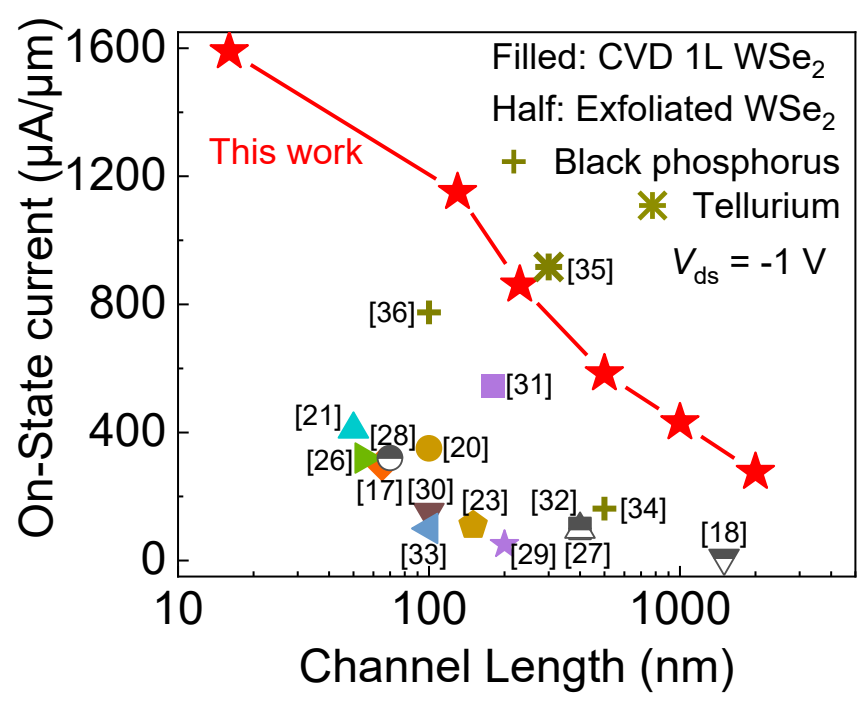

Supplementary Fig. 31. Benchmarking on-state current of p-type WSe_2 transistors.

Supplementary Table 1 Benchmarking state-of-art WSe_2 p-type transistors

Channel	Synthesis method	Contact	Dielectric	L_{ch} (μm)	R_C (Ω·μm)	I_{on} (μA/μm) V_{ds}=-1 V	Mobility ($cm^2/V\cdot s$)	On/off ratio	Ref.
1L WSe_2	CVD	Pt	15 nm SiO_2	2<br>0.016	104	276<br>1532	218<br>-	10^{10}<br>10^7	This work
ML WSe_2	Exfoliated	2D/2D	40 nm hBN/280 nm SiO_2	0.27	300	320	200	~10^9	9
1L WSe_2<br>3L WSe_2	Exfoliated	WO_x	285 nm SiO_2	-	-<br>1400	-	50<br>31	~10^7	10
1L WSe_2	CVD	Au	285 nm SiO_2	-	-	-	143	~10^7	11
2L WSe_2	Exfoliated	Pd	hBN/SiO_2	-	3500	-	195	~10^6	12
2L WSe_2	CVD	VSe_2	285 nm SiO_2	1.8	-	183	137	~10^7	13
ML WSe_2	Exfoliated	Ti	SiO_2	-	109600	-	72.9	~10^8	14
1L WSe_2<br>2L WSe_2	CVD	Pt	300 nm SiO_2	-<br>-	-<br>-	-<br>-	92<br>145	-<br>~10^7	15
2L WSe_2	CVD	Pt	6 nm SiO_2	0.12	650	425	90	~10^7	16
1L WSe_2	CVD	Ti/Pd	2.8 nm HfO_2	0.065	950	300	35	~10^6	17
ML WSe_2	Exfoliated	Pt	300/100 nm SiO_2	1.5	3300	7.6	190	~10^7	18
ML WSe_2	Exfoliated	Ti	300 nm SiO_2	-	-	-	100	~10^6	19
1L WSe_2	CVD	Pt	5 nm SiO_2	0.1	-	350	57	~10^7	20
1L WSe_2	CVD	Pd	100 nm SiN_x	0.05	720	410	30	~10^7	21
1L WSe_2<br>2L WSe_2	CVD	Pt	300 nm SiO_2	-<br>0.7	-<br>-	-<br>156	72.8<br>103.5	-<br>~10^8	22
1L WSe_2	CVD	Ti/Pd	6 nm HfO_2	0.15	6800	110	44	~10^9	23
2L WSe_2	MOCVD	Pd	10 nm HfO_2	0.1	1300	421	16.1	~10^7	24
1L WSe_2<br>2L WSe_2	AACVD	Pt	100 nm Si_3N_4	-<br>-	7000<br>5600	-<br>-	105<br>119	-<br>-	25
1L WSe_2<br>2L WSe_2	CVD	Ti/Pt	3.5 nm HfO_2	0.055	875<br>390	300<br>448	80<br>-	~10^9<br>~10^6	26
ML WSe_2	Exfoliated	Pt or Pd	300 nm SiO_2	0.4	800	100	-	~10	27
ML WSe_2	Exfoliated	Ti/Pd	90 nm SiO_2	0.07	528	320	-	~10^7	28
1L WSe_2	MOCVD	Ru	-	0.2	2700	50	-	~10^7	29
1L WSe_2	CVD	Sb/Pt	100 nm SiN_x	0.1	750	151	-	~10^6	30
1L WSe_2	CVD	Pt	6 nm HfO_2	0.18	875	546	-	~10^9	31
ML WSe_2	Exfoliated	Pt	8 nm HfO_2	0.4	5700	97	-	~10^{10}	32
1L WSe_2	MOCVD	Ru	-	0.1	-	100	-	~10^8	33

Supplementary References

1. S. Barja *et al.*, Identifying substitutional oxygen as a prolific point defect in monolayer transition metal dichalcogenides. *Nature Communications* **10**, 3382 (2019).

2. G. H. Ahn *et al.*, Strain-engineered growth of two-dimensional materials. *Nature Communications* **8**, 608 (2017).

3. J. A. Yang *et al.*, Biaxial Tensile Strain Enhances Electron Mobility of Monolayer Transition Metal Dichalcogenides. *ACS Nano* **18**, 18151-18159 (2024)

4. J. S. Ross *et al*., Electrical control of neutral and charged excitons in a monolayer semiconductor. *Nature Communications* **4**, 1474 (2013).

5. Z. Xiao *et al*., Point defect limited carrier mobility in 2D transition metal dichalcogenides. *ACS Nano* **18**, 8511-8516 (2024).

6. Y. Wang *et al*., Critical challenges in the development of electronics based on two-dimensional transition metal dichalcogenides. *Nature Electronics* **7**, 638-645, (2024).

7. W. Li *et al*., Approaching the quantum limit in two-dimensional semiconductor contacts. *Nature* **613**, 274–279 (2023).

8. J. Jiang *et al*., Ballistic two-dimensional InSe transistors. *Nature* **616**, 470–475 (2023).

9. H.-J. Chuang *et al*., Low-Resistance 2D/2D Ohmic Contacts: A Universal Approach to High-Performance WSe_2, MoS_2, and $MoSe_2$ Transistors. *Nano Letters* **16** (3), 1896-1902 (2016).

10. M. Yamamoto, *et al.*, Self-Limiting Oxides on WSe_2 as Controlled Surface Acceptors and Low-Resistance Hole Contacts. *Nano Letters* **16** (4), 2720-2727 (2016).

11. Y. Gao *et al.*, Ultrafast growth of high-quality monolayer WSe_2 on Au. *Advanced Materials* **29**, 1700990 (2017).

12. Y. Jung *et al.*, Transferred via contacts as a platform for ideal two-dimensional transistors. *Nature Electronics* **2**, 187–194 (2019).

13. J. Li *et al.*, General synthesis of two-dimensional van der Waals heterostructure arrays. *Nature* **579**, 368–374 (2020).

14. S. Yang *et al.*, Selective p-Doping of 2D WSe_2 via UV/Ozone Treatments and Its Application in Field-Effect Transistors. *ACS Applied Materials & Interfaces* **13** (1), 955-961 (2021).

15. Z. Zhang *et al.*, Highly Selective Synthesis of Monolayer or Bilayer WSe_2 Single Crystals by Pre-annealing the Solid Precursor. *Chemistry of Materials* **33** (4), 1307-1313 (2021).

16. X. Shi *et al.*, High-Performance Bilayer WSe_2 pFET with Record I_{ds} = 425 μA/μm and G_m = 100 at μS/μm V_{ds} = -1 V By Direct Growth and Fabrication on SiO_2 Substrate. *2022 International Electron Devices Meeting (IEDM)*. (2022), pp. 7.1.1-

7.1.4.

17. C. -C. Chiang *et al*., Air-Stable P-Doping in Record High-Performance Monolayer WSe_2 Devices, *IEEE Electron Device Letters* **43**, 319-322 (2022).

18. Y. Wang *et al*., P-type electrical contacts for 2D transition-metal dichalcogenides. *Nature* **610,** 61–66 (2022).

19. H. Cho *et al*., Se-Vacancy Healing with Substitutional oxygen in WSe_2 for High-Mobility p-Type Field-Effect Transistors. *ACS Nano* **17** (12), 11279-11289 (2023).

20. X. Wang *et al*., BEOL Compatible High-Performance Monolayer WSe_2 pFETs with Record Gm=190 μS/μm and Ion=350 μA/μm by Direct-Growth on SiO_2 Substrate at Reduced Temperatures. *2023 International Electron Devices Meeting (IEDM).* (2023), pp. 1-4.

21. A. -S. Chou *et al*., Status and Performance of Integration Modules Toward Scaled CMOS with Transition Metal Dichalcogenide Channel. *2023 International Electron Devices Meeting (IEDM).* (2023), pp. 1-4.

22. K. S. Kim *et al*., Non-epitaxial single-crystal 2D material growth by geometric confinement. *Nature* **614**, 88–94 (2023).

23. Y. Tan *et al*., Monolayer WSe_2 Field-Effect Transistor Performance Enhancement by Atomic Defect Engineering and Passivation. *ACS Nano* **19** (9), 8916-8925

(2025).

24. S. Ghosh *et al*., High-performance p-type bilayer WSe_2 field effect transistors by nitric oxide doping. *Nature Communications* **16,** 5649 (2025).
25. S. -A. Chou *et al*., Large-scale alkali-assisted growth of monolayer and bilayer WSe_2 with a low defect density. *Nature Communications* **16**, 2777 (2025).
26. H. -Y. Lan *et al*., Uncovering the doping mechanism of nitric oxide in high-performance P-type WSe_2 transistors. *Nature Communications* **16**, 4160 (2025).
27. L. Cai *et al*., Rapid Flame Synthesis of Atomically Thin MoO_3 down to Monolayer Thickness for Effective Hole Doping of WSe_2. *Nano Letters* **17** (6), 3854-3861 (2017).
28. C. -S. Pang *et al*., Atomically Controlled Tunable Doping in High-Performance WSe_2 Devices. *Advanced Electronic Materials* **6**, 1901304 (2020).
29. K. P. O'Brien *et al*., Advancing 2D Monolayer CMOS Through Contact Channel and Interface Engineering. *2021 IEEE International Electron Devices Meeting (IEDM)*. (2021), pp. 7.1.1-7.1.4.
30. A. -S. Chou *et al*., High-Performance Monolayer WSe_2 p/n FETs via Antimony-Platinum Modulated Contact Technology towards 2D CMOS Electronics. *2022 International Electron Devices Meeting (IEDM)*. (2022), pp. 7.2.1-7.2.4.

31. H. -Y. Lan *et al.*, Wafer-scale CVD Monolayer WSe_2 p-FETs with Record-high 727 μA/μm I_{on} and 490 μS/μm g_{max} via Hybrid Charge Transfer and Molecular Doping. *2023 International Electron Devices Meeting (IEDM)*. (2023), pp. 1-4.
32. N. H. Patoary *et al.*, Improvements in 2D p-type WSe_2 transistors towards ultimate CMOS scaling. *Scientific Reports* **13**, 3304 (2023).
33. K. Maxey *et al.*, 300 mm MOCVD 2D CMOS Materials for More (Than) Moore Scaling. *2022 IEEE Symposium on VLSI Technology and Circuits (VLSI Technology and Circuits)*, (2022), pp. 419-420.
34. X. Xiong *et al.*, Nonvolatile Logic and Ternary Content-Addressable Memory Based on Complementary Black Phosphorus and Rhenium Disulfide Transistors. *Advanced Materials* **34**, 2106321 (2022).
35. Y. Wang *et al.*, Field-effect transistors made from solution-grown two-dimensional tellurene. *Nature Electronics* **1**, 228–236 (2018).
36. X. Li *et al.*, High-speed black phosphorus field-effect transistors approaching ballistic limit. *Science Advance* **5**, eaau3194 (2019).
