# Supplementary material for: Low contact resistance and high current density in ballistic p-type transistors based on oxygen-doped WSe2 monolayers
Source: Nat Commun. 2026 Oct 1;17:11273. doi: 10.1038/s41467-026-78157-w (PMC13720060; doi:10.1038/s41467-026-78157-w)
Supplement: Supplementary file 2 [file 41467_2026_78157_MOESM2_ESM.pdf]

nature portfolio

Peer Review File

Low contact resistance and high current density in ballistic p-type transistors based on oxygen-doped WSe2 monolayers

Corresponding Author: Professor Xuefei Li

This file contains all reviewer reports in order by version, followed by all author rebuttals in order by version.

Version 0:

Reviewer comments:

Reviewer #1

(Remarks to the Author)
Sun et al. present a controllable oxygen-doping method to reduce the density of Se vacancy defects in CVD-grown WSe_2. The effectiveness of the doping is confirmed by STM measurements, showing a reduction in Se vacancy density from 3.9 e11 cm-2 to 1.1 e11 cm-2. The authors further claim that this method reduces the hole contact resistance from 1869 Ω·μm to a record-low value of 104 Ω·μm. However, the doping mechanisms seem unclear and the key experimental details require further clarification. Therefore, I suggest major revision of the manuscript.
Here are some specific comments to improve their manuscript.
1. The reported doping levels appear to be controlled by varying the oxygen concentration during the post-device annealing process. The mobility of monolayer WSe2 increases with increasing oxygen concentration (Figure s9). What is the behavior at higher doping levels?
2. In Fig. 2a, the Raman spectrum of monolayer WSe2 exhibits a pronounced background and unusually broad peaks prior to annealing. After annealing, the Raman signal is significantly improved, suggesting enhanced crystallinity. Could the authors comment on the structural evolution of WSe2 during the annealing process?
3. Figure S5 & S6, in monolayer WSe2, both positive and negative trions can coexist, making it difficult to unambiguously infer the doping type and level solely from the FWHM of the PL spectra (Figs. S5 and S6). Gate-dependent PL measurements are therefore required to confirm the doping effect and to distinguish between electron and hole trion contributions.
4. The device characteristics are reported for as-grown samples on SiO2 substrates. Given that the growth process involves high temperatures and the use of KCl, it is important to clarify whether this process induces degradation or leakage in the 15 nm SiO2 dielectric. The authors should provide C–V measurements and dielectric leakage data both after growth and after the post-annealing process.
5. Could the authors comment on how the oxygen passivates the defects of monolayer WSe2 if contacts have already been made on the monolayer WSe2?
6. The authors should provide more date statics for doping first and doping last strategies. The two transfer curves do not show significant differences, which could arise from variations in devices.
7. The difference in threshold voltage between Fig. 4a and Fig. S19 cannot be attributed to EOT, as the drain current at zero gate voltage already shows a significant discrepancy between the two devices.
8. What is the mobility of device using HfO2 back gate?
9. The authors are advised to provide XPS measurements of monolayer WSe2 to directly confirm the chemical composition of the samples before and after doping.

Reviewer #2

(Remarks to the Author)
This study remarkably improves the chronic issues of high contact resistance (Rc) and low mobility in 2D WSe2 p-type transistors using oxygen doping and a 'Full-contact' structure. Notably, achieving a contact resistance of 104~ and a ballistic ratio of 81% marks a significant advancement for next-generation 2D CMOS technology. The clear elucidation of the selenium vacancy (VSe) repair mechanism via DFT calculations and physical analysis (STM, Raman, PL) is also commendable. However, further refinements regarding device operation modes and stability are necessary for practical logic circuit applications.

1. According to the transfer characteristics in Fig. 2f, oxygen doping induces a significant positive shift in the threshold voltage (Vth), resulting in depletion-mode (normally-on) operation. Given that enhancement-mode (normally-off) characteristics are essential for minimizing static power consumption in practical CMOS integrated circuits, what strategies do the authors propose to shift the Vth toward negative values? Specifically, how can enhancement-mode operation be achieved while preserving the record-high mobility and ultralow contact resistance demonstrated in this work (e.g., through gate work-function engineering or finer control of oxygen concentration)?
2. The oxygen doping is performed via thermal annealing. How stable is the electrical performance of the OM-WSe2 devices when exposed to ambient air for a long period?

3. The 'doping last' process shows significantly lower contact resistance than 'doping first'. Is there any evidence that oxygen atoms diffuse underneath the metal contacts to repair defects at the buried interface?

4. The paper mentions that WSe2 performance is superior on SiO2 due to an additional doping effect. Have you observed any degradation in mobility or subthreshold swing (SS) when using high-k dielectrics like HfO2?

5. While CVD-grown WSe2 was used, the device-to-device variation across a large scale (e.g., 2-inch wafer) is critical. What is the standard deviation of the mobility and Rc values across the wafer?

Reviewer #3

(Remarks to the Author)
Lei Sun et al. proposed a new oxidation method for monolayer WSe2 to fabricate a ballistic p-type transistor. The method and results appear novel and promising. However, to meet the scientific rigor, reproducibility, and clarity expected by the journal, several issues need to be addressed before its publication:

Major points.
1. A key concern is the reproducibility of the experimental procedure. The device fabrication process should be described in more detail. For example, the manuscript does not explain how the oxygen concentration during annealing (~2x10^-4 mol/L) was measured, either in the main text or in the Methods. Was oxygen introduced only at the maximum annealing temperature of 200 degree C? In addition, what happens if the oxygen concentration during annealing exceeds ~2x10^-4 mol/L? In previous studies involving oxygen doping of WSe2, the entire WSe2 layer was typically converted or oxidized (e.g., https://ieeexplore.ieee.org/document/9046453 , and so on). In contrast, the mild oxidation used in this work appears quite unique and interesting. From this perspective, it would be helpful if the authors could explain more clearly how pure oxygen annealing can selectively passivate selenium vacancies without converting the entire monolayer. Similarly, details on the Pt S/D electrode deposition are necessary. Under what vacuum level was the deposition performed? What were the source-substrate distance and deposition rate? Is there any passivation layer on top of the Pt electrode?
2. Regarding Fig. 3, the mechanism behind the performance differences between the “doping first” and “doping last” processes is not sufficiently explained. Supplementary Fig. 13 shows a schematic comparison of the two conditions, suggesting that contact doping may differ. However, such discussion is largely missing from the main text. Moreover, in the doping last process, since the contact electrodes act as a mask, oxygen doping is likely to occur more in the channel region than underneath the contacts. In that case, one would expect the doping last devices to show inferior performance. Without a materials-level characterization of the contact interface, the current explanation feels not fully convincing.
3. It would also strengthen the manuscript to investigate the temperature dependence of device properties. From the conductivity trend, the device seems to exhibit a negative thermionic emission barrier for Vg < 2 V and undergoes a metal-to-insulator transition (MIT), as shown in Fig. S15. Compared to previously reported WSe2 systems, is the MIT less or more pronounced? How does the mobility change with temperature? Is the transport limited by contact scattering?
4. To determine whether the WSe2 FETs truly operate in the ballistic regime, it is essential to extract the saturation velocity and examine its temperature dependence. This evaluation is currently missing. Furthermore, ballistic transport should be demonstrated through channel-length scaling that shows reduced scattering as the device dimensions shrink. Such evidence is not presented in the manuscript.

Minor points.
5. The notation for gate voltages is not consistent across the figures. For example, Vgt, Vbg, and other symbols are used in different panels.
6. In Figs. 2f and 2h, the information on Vds and Vgs is missing. The figure captions should be revised.
7. For Figs. 3d and 3e, the Vg conditions are not provided.
8. In Supplementary Fig. 16, I suggest quantifying the linearity as a function of Vg and providing the corresponding figures (e.g., linearity metrics) rather than only qualitative plots.
9. On line 252-254, “To further evaluate the potential of WSe2 pFETs with doping last, we scaled the channel length down to 16 nm on monolayer WSe2, which channel length was confirmed by high-resolution cross-sectional transmission electron microscopy (TEM) image (Supplementary Figs. 17–18).” However, there is no actual TEM image shown; only EDS data are presented in Fig. S18. It also appears that the current Fig. S17 may have been placed incorrectly.

Version 1:

Reviewer comments:

Reviewer #1

(Remarks to the Author)
The authors have addressed most of the comments. However, they have not yet provided C–V measurements of the dielectrics both after growth and after the post-annealing process. This information is important because changes in the dielectric capacitance can directly affect the extracted mobility values.
The authors should provide the C–V data used for mobility extraction and clearly describe the step-by-step procedure used to calculate the mobility, including how the gate capacitance was determined, which transfer-curve regime was used, and whether the same capacitance value was assumed before and after post-annealing.

Reviewer #2

(Remarks to the Author)
This manuscript presents an intriguing approach to mitigating the chronic issue of high selenium vacancy density in monolayer WSe_2 via a controlled oxygen-moderate annealing process, which, combined with a full-contact configuration, delivers exceptional hole mobility and ultralow contact resistance. The authors have made commendable efforts to address the first-round review comments, particularly by introducing Raman and PL profiles of a 3 nm Pt-covered WSe_2 layer to justify their oxygen diffusion mechanism. However, to robustly validate the scalability for practical CMOS integrated circuits and the physical soundness of the proposed diffusion model, several critical open questions must be resolved. Specifically, potential performance degradation factors during device scaling and the underlying scattering mechanisms require a deeper investigation. Therefore, I recommend further clarification on the following points.

1. Please provide quantitative subthreshold swing (SS) data before and after oxygen annealing for the HfO_2-gated devices to clarify how the oxygen incorporation affects remote Coulomb scattering and native high-k interface traps (Dit)
2. If oxygen laterally diffuses from the contact edges into the channel during the 'doping-last' process, it could create a doping concentration gradient. Do you have experimental data tracking Vth shifts as a function of Lch to rule out this profile non-uniformity?

Reviewer #3

(Remarks to the Author)
The authors have revised the manuscript thoroughly and have addressed all the issues I raised. I believe this work is both highly timely and novel. I therefore recommend its publication in Nature Communications.

Version 2:

Reviewer comments:

Reviewer #1

(Remarks to the Author)
The authors have addressed my comments.

Reviewer #2

(Remarks to the Author)
The authors have provided additional clarification regarding the possible oxygen diffusion pathway. However, the current Vth -versus- Lch analysis does not fully rule out a possible channel-length-dependent doping profile, since Vth itself is influenced by short-channel effects.

It would therefore be helpful if the authors could provide the annealing-induced threshold-voltage shift, delta Vth = Vth,after - Vth, before, or an equivalent analysis that more directly evaluates the uniformity of oxygen doping. If such data are not available, the statement that the doping profile is highly uniform should be moderated accordingly.

Open Access This Peer Review File is licensed under a Creative Commons Attribution 4.0 International License, which permits use, sharing, adaptation, distribution and reproduction in any medium or format, as long as you give appropriate credit to the original author(s) and the source, provide a link to the Creative Commons license, and indicate if changes were made.
In cases where reviewers are anonymous, credit should be given to 'Anonymous Referee' and the source.
The images or other third party material in this Peer Review File are included in the article's Creative Commons license, unless indicated otherwise in a credit line to the material. If material is not included in the article's Creative Commons license and your intended use is not permitted by statutory regulation or exceeds the permitted use, you will need to obtain permission directly from the copyright holder.
To view a copy of this license, visit https://creativecommons.org/licenses/by/4.0/

.++Point-by-point response to reviewers' comments

To the reviewers:

We are grateful to the reviewers for their time and very helpful comments. In response, we have included more experimental results and analysis. We have made changes to the text and, accordingly, the manuscript has been improved in several respects. In the revised manuscript, we have incorporated all the suggested changes and clarifications. We believe that we have been able to address all the concerns of the reviewers.

The key changes in the modified manuscript are as follows:

1. Electrical and PL properties of WSe_2 with higher doping levels
2. X-ray photoelectron spectroscopy (XPS) and electrical properties of monolayer WSe_2 before and after annealing
3. Raman and PL measurements of monolayer WSe_2 covered with 3 nm Pt
4. Standard deviation of the mobility and R_c values across the large scale WSe_2
5. Temperature-dependent mobility, carrier injection velocity, and thermal velocity

In summary, we greatly appreciate the reviewer's insightful comments and valuable suggestions, which helped us clarify many issues in the original manuscript and strengthened the manuscript with these new discussions. Thus, by incorporating these new data and analyses, we believe that we have now addressed the reviewer's concerns.

The responses to the reviewer's comments are listed below.

~~~~~~
~~~~~~

Reviewers' comments:

<u>Reviewer #1 (Remarks to the Author):</u>

Sun et al. present a controllable oxygen-doping method to reduce the density of Se vacancy defects in CVD-grown WSe_2. The effectiveness of the doping is confirmed by STM measurements, showing a reduction in Se vacancy density from 3.9 e11 cm^{-2} to 1.1 e11 cm^{-2}. The authors further claim that this method reduces the hole contact resistance from 1869 Ω • μm to a record-low value of 104 Ω·μm. However, the doping mechanisms seem unclear and the key experimental details require further clarification. Therefore, I suggest major revision of the manuscript.

We appreciate the reviewer's comments, and the article has been revised to comply with the reviewer's suggestions.

The detailed questions are as follows:

1. **The reported doping levels appear to be controlled by varying the oxygen concentration during the post-device annealing process. The mobility of monolayer WSe_2 increases with increasing oxygen concentration (Figure S9). What is the behavior at higher doping levels?**

We thank the reviewer for pointing this out. We fabricated monolayer WSe_2 transistors with increasing oxygen concentration. The mobility of the transistors decreased and the number of defects increased at higher doping levels, as shown in Fig. R1. The data have been updated in the new Fig. 2d and Supplementary Fig. 8 and 12a.

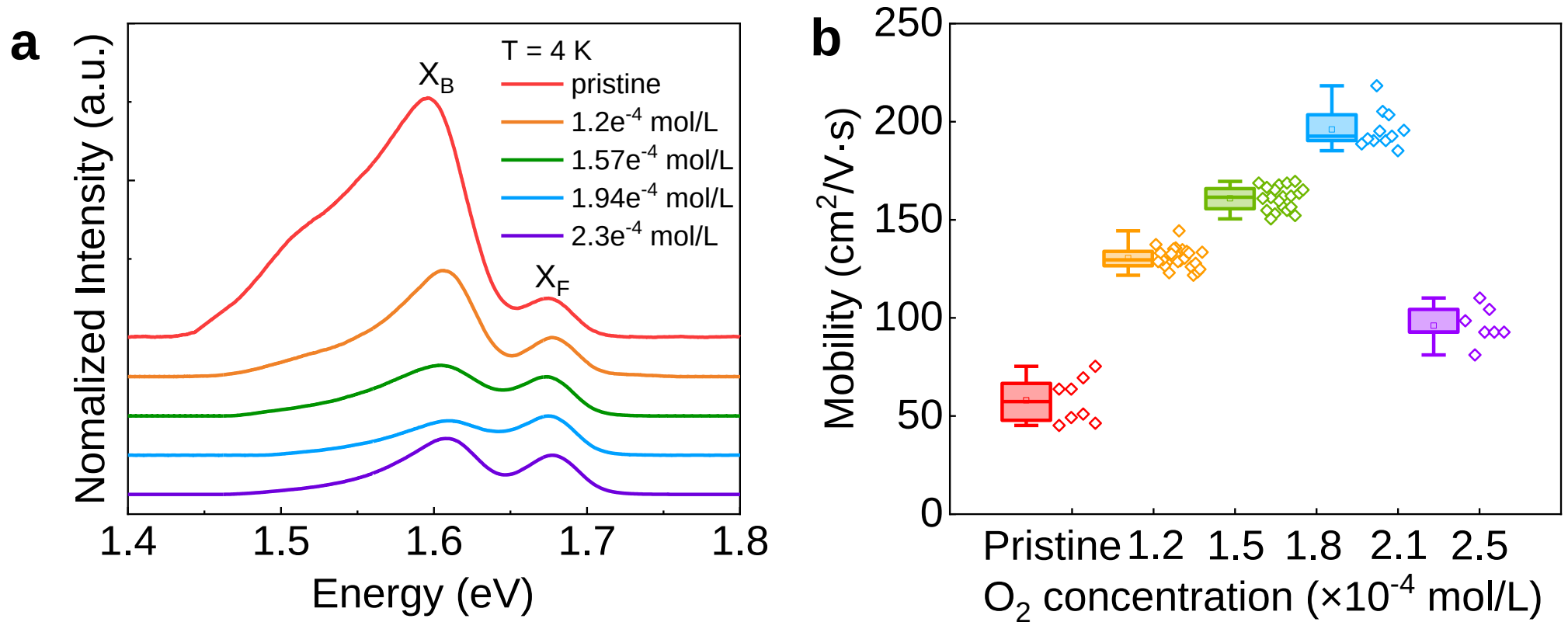

Fig. R1. (a) PL at 4 K without and with different O_2 concentrations doping with WSe_2, (b) Field-effect mobility statistics of WSe_2 transistors without and with different O_2 concentrations doped at room temperature.

2. In Fig. 2a, the Raman spectrum of monolayer WSe_2 exhibits a pronounced background and unusually broad peaks prior to annealing. After annealing, the Raman signal is significantly improved, suggesting enhanced crystallinity. Could the authors comment on the structural evolution of WSe_2 during the annealing process?

We thank the reviewer for pointing this out. The as-prepared WSe_2 monolayer exhibits a pronounced background and unusually broad peaks before annealing, which can be attributed to the presence of a high density of defects generated during the growth process. The elevated background is typically associated with strong photoluminescence (PL) originating from defect states or impurity levels within the bandgap, which are abundant in as-grown crystals (*Nanotechnology* 2019, 30, 034001). Proper annealing with oxygen fills up the Se vacancies, which leads to improved local structure integrity and thus improved PL intensity. However, annealing with high oxygen concentration also causes the formation of new defects as shown in Supplementary Fig. 8. We have added a discussion in the revised manuscript as follows:

"annealing in an appropriate oxygen concentration is very important for reducing the number of defect states and improving the crystallinity of monolayer WSe_2"

3. Figure S5 & S6, in monolayer WSe_2, both positive and negative trions can coexist, making it difficult to unambiguously infer the doping type and level solely from the FWHM of the PL spectra (Figs. S5 and S6). Gate-dependent PL measurements are therefore required to confirm the doping effect and to distinguish between electron and hole trion contributions.

We thank the reviewer for this helpful comment. Gate-dependent PL measurements were performed in our previous work (*Nat. Electron.*, accepted, DOI: 10.1038/s41928-026-01637-w), as shown in Fig. R2. The PL emission of free-excitons at higher photon energies (ranging from 1.65 to 1.75 eV) can be deconvoluted by two Gaussian functions. These functions are assigned to charged and neutral A-excitons, respectively (X_A, and X_A^+). As V_{gs} decreases from 0 V to -20 V (more holes are doped into WSe_2), the X_A resonance energy moves to higher energy and its oscillator strength diminishes as the X_A^+ resonance develops a redshift due to Coulomb screening, suggesting that as-grown WSe_2 is initially p-doped.

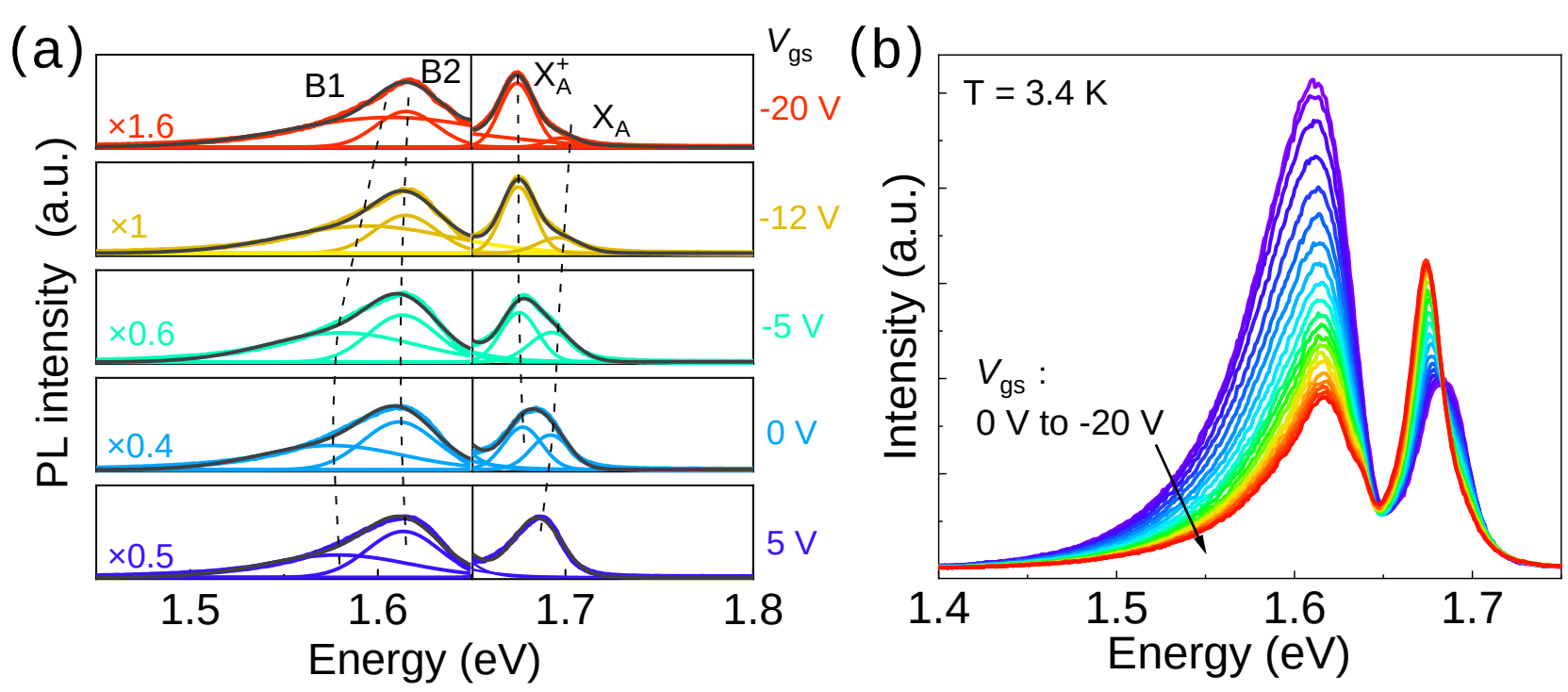

Fig. R2. Gate-dependent PL of as-grown monolayer WSe_2 at 3.4 K. (a) Typical PL curves fitted with four Gaussian peaks, denoted as B1, B2, X_A, and X_A^+. (b) PL spectrum with a gate voltage from 0 V to -20 V, step: -1 V.

4. **The device characteristics are reported for as-grown samples on SiO_2 substrates. Given that the growth process involves high temperatures and the use of KCl, it is important to clarify whether this process induces degradation or leakage in the 15 nm SiO_2 dielectric. The authors should provide C–V measurements and dielectric leakage data both after growth and after the post-annealing process.**

We thank the reviewer for pointing this out. Our SiO_2/Si substrates were purchased directly from the Hefei Kejing Company. In view of the highly doped Si substrates, it is impossible to perform C-V measurements. On the basis of the transistor data, the SiO_2 dielectric property is good. We plotted the gate leakage currents of the devices before and after post-annealing in Fig. R3. It can be seen that the gate leakage current of both devices is always less than 2 nA, which is much lower than that of drain current in the saturation region. This indicates the excellent insulator properties of SiO_2. These data have been added to the revised manuscript as Supplementary Fig. 11.

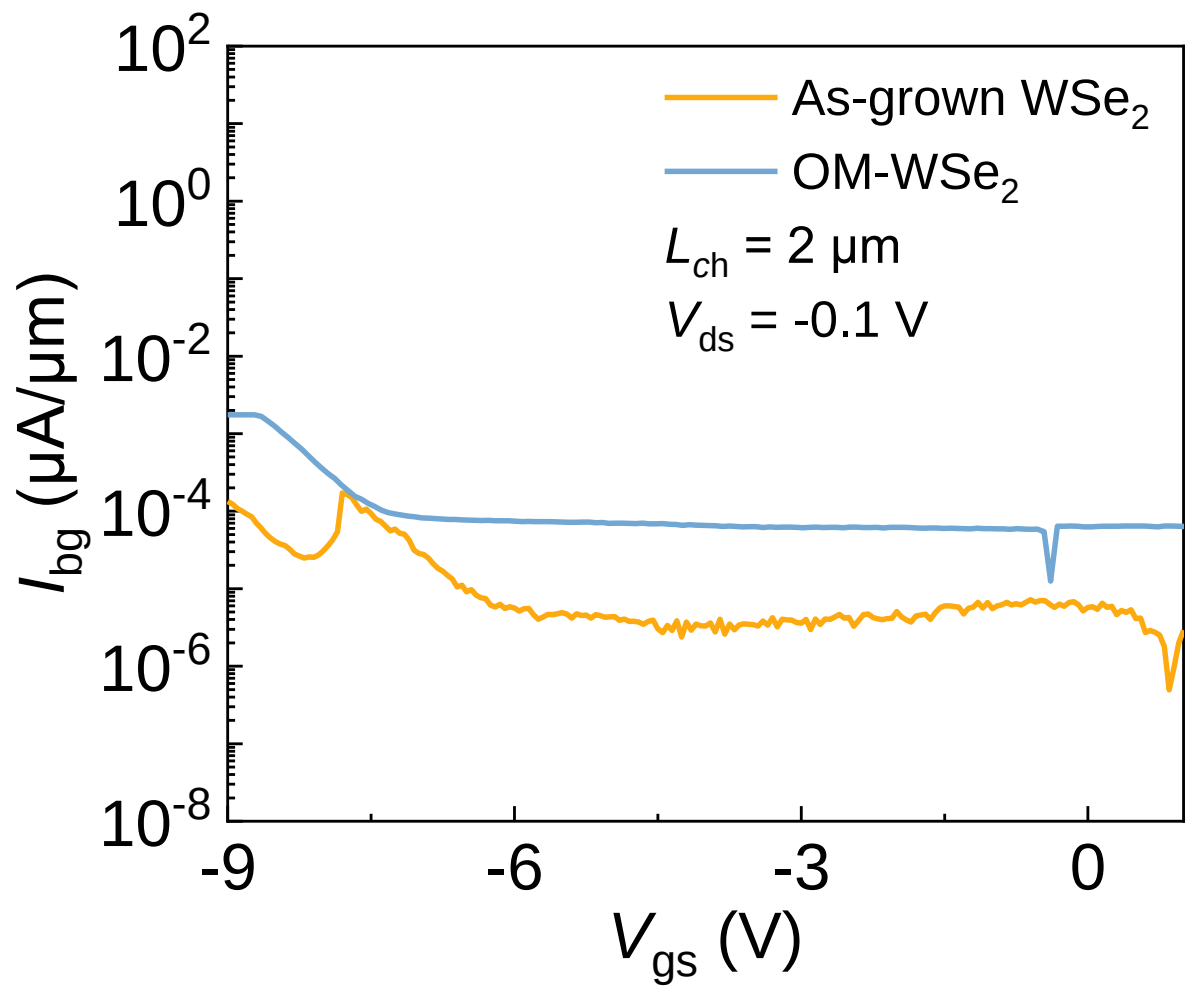

Fig. R3. Gate leakage currents of WSe_2 transistors with and without post-annealing process. The gate dielectric is 15-nm SiO_2.

5. **Could the authors comment on how the oxygen passivates the**

defects of monolayer WSe_2 if contacts have already been made on the monolayer WSe_2?

We thank the reviewer for pointing this out. Raman and PL measurements were conducted on monolayer WSe_2 covered with 3 nm of Pt, and the blueshift of the Raman A_{1g} peak and the PL peak for doping-last confirmed p-type doping, which proves that oxygen atoms diffused underneath the metal contacts and repaired defects at the buried interface (Fig. R4). On the basis of these experimental results, oxygen during vacuum annealing can still effectively passivate defects in the contact regions possibly through metal grain boundaries or lateral diffusion from the channel to the contact regions. This is also well demonstrated by the distinct device characteristics observed for doping-first and doping-last devices. These data have been added to the revised manuscript as Supplementary Fig. 18. We have added a discussion in the revised manuscript as follows:

"Raman and PL spectra confirm oxygen doping in the contact regions, which likely occurs through metal grain boundaries or lateral diffusion from the channel (Supplementary Fig. 18)."

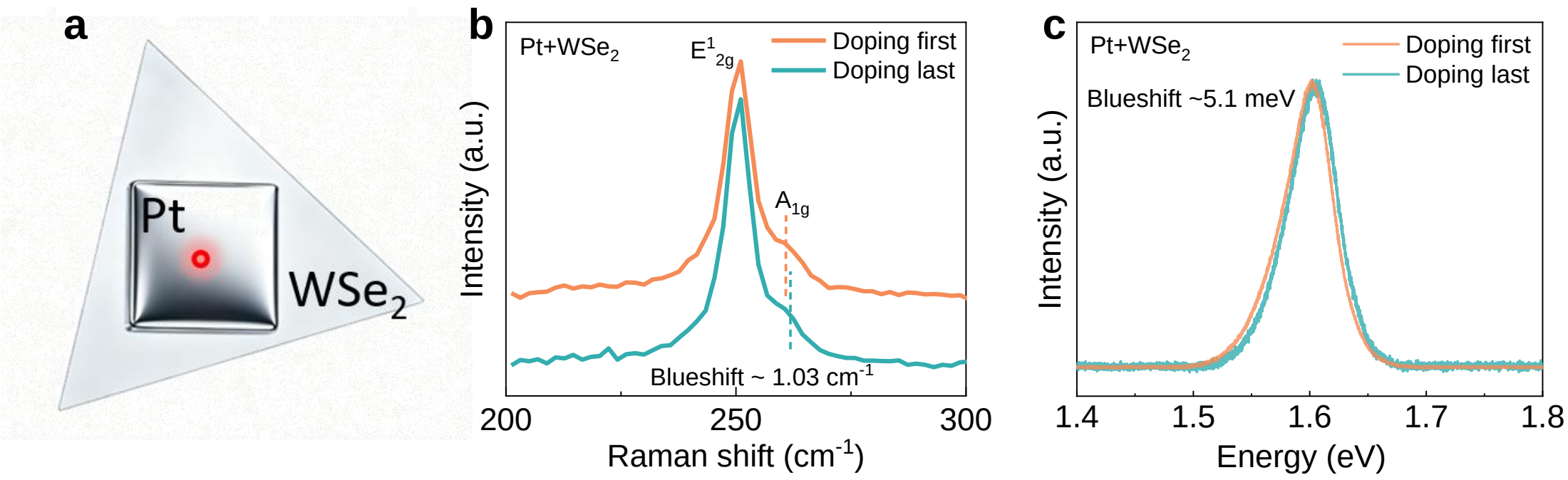

Fig. R4. (a) Schematic diagram of Raman measurement for 3-nm Pt+1L WSe_2, Raman (b) and PL (c) Spectra of Pt+WSe_2 with doping-first and doping-last.

6. The authors should provide more data statics for doping first and doping last strategies. The two transfer curves do not show significant differences, which could arise from variations in devices.

We thank the reviewer for this suggestion. To address this concern, we performed statistical measurements on a large number of devices for both the doping-first and doping-last strategies, as depicted in Fig. R5. The device-to-device variation is comparable between the two groups (similar standard deviations), ruling out the possibility that the lack of visible difference is merely due to random device fluctuations. These data have been added to the revised manuscript as Supplementary Fig. 17.

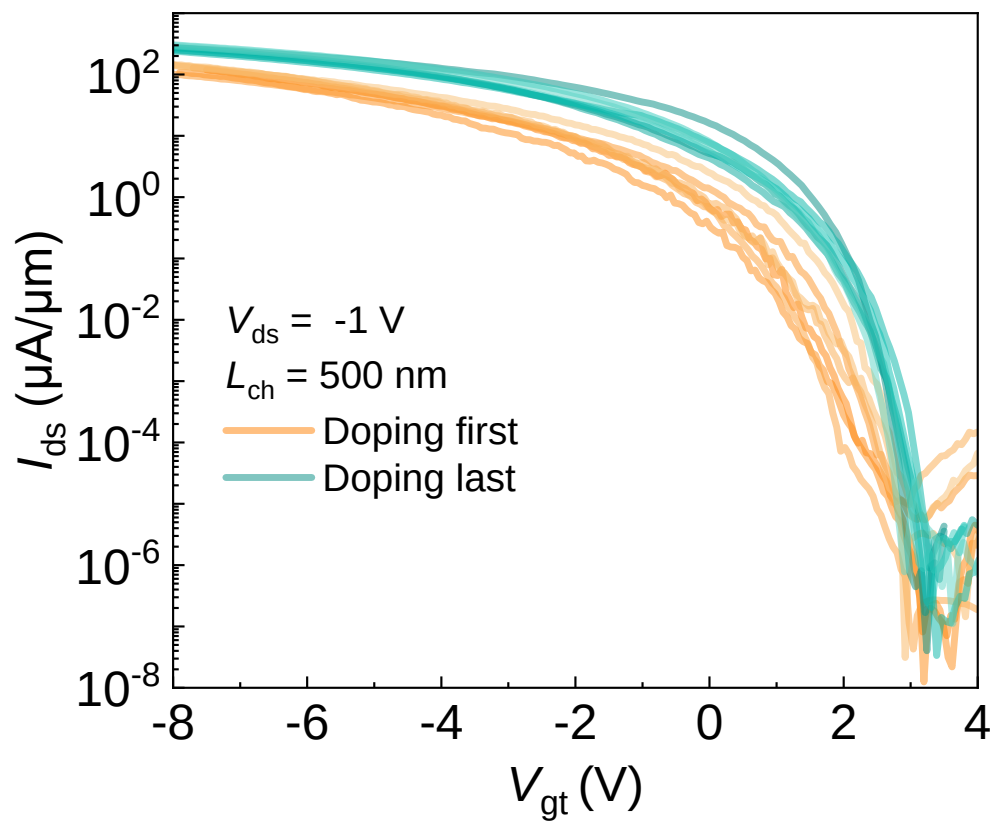

Fig. R5. Transfer characteristics curve statistics data of devices with doping first and doping last.

7. The difference in threshold voltage between Fig. 4a and Fig. S19 cannot be attributed to EOT, as the drain current at zero gate voltage already shows a significant discrepancy between the two devices.

We thank the reviewer for pointing this out. Accordingly, we have removed this content from the revised manuscript.

8. What is the mobility of device using HfO_2 back gate?

We thank the reviewer for pointing this out. According to the formula $\mu = \frac{g_m L_{ch}}{W_{ch} C_{ox} V_{ds}}$, g_m is the transconductance, L_{ch} is the channel length, W_{ch} is the

channel width, C_{ox} is the gate oxide capacitance per unit area (here C_{HfO2} = 2.83 μF/cm^2), and V_{ds} is the drain-source voltage. We calculated that the mobility of device before and after doping was 5.3 and 13 cm^2/V·s, respectively. The data have been added in Supplementary Fig. 24.

9. The authors are advised to provide XPS measurements of monolayer WSe_2 to directly confirm the chemical composition of the samples before and after doping.

We thank the reviewer for pointing this out. We carried out XPS measurements for monolayer WSe_2 before and after doping (OM-WSe_2). The survey spectra, W 4f, and Se 3d of WSe_2 before and after doping are shown below (Fig. R6). It can be seen that the change of chemical composition of the samples before and after doping is not obvious. We believe the doping concentration is lower than the XPS detection limit; however, such doping level is sufficient to improve the electrical characteristics. These data have been added to the revised manuscript as Supplementary Fig. 10. We have added a discussion in the revised manuscript as follows:

X-ray photoelectron spectroscopy (XPS) show that the chemical composition of WSe_2 before and after doping remains unchanged (Supplementary Fig. 10).

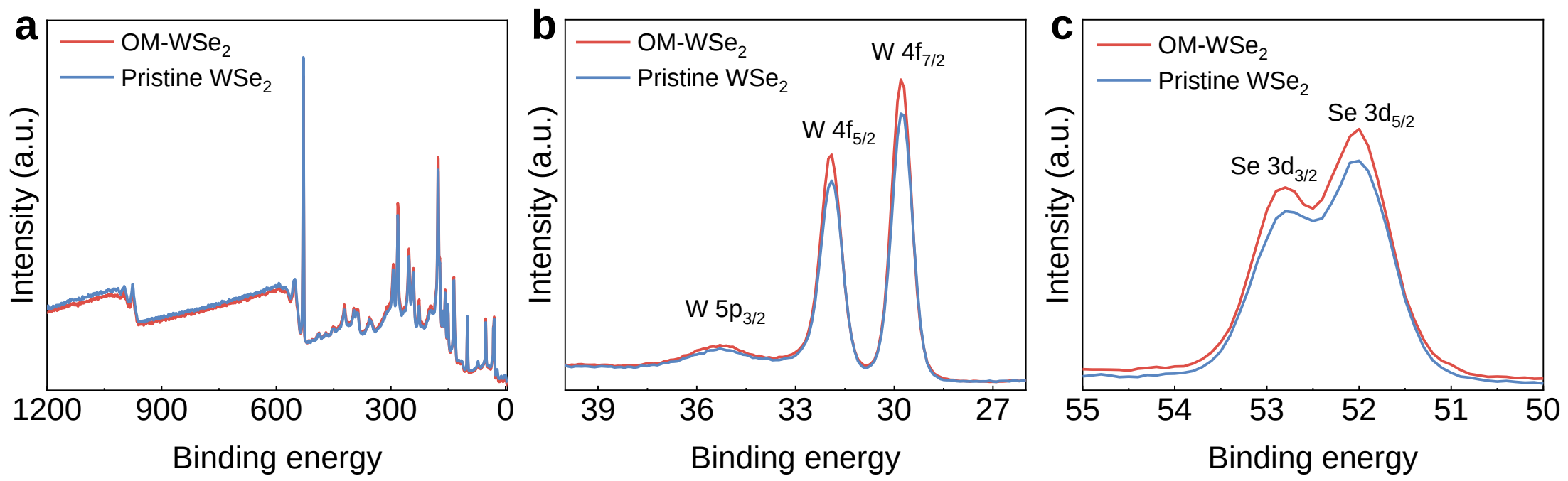

Fig. R6. XPS spectra of WSe_2 before and after doping: (a) Survey spectrum, (b) W 4f, (c) Se 3d.

~~~~~~
~~~~~~

Reviewer #2 (Remarks to the Author):

This study remarkably improves the chronic issues of high contact resistance (R_c) and low mobility in 2D WSe_2 p-type transistors using oxygen doping and a 'Full-contact' structure. Notably, achieving a contact resistance of 104~ and a ballistic ratio of 81% marks a significant advancement for next-generation 2D CMOS technology. The clear elucidation of the selenium vacancy (V_{Se}) repair mechanism via DFT calculations and physical analysis (STM, Raman, PL) is also commendable. However, further refinements regarding device operation modes and stability are necessary for practical logic circuit applications.

We appreciate the reviewer's positive comments.

1. **According to the transfer characteristics in Fig. 2f, oxygen doping induces a significant positive shift in the threshold voltage (V_{th}), resulting in depletion-mode (normally-on) operation. Given that enhancement-mode (normally-off) characteristics are essential for minimizing static power consumption in practical CMOS integrated circuits, what strategies do the authors propose to shift the V_{th} toward negative values? Specifically, how can enhancement-mode operation be achieved while preserving the record-high mobility and ultralow contact resistance demonstrated in this work (e.g., through gate work-function engineering or finer control of oxygen concentration)?**

We thank the reviewer for pointing this out. In this work, a one-step doping process is used. To preserve high device performance while offering control over the threshold voltage, a two-step doping approach can be adopted. Specifically, the source/drain and channel regions would be doped separately, with the channel region receiving only a low-level doping to passivate selenium

vacancies. In parallel, moderate doping of the contact regions ensures a sufficiently high carrier concentration for the formation of ohmic contacts. During selective doping, the regions that are not intended to be doped are protected by a mask. Alternatively, independently tuning the dipole of the gate stack can also be employed for synergistic modulation to achieve depletion-mode operation of the device. We thank the reviewer again for the insightful comments and the research on tunable threshold voltage is in progress. We have added a discussion in the revised manuscript as follows:

"The threshold voltage can be tuned either by doping with an appropriate oxygen concentration or by independently tuning the dipole of the gate stack."

2. **The oxygen doping is performed via thermal annealing. How stable is the electrical performance of the OM-WSe_2 devices when exposed to ambient air for a long period?**

We thank the reviewer for pointing this out. The OM-WSe_2 devices remained stable even after being exposed to ambient air for nearly two months, as shown in Supplementary Fig. 27.

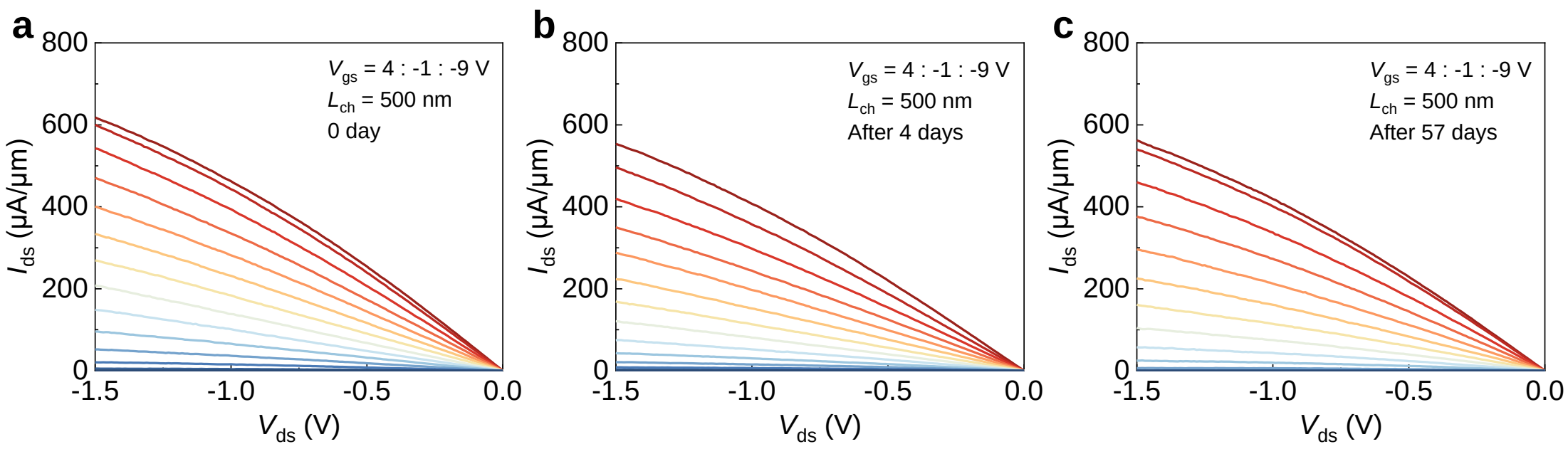

Fig. R7. Stability of OM-WSe_2 transistors. Output characteristics of the WSe_2 transistor (L_{ch} = 500 nm) at once (a) and after 4 days (b) and 57 days (c).

3. **The 'doping last' process shows significantly lower contact resistance than 'doping first'. Is there any evidence that oxygen atoms diffuse underneath the metal contacts to repair defects at the buried**

interface?

We thank the reviewer for pointing this out. Raman and PL measurements were conducted on monolayer WSe_2 covered with 3 nm Pt, and the blueshift of the Raman A_{1g} peak and the PL peak for doping-last confirmed the p-type doping, which proves that oxygen atoms diffused underneath the metal contacts and repaired defects at the buried interface. The doping is possibly through metal grain boundaries or lateral diffusion from the channel to the contact regions.

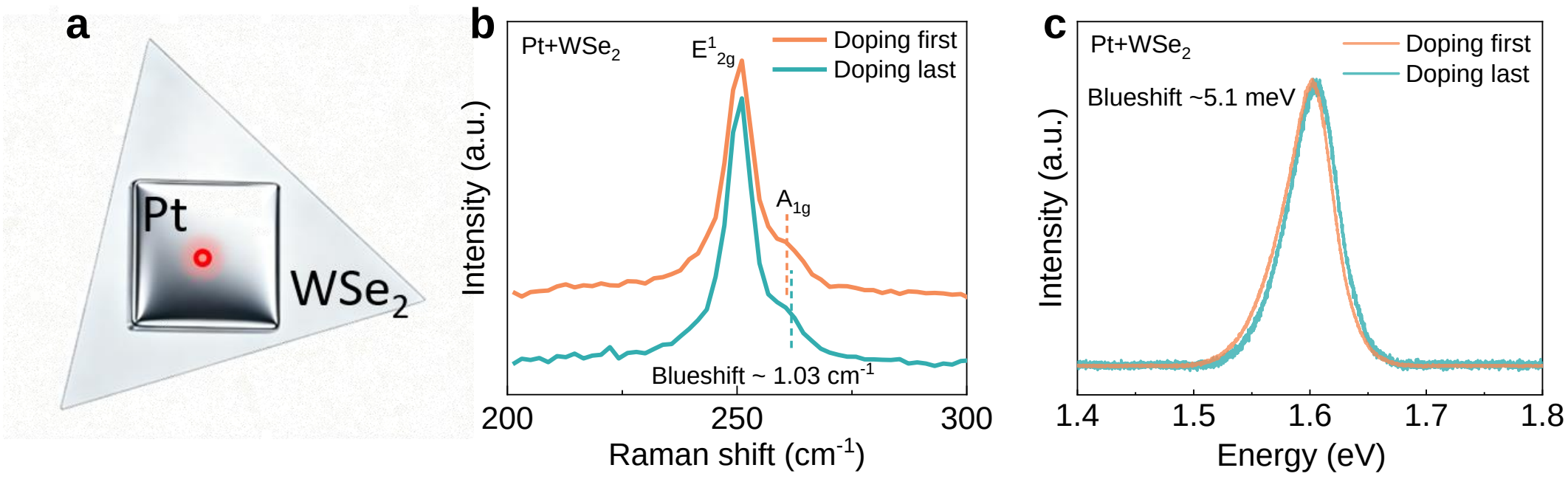

Fig. R8. (a) Schematic diagram of Raman measurement for 3-nm Pt+1L WSe_2, Raman (b) and PL (c) Spectra of Pt+WSe_2 with doping-first and doping-last.

4. **The paper mentions that WSe_2 performance is superior on SiO_2 due to an additional doping effect. Have you observed any degradation in mobility or subthreshold swing (SS) when using high-k dielectrics like HfO_2?**

We thank the reviewer for this important question. In previous studies that employed HfO_2 as the gate dielectric (*Nat. Commun.* 2025, 16, 4160; *Nat. Commun.* 2025, 16, 5649; *ACS Nano* 2025 19 (9), 8916-8925), we observed a certain degree of mobility degradation compared with devices on SiO_2. We attribute this mainly to the transfer-induced damage and the introduction of

defects at the WSe_2/HfO_2 interface during the material transfer process, which act as scattering centers and compromise carrier mobility.

On the other hand, the subthreshold swing (SS) was significantly improved when HfO_2 was used. This is because the high-κ nature of HfO_2 allows a much smaller equivalent oxide thickness (EOT) than that of SiO_2, leading to enhanced gate electrostatic control over the channel. Consequently, the SS, which reflects the steepness of the turn-on characteristic, is notably improved when thin HfO_2 dielectrics are used.

5. While CVD-grown WSe_2 was used, the device-to-device variation across a large scale (e.g., 2-inch wafer) is critical. What is the standard deviation of the mobility and R_c values across the wafer?

We thank the reviewer for pointing this out. As stated in the Methods section, the CVD equipment is a 1-inch tube furnace. We conducted a statistical analysis on the mobility and contact resistance of the same batch of devices and calculated the standard deviation. The mobility and contact resistance of OM-WSe_2 transistors are 189.2 ± 11.2 $cm^2/V \cdot s$ and 165 ± 39 Ω·μm, respectively. These data have been added to the revised manuscript as Supplementary Fig. 12b and Fig. 20.

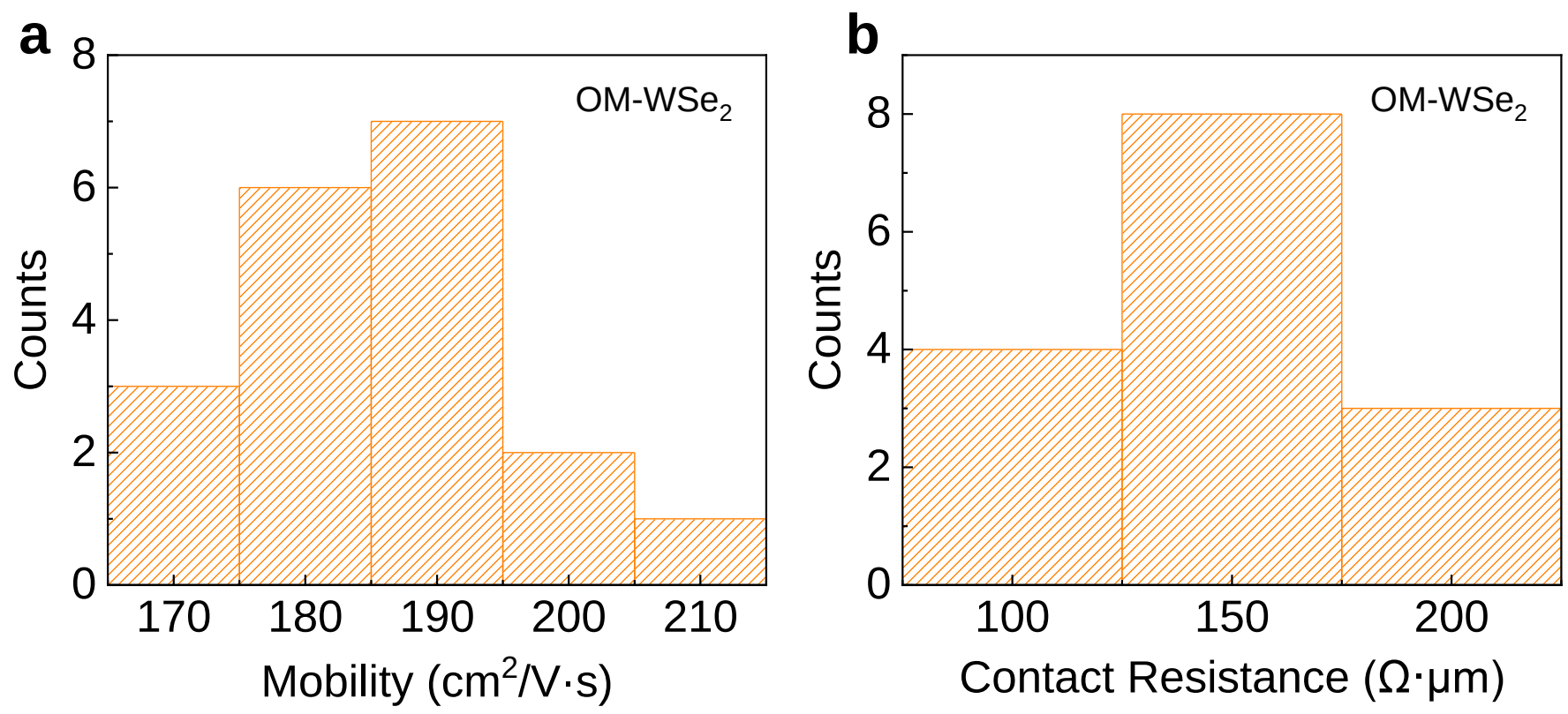

Fig. R9. (a) Statistics of mobility for 19 devices with long channel, (b) Statistics of contact resistance for 15 TLM structures.

~~~~~~
~~~~~~

Reviewer #3 (Remarks to the Author):

Lei Sun et al. proposed a new oxidation method for monolayer WSe_2 to fabricate a ballistic p-type transistor. The method and results appear novel and promising. However, to meet the scientific rigor, reproducibility, and clarity expected by the journal, several issues need to be addressed before its publication.

We appreciate the reviewer's positive comments.

Major points:

1. **A key concern is the reproducibility of the experimental procedure. The device fabrication process should be described in more detail. For example, the manuscript does not explain how the oxygen concentration during annealing (~2x10^-4 mol/L) was measured, either in the main text or in the Methods. Was oxygen introduced only at the maximum annealing temperature of 200 degree C? In addition, what happens if the oxygen concentration during annealing exceeds ~2x10^-4 mol/L? In previous studies involving oxygen doping of WSe_2, the entire WSe_2 layer was typically converted or oxidized (e.g., https://ieeexplore.ieee.org/document/9046453, and so on). In contrast, the mild oxidation used in this work appears quite unique and interesting. From this perspective, it would be helpful if the authors could explain more clearly how pure oxygen annealing can selectively passivate selenium vacancies without converting the entire monolayer. Similarly, details on the Pt S/D electrode deposition are necessary. Under what vacuum level was the deposition performed? What were the source-substrate distance and deposition rate? Is there any passivation layer on top of the Pt electrode?**

We thank the reviewer for pointing this out. The oxygen concentration $C = \frac{n}{V} = \frac{P}{RT}$ was derived by the ideal gas law $PV = nRT$, where *P* is the pressure within the chamber, *V* is the volume of the chamber, *n* is the amount of gas, *R* is the universal gas constant (8.314 J/(mol·K)) and *T* is the absolute temperature. Oxygen was introduced from the beginning of the heating process until the entire annealing process was completed, and the supply was stopped when the sample cooled. When the oxygen concentration during annealing exceeded ~2×10^{-4} mol/L, the mobility of the devices decreased, and the number of defects increased, as shown in Fig. R1. The Pt S/D electrode was deposited under a vacuum of 5e-7 Torr at a rate of 0.3 Å/s. The source-substrate distance is about 65 cm. There is no other passivation layer on top of the Pt electrode except for the 20-nm Au layer. We have added a discussion in the revised manuscript as follows:

"The Pt S/D electrode was deposited under a vacuum of 5e-7 Torr at a rate of 0.3 Å/s. The source-substrate distance is about 65 cm." … "The oxygen concentration C = n/V = P/RT was derived by the ideal gas law PV = nRT, P is the pressure within the chamber, V is the volume of the chamber, n is the amount of gas, R is the universal gas constant (8.314 J/(mol·K)) and T is the absolute temperature. Oxygen was introduced from the beginning of the heating process until the entire annealing process was completed, and the supply was stopped when the sample cooled."

2. **Regarding Fig. 3, the mechanism behind the performance differences between the "doping first" and "doping last" processes is not sufficiently explained. Supplementary Fig. 13 shows a schematic comparison of the two conditions, suggesting that contact doping may differ. However, such discussion is largely missing from the main text. Moreover, in the doping last process, since the contact electrodes act as a mask, oxygen doping is likely to occur more in the**

channel region than underneath the contacts. In that case, one would expect the doping last devices to show inferior performance. Without a materials-level characterization of the contact interface, the current explanation feels not fully convincing.

We thank the reviewer for pointing this out. In the device fabrication, the contact electrodes (Pt) are deposited by electron-beam evaporation at high vacuum with a slow rate to reduce damage. However, this deposition process inevitably introduces some defects (e.g., Se vacancies on the surface of WSe_2) into the underlying WSe_2. Such defects act as scattering centers and increase contact resistance, degrading the overall device performance (*Nat. Electron.* 2024, 7, 638–645).

For the doping first process, oxygen annealing is performed before metal deposition. Although the pre-existing Se vacancies in the channel and contact regions are effectively passivated by oxygen, the subsequent metal deposition creates new defects at the contact interface. These newly generated defects are not repaired because no further annealing is carried out after metallization. Consequently, the contact region in doping-first devices still suffers from defect-induced degradation, limiting the achievable contact quality.

In the doping last process, the oxygen annealing is performed after the contact electrodes have been fabricated. Raman and PL characterization (shown in Fig. R10) were performed on WSe_2 samples with metal contacts before and after oxygen annealing. The results clearly demonstrate that despite the presence of the top metal layer, the blueshift of the Raman A_{1g} peak and the PL peak for doping-last confirmed the p-type doping in the contact region, indicating that oxygen species can indeed laterally diffuse from the contact edges and heal Se vacancies underneath the contacts. Moreover, the doping-last devices exhibit better electrical performance than that of doping first devices (shown in Fig. R11). This observation strongly suggests that the

contact regions are effectively passivated in the doping last case, consistent with the Raman and PL results.

Therefore, contrary to the expectation that the metal mask would block doping, the doping last process actually benefits from the post-metallization annealing, which passivates both the channel defects and the metal-induced damage at the contact region. We have added a discussion in the revised manuscript as follows:

"Raman and PL spectra confirm oxygen doping in the contact regions, which likely occurs through metal grain boundaries or lateral diffusion from the channel (Supplementary Fig. 18)."

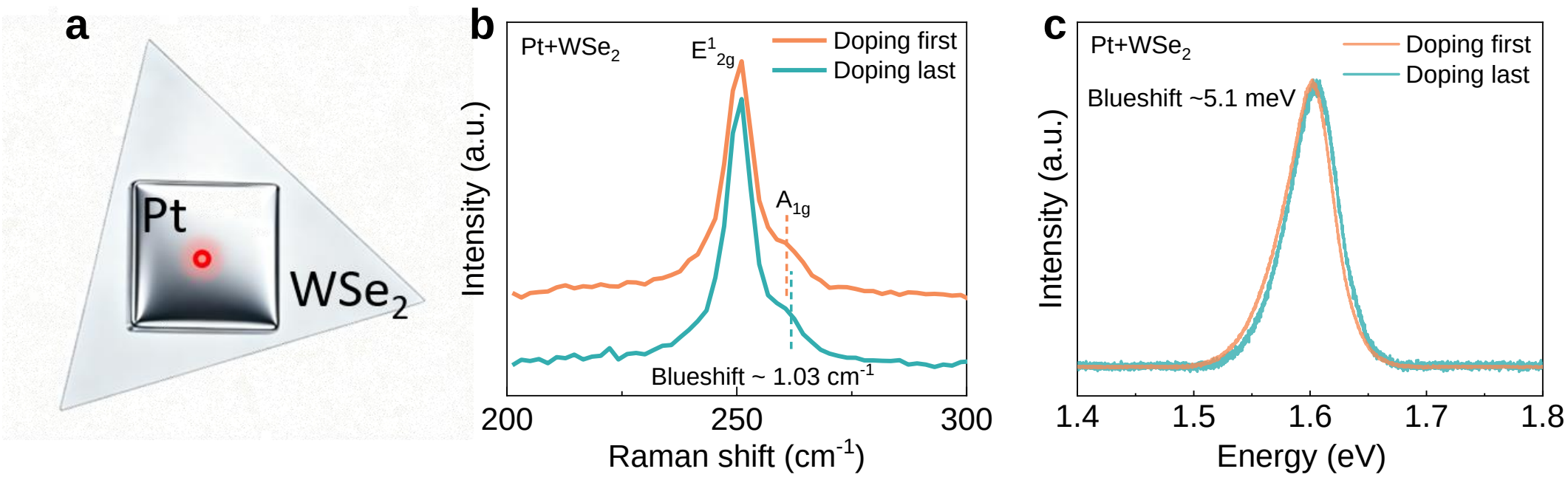

Fig. R10. (a) Schematic diagram of Raman measurement for 3-nm Pt+1L WSe_2, Raman (b) and PL (c) Spectra of Pt+WSe_2 with doping-first and doping-last.

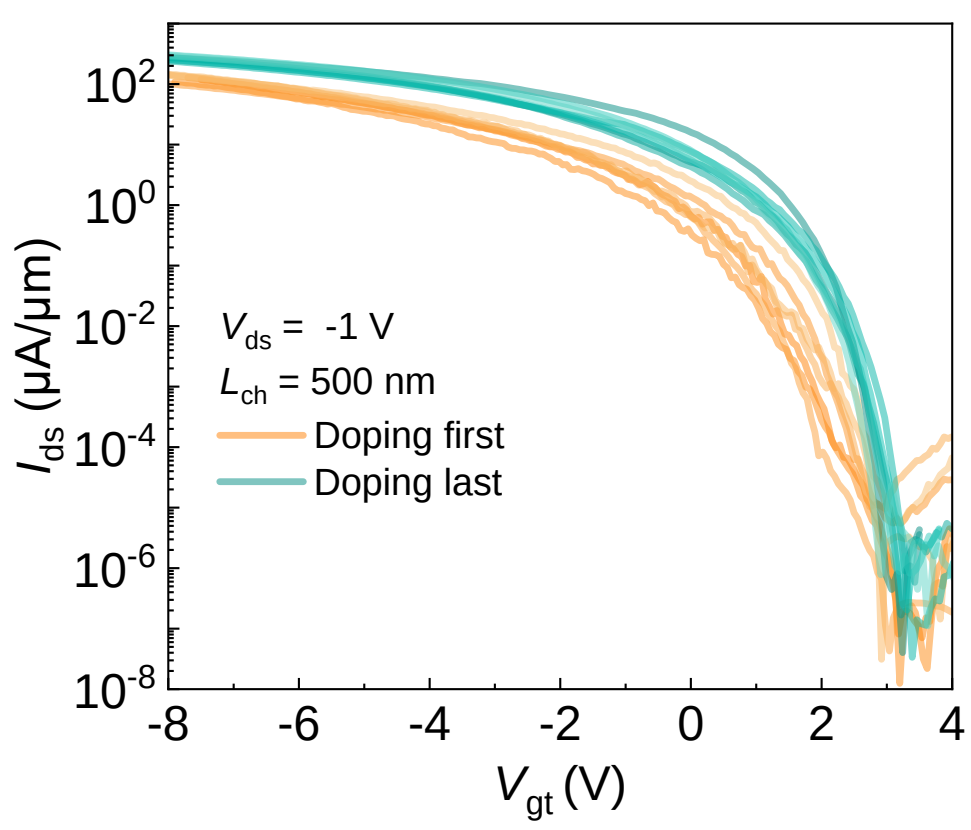

Fig. R11. Transfer characteristics curve statistics data of devices with doping first and doping last.

3. **It would also strengthen the manuscript to investigate the temperature dependence of device properties. From the conductivity trend, the device seems to exhibit a negative thermionic emission barrier for V_g < 2 V and undergoes a metal-to-insulator transition (MIT), as shown in Fig. S15. Compared to previously reported WSe_2 systems, is the MIT less or more pronounced? How does the mobility change with temperature? Is the transport limited by contact scattering?**

We thank the reviewer for pointing this out. From temperature-dependent transfer characteristics (Fig. R12(a)), we do observe a clear MIT. Compared to previously reported WSe_2 devices, the MIT observed in our monolayer devices is pronounced because the carrier density (~ 1.4×10^{12} cm^{-2}) is lower than that of the previous study (*Adv. Electron. Mater.* 2022, 8, 2200046).

From the measured temperature-dependent transfer characteristics in the linear regime, we extracted the field-effect mobility as a function of temperature. As shown in Fig. R12(b), the mobility increases when the temperature decreases from 300 K to 220 K. This behavior indicates that phonon scattering is the dominant mobility-limiting mechanism in this temperature range. At room temperature, the lattice vibrations (phonons) are strong and scatter the carriers frequently, resulting in lower mobility. As the temperature is lowered, phonon population decreases, and the phonon-scattering rate is suppressed, allowing the mobility to increase. These data have been added to the revised manuscript as Supplementary Fig. 28. Although the contact resistance is low in this work, it is still higher than that of quantum limit (*Nature Electronics*, 8, 96 (2025)). Therefore, we think the transport is limited by contact scattering. We have added a discussion in the revised manuscript as follows:

"The presence of a metal-to-insulator transition (MIT) at a low carrier density of 1.4×10^{12} cm^{-2} indicates a low defect density after oxygen doping (Supplementary Fig. 28)"

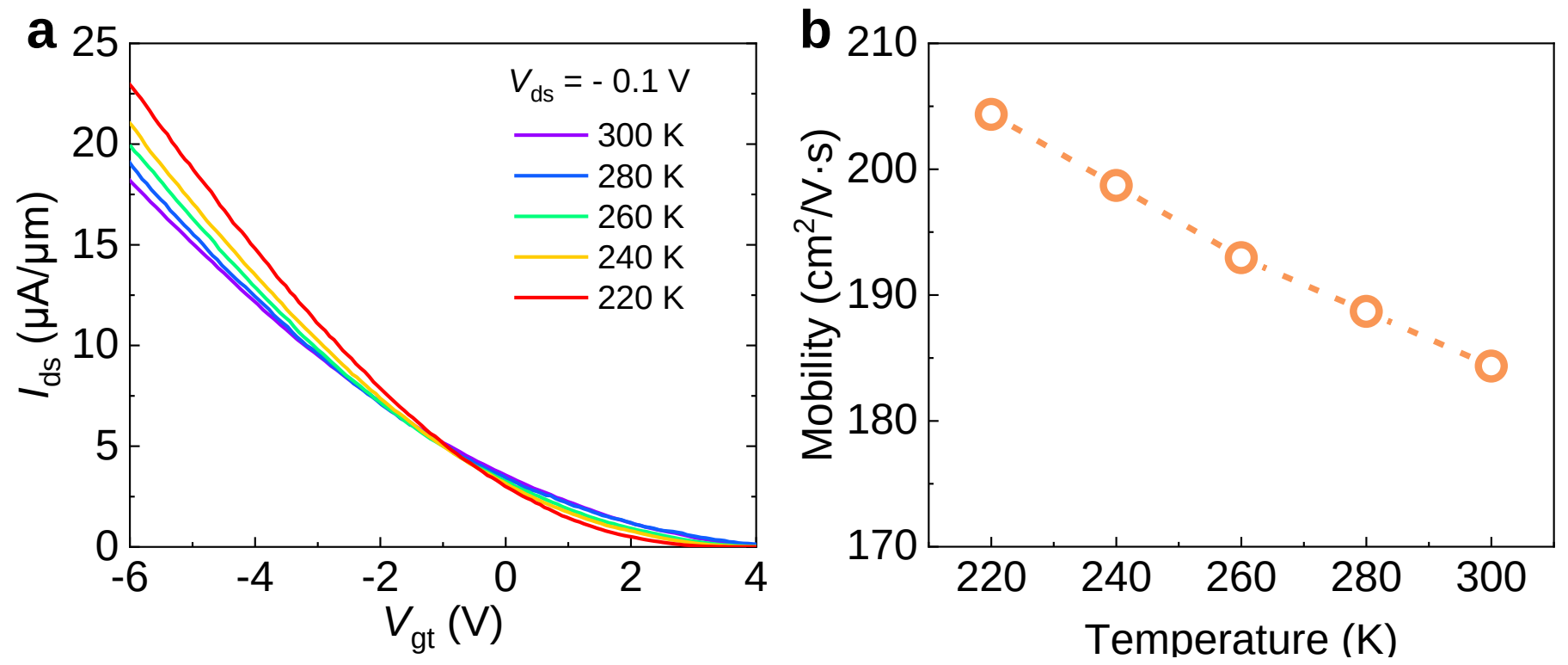

Fig. R12. Temperature-dependent transfer characteristics (a) and mobility (b) of the device.

4. **To determine whether the WSe_2 FETs truly operate in the ballistic regime, it is essential to extract the saturation velocity and examine its temperature dependence. This evaluation is currently missing. Furthermore, ballistic transport should be demonstrated through channel-length scaling that shows reduced scattering as the device dimensions shrink. Such evidence is not presented in the manuscript.**

We thank the reviewer for pointing this out. We extracted the temperature dependent injection velocity as shown in Fig. R13(a). At 300 K, the extracted ballisticity (v_{inj}/v_T) is ~0.81 (as mentioned in the main text), indicating that carrier transport is still affected by backscattering. As the temperature is reduced to 240 K, the ballisticity increases (to ~0.92), reflecting a transition toward more ballistic transport. This increase can be primarily attributed to the reduction of phonon scattering. Below 240 K, however, the extracted ballisticity decreases slightly. This behavior suggests that additional scattering mechanisms become increasingly important at low temperature, such as Coulomb-related scattering and interface-disorder effects, partially offsetting the benefit of suppressed phonon scattering.

In the short-channel regime, the drain current increases only weakly as the channel length is reduced as shown in Fig. R13(b). This behavior indicates that

the overall device resistance is no longer dominated by the channel scattering-limited component. Instead, the total resistance becomes increasingly governed by the contact resistance and the ballistic injection resistance, both of which are independent of L_{ch}. The weak dependence of I_{ds} on L_{ch} therefore reflects a reduced probability of scattering events within the channel, implying that carrier transport approaches the quasi-ballistic or ballistic regime where scattering plays a minor role. These data have been added to the revised manuscript as Supplementary Fig. 29. We have added a discussion in the revised manuscript as follows:

"Channel-length scaling that shows reduced scattering as the device dimensions shrink (Supplementary Fig. 29)."

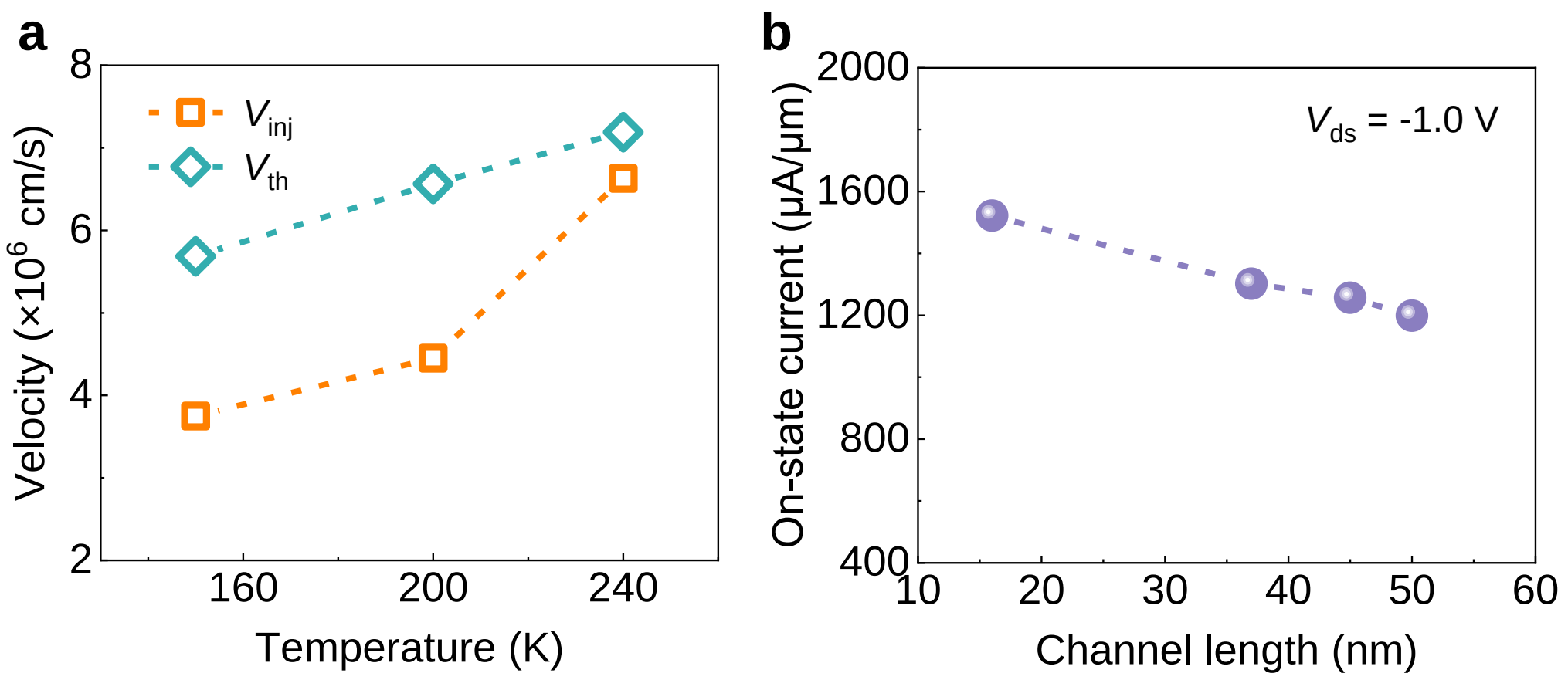

Fig. R13. (a) Temperature-dependent carrier injection velocity and thermal velocity, (b) Channel-length dependence of current in short-channel devices.

Minor points:

5. The notation for gate voltages is not consistent across the figures. For example, V_{gt}, V_{bg}, and other symbols are used in different panels.

We thank the reviewer for pointing this out. All the V_{bg} have been changed to V_{gs}. The overdrive voltage V_{gt} ($V_{gt} = V_{gs} - V_{th}$) has also defined in Fig. 3c.

6. In Figs. 2f and 2h, the information on V_{ds} and V_{gs} is missing. The figure captions should be revised.

We thank the reviewer for pointing this out and this has been revised accordingly in the revised manuscript.

7. For Figs. 3d and 3e, the V_g conditions are not provided.

We thank the reviewer for pointing this out. The contact resistance is extracted at a carrier density of 1.0 x 10^{13} cm^{-2}, which has been added in the Fig.3d. The gate voltage is 1.1 V for doping first and 2 V for doping last, which has been added in the Fig. 3e.

8. In Supplementary Fig. 16, I suggest quantifying the linearity as a function of V_g and providing the corresponding figures (e.g., linearity metrics) rather than only qualitative plots.

We thank the reviewer for this suggestion. This method is qualitative to analyze the contact quality as shown in the previous study (Nature communications, 16, 4018 (2025)). As shown in Supplementary Fig. 22, the I_{ds}–V_{ds} curves remain linear even at 4 K, indicating excellent contact quality in this work (nonlinearity is 0). Therefore, different plots don't affect our conclusion.

9. On line 252-254, "To further evaluate the potential of WSe_2 pFETs with doping last, we scaled the channel length down to 16 nm on monolayer WSe_2, which channel length was confirmed by high-resolution cross-sectional transmission electron microscopy (TEM) image (Supplementary Figs. 17–18)." However, there is no actual TEM image shown; only EDS data are presented in Fig. S18. It also appears that the current Fig. S17 may have been placed incorrectly.

We thank the reviewer for pointing this out and the TEM image has been added accordingly in the revised manuscript. This data has been added to the revised manuscript as Supplementary Fig. 23a. To avoid the confusion, original Fig. S17 has been deleted.

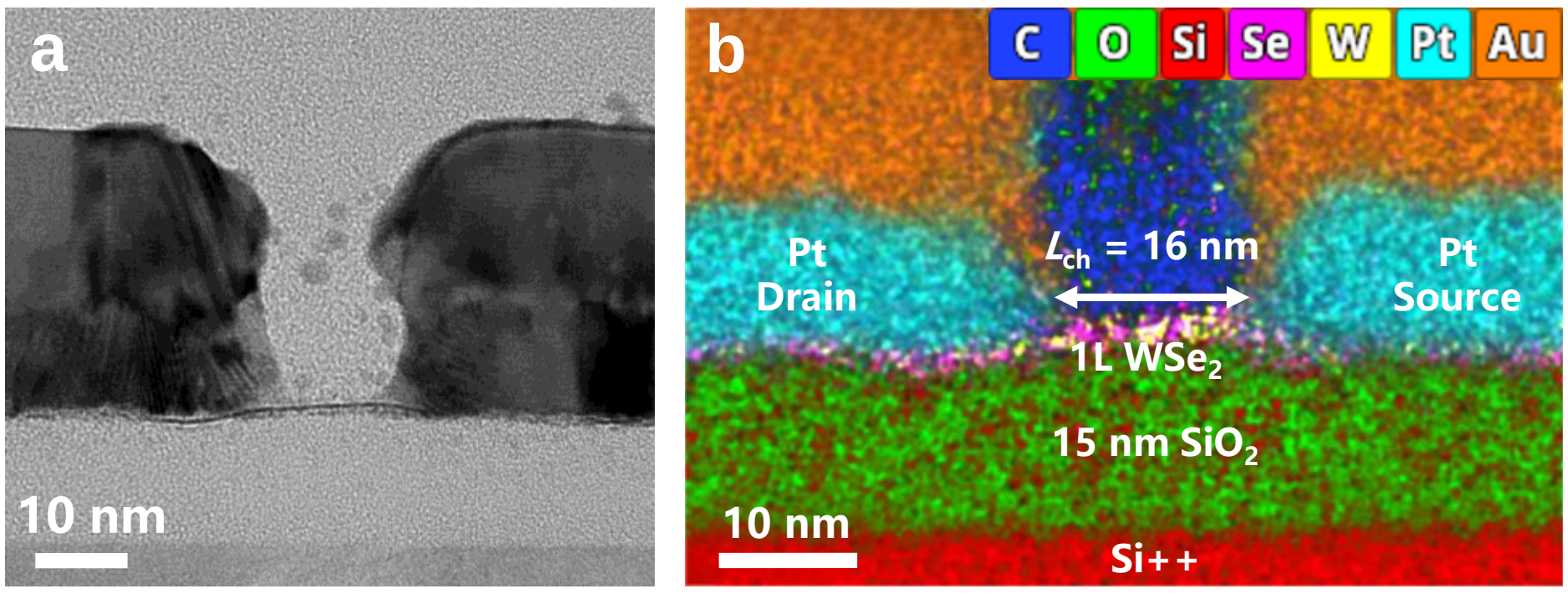

Fig. R14. TEM (a) and EDS mapping (b) of the ballistic monolayer WSe_2 transistor. The channel length of the device is approximately 16 nm.

.++Point-by-point response to reviewers' comments

To the reviewers:

We are grateful to the reviewers for their time and very helpful comments. In response, we have included more experimental results and analysis. We have made changes to the text, and accordingly, the manuscript has been improved in several respects. In the revised manuscript, we have incorporated all the suggested changes and clarifications. We believe that we have been able to address all the concerns of the reviewers.

The key changes in the modified manuscript are as follows:

1. C–V curves before and after the post-annealing process
2. Subthreshold swing (SS) data before and after oxygen annealing for the HfO_2-gated devices and native high-k interface traps (D_{it})
3. Threshold voltage (V_{th}) shifts as a function of channel length (L_{ch})

In summary, we greatly appreciate the reviewer's insightful comments and valuable suggestions, which helped us clarify many issues in the original manuscript and strengthened the manuscript with these new discussions. Thus, by incorporating these new data and analyses, we believe that we have now addressed the reviewer's concerns.

The responses to the reviewer's comments are listed below.

~~~~~~

Reviewers' comments:

Reviewer #1 (Remarks to the Author):

The authors have addressed most of the comments. However, they have
~~~~~~

not yet provided C-V measurements of the dielectrics both after growth and after the post-annealing process. This information is important because changes in the dielectric capacitance can directly affect the extracted mobility values.

We appreciate the reviewer's positive comments, and the article has been revised to comply with the reviewer's suggestions.

The detailed questions are as follows:

1. **The authors should provide the C-V data used for mobility extraction and clearly describe the step-by-step procedure used to calculate the mobility, including how the gate capacitance was determined, which transfer-curve regime was used, and whether the same capacitance value was assumed before and after post-annealing.**

We thank the reviewer for the thorough and constructive comments regarding the mobility extraction procedure. Below, we provide the requested C-V data, a detailed step-by-step description of our extraction method, and clarifications regarding the gate capacitance used before and after post-annealing.

We fabricated a metal oxide semiconductor capacitor (MOSCAP) on purchased SiO_2/Si substrates both with and without a post-annealing process to perform C–V measurements. The measured C–V curves are provided below (Fig. R1). Owing to the heavily doped nature of the substrate (which acts as a degenerate semiconductor), the device remains in the accumulation regime across the entire applied gate-voltage sweep range and cannot be driven into depletion or inversion. Consequently, the measured capacitance does not decrease to zero at zero or positive bias. The gate oxide capacitance (C_{ox}) was determined directly from the accumulation plateau capacitance of the MOSCAP.

The mobility was extracted according to the formula $\mu = \frac{g_m L_{ch}}{W_{ch} C_{ox} V_{ds}}$, where g_m is the transconductance, L_{ch} is the channel length, W_{ch} is the channel width, C_{ox} is the gate oxide capacitance per unit area (here, C_{SiO2} = 0.26 μF/cm^2), and V_{ds} is the drain-source voltage. We specifically used the linear transfer curve regime (V_{ds} = -0.1 V) for this extraction to avoid series resistance and short-channel effects associated with the saturation regime.

As the postannealing process reduces the interfacial trap density and causes structural densification of the gate dielectric, which slightly reduces its physical thickness or increases its permittivity, the observed slight increase after annealing is physically plausible. Regardless of the origin, the value of C_{ox} does not affect the conclusion that the mobility increases substantially after annealing. In this paper, owing to the numerous annealing conditions, a uniform C_{ox} value is adopted for extracting the mobility to facilitate comparison.

The data have been updated in Supplementary Fig. 12, and we have added a discussion in the revised manuscript as follows:

"The areal capacitance of the gate dielectric (C_{ox}) is 0.26 μF/cm^2. Since the variation in C_{ox} before and after annealing is negligible (as shown in Supplementary Fig. 12), a constant C_{ox} value is adopted for all mobility extractions to ensure a fair comparison."

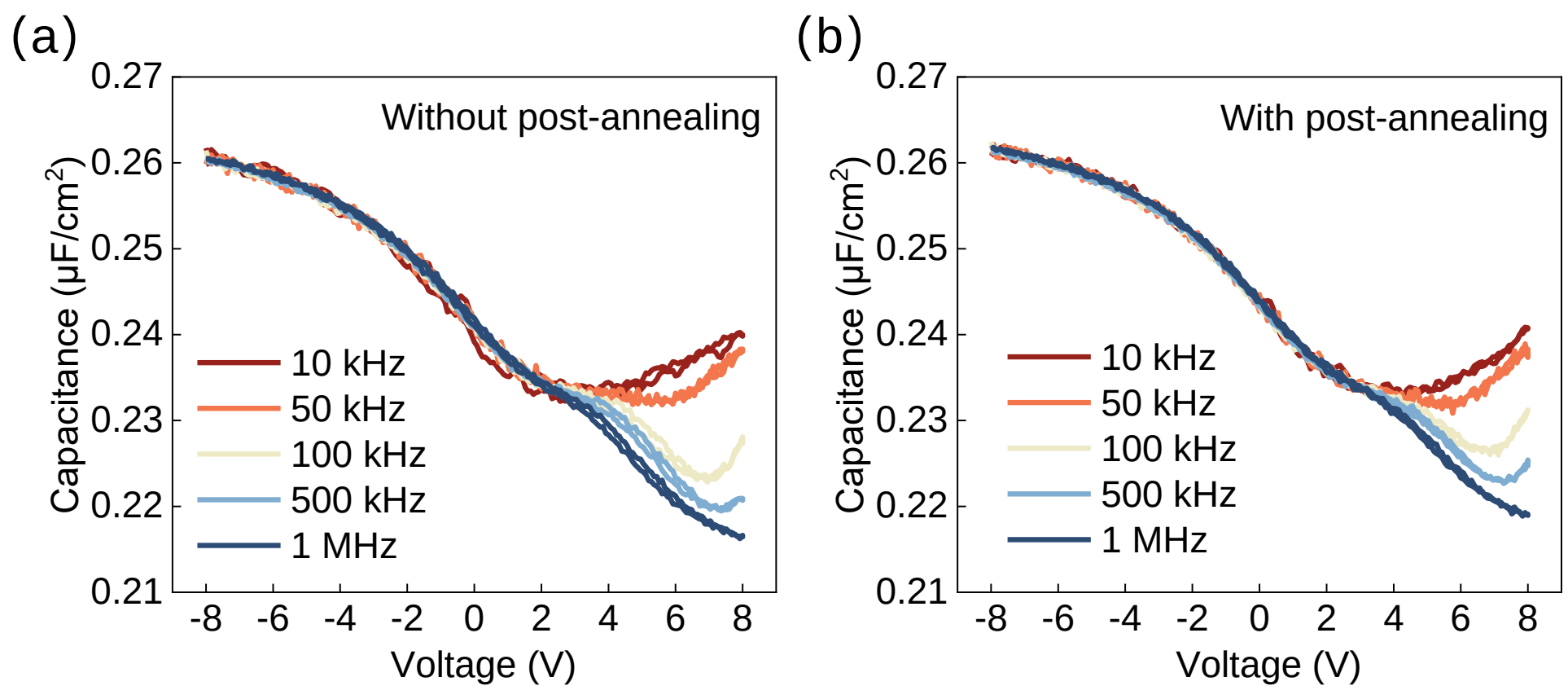

R1. C–V curves of purchased SiO_2/Si substrates without (a) and with (b) post-annealing.

~~~~~~

<u>Reviewer #2 (Remarks to the Author):</u>

This manuscript presents an intriguing approach to mitigating the chronic issue of high selenium vacancy density in monolayer WSe_2 via a controlled oxygen-moderate annealing process, which, combined with a full-contact configuration, delivers exceptional hole mobility and ultralow contact resistance. The authors have made commendable efforts to address the first-round review comments, particularly by introducing Raman and PL profiles of a 3 nm Pt-covered WSe_2 layer to justify their oxygen diffusion mechanism. However, to robustly validate the scalability for practical CMOS integrated circuits and the physical soundness of the proposed diffusion model, several critical open questions must be resolved. Specifically, potential performance degradation factors during device scaling and the underlying scattering mechanisms require a deeper investigation. Therefore, I recommend further clarification on the following points.

We appreciate the reviewer's positive comments, and the article has been revised to comply with the reviewer's suggestions.

1. **Please provide quantitative subthreshold swing (SS) data before and after oxygen annealing for the HfO_2-gated devices to clarify how the oxygen incorporation affects remote Coulomb scattering and native high-k interface traps (D_{it}).**

We thank the reviewer for pointing this out. Following the reviewer's suggestion, we extracted the quantitative SS data before and after oxygen annealing, as shown in Fig. R2 (also in Supplementary Fig. 25). The device exhibits a significant reduction in the amount of SS after oxygen annealing. This
~~~~~~

improvement in the SS (from ~211 to ~96 mV/dec) directly indicates a substantial decrease in the net trap density near the interface region.

For native high-k interface traps (D_{it}), the reduction in SS after annealing is direct evidence of interface state passivation. Oxygen annealing effectively allows oxygen atoms to diffuse to the HfO_2/Si interface, terminating the dangling bonds and reducing D_{it} from ~4.45×10^{13} eV^{-1} cm^{-2} to ~1.06×10^{13} eV^{-1} cm^{-2} (as calculated by $SS = \frac{k_B T}{q} ln(10)(1 + \frac{qD_{it}}{C_{ox}})$). This passivation reduces trap-assisted recombination, thereby steepening the SS.

For remote Coulomb scattering (RCS), in addition to interface passivation, oxygen annealing plays a crucial role in suppressing the RCS. The as-deposited HfO_2 films typically contain a high density of oxygen vacancies (V_O) acting as charged bulk traps. These bulk traps are located physically close to the channel; their long-range Coulomb potential scatters the channel carriers, degrading the subthreshold slope (especially at low drain current levels). After oxygen annealing, these oxygen vacancies are effectively filled, reducing the fixed bulk charge density. The reduction in these remote charged centers weakens the Coulomb scattering potential in the channel, which is corroborated by the improved carrier mobility and the steeper SS observed in the linear region.

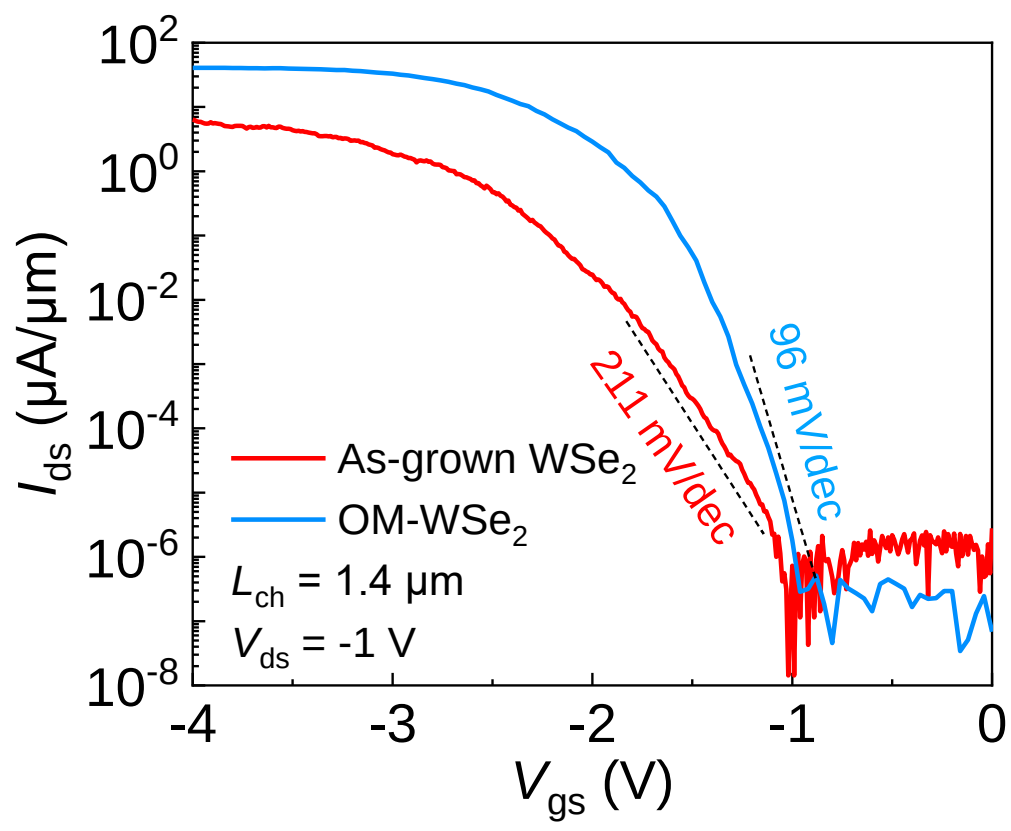

Fig. R2. Transfer characteristics of WSe_2 transistors without and with O_2 doping on HfO_2 gate dielectrics.

2. **If oxygen laterally diffuses from the contact edges into the channel during the 'doping-last' process, it could create a doping concentration gradient. Do you have experimental data tracking V_{th} shifts as a function of L_{ch} to rule out this profile non-uniformity?**

We thank the reviewer for pointing this out and agree that clarifying the diffusion pathway is essential. We extracted the V_{th} shifts as a function of L_{ch}, as shown in Fig. R3. As expectation, the V_{th} of the transistors shifts toward positive as L_{ch} decreases due to short-channel effect.

However, we wish to emphasize a key distinction regarding the underlying mechanism: in our "doping-last" process, oxygen incorporation (or the relevant oxygen species) is believed to proceed via metal grain boundaries or lateral diffusion from the surrounding regions toward the contact areas, rather than diffusing from the contact edges into the channel. Owing to the extended two-hour annealing duration, the resulting doping profile is highly uniform. Second, the contact area of WSe_2 transistors with different L_{ch} is all the same. The doping concentration in contact region should be the same. Therefore, we can eliminate potential concentration gradient. We have added a discussion in the revised manuscript as follows:

"… from the regions surrounding the contacts."

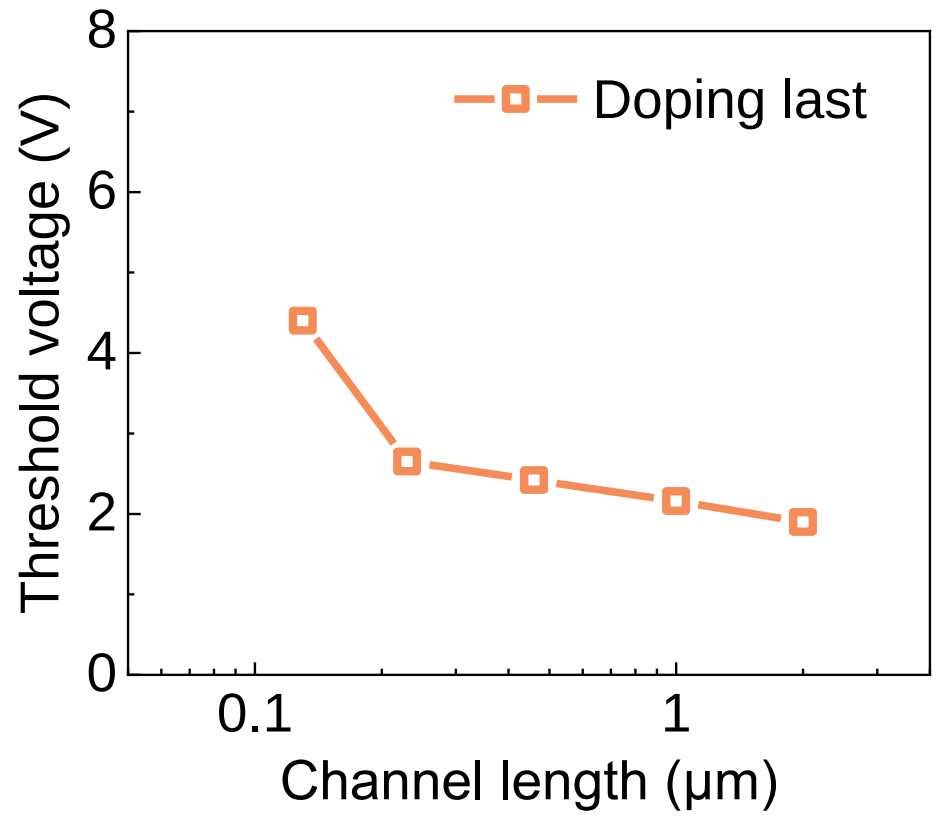

Fig.R3. Threshold voltage of devices with doping-last shift as a function of channel length.

~~~~~~

<u>Reviewer #3 (Remarks to the Author):</u>

The authors have revised the manuscript thoroughly and have addressed all the issues I raised. I believe this work is both highly timely and novel. I therefore recommend its publication in Nature Communications.

We appreciate the reviewer’s positive comments.
~~~~~~

.++Point-by-point response to reviewers' comments

To the reviewers:

We are grateful to the reviewers for their time and very helpful comments. In response, we have further modified our statement. We believe that we have been able to address the remaining concern of the reviewer #2.

The responses to the reviewer's comments are listed below.

~~~~~~

Reviewers' comments:

Reviewer #1 (Remarks to the Author):

The authors have addressed my comments.

We appreciate the reviewer’s positive comments.

~~~~~~

Reviewer #2 (Remarks to the Author):

The authors have provided additional clarification regarding the possible oxygen diffusion pathway. However, the current V_{th} versus L_{ch} analysis does not fully rule out a possible channel-length-dependent doping profile, since V_{th} itself is influenced by short-channel effects.

It would therefore be helpful if the authors could provide the annealing-induced threshold-voltage shift, $\Delta V_{th} = V_{th,after} - V_{th, before}$, or an equivalent

analysis that more directly evaluates the uniformity of oxygen doping. If such data are not available, the statement that the doping profile is highly uniform should be moderated accordingly.

We appreciate the reviewer’s thoughtful comment. As data directly evaluating the uniformity of oxygen doping are currently unavailable, we have moderated our statement accordingly. To ensure scientific rigor, we have removed the discussion regarding the doping profile from the revised manuscript.
